# Supplementary material for: The Shape of Human Red Blood Cells Suspended in Autologous Plasma and Serum
Source: Cells. 2022 Jun 16;11(12):1941. doi: 10.3390/cells11121941 (PMC9222013; doi:10.3390/cells11121941)
Supplement: Supplementary file 1 [file cells-11-01941-s001.zip › cells-1742284-supplementary/Document S1 - Supplmentary information.pdf]

Supporting Information to

The Shape of Human Red Blood Cells Suspended in Autologous Plasma and Serum

Thomas M. Fischer

Department of Experimental Physics, Saarland University, Campus E2 6, 66123 Saarbrücken, Germany; phone: +49 160 2293318, email: thmfischer@gmail.com

and

Laboratory for Red Cell Rheology, Krummer Weg 20, Herzogenrath, Germany

1 Shape change induced by albumin

Red cells were observed with an 100/1.30 interference contrast T objective (Leitz, Wetzlar, Germany). For illumination, a custom made light source (LED 515 nm) was used.

The experiment started by filling a transparent chamber with a suspension of RBCs suspended in PBS. Bottom and top of the chamber were made of glass. The glass effect transformed the cells into spheres decorated with spicules, so-called echinocytes type 3 (E3) [1].

In a second step, PBS supplemented with bovine albumin (2 g%) was added in such a way that both liquids were in contact without mixing. Due to diffusion of the albumin, its concentration slowly increased at the location of the RBCs. Albumin is a stomatocytic agent. As such it decreases the spontaneous curvature of the membrane. This decrease transforms echinocytes into discocytes and eventually into stomatocytes. Figure S1 shows selected frames of such a sequence.

For technical reasons, it was not possible to capture the cell in the E3 stage. The first image shows an echinocyte type 2 (E2) which still has spicules but its overall shape is flat. The whole sequence is shown in a movie (Supplementary Materials, Movie 1). The movie is in time lapse mode. The contraction in time is 27 fold.

The stable edge-on orientation during this sequence is explained as follows. After filling the chamber and subsequent sedimentation, the E3 attached with the tip of some spicules to the bottom. Obviously corresponded these spicules to a portion of the rim of the former (before filling the suspension into the chamber) and later (Figure S1 at 127s) biconcave disc. The attachment of the rim to the bottom of the chamber is suggested having prevented the rotation into a face-on orientation.

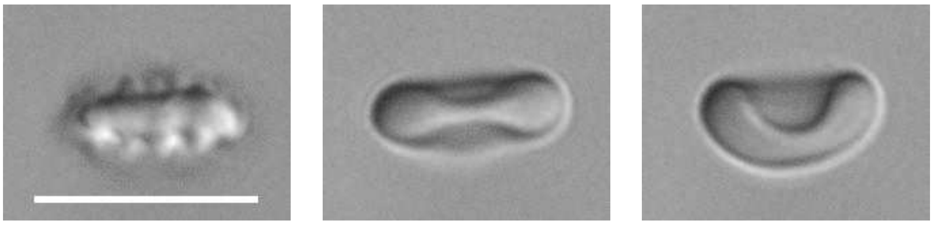

0 s   127 s   427 s

Figure S1: Selected frames of a movie (Supplementary Materials, Movie 1). The times correspond to the actual time in the experiment. The scale bar corresponds to 10 µm.

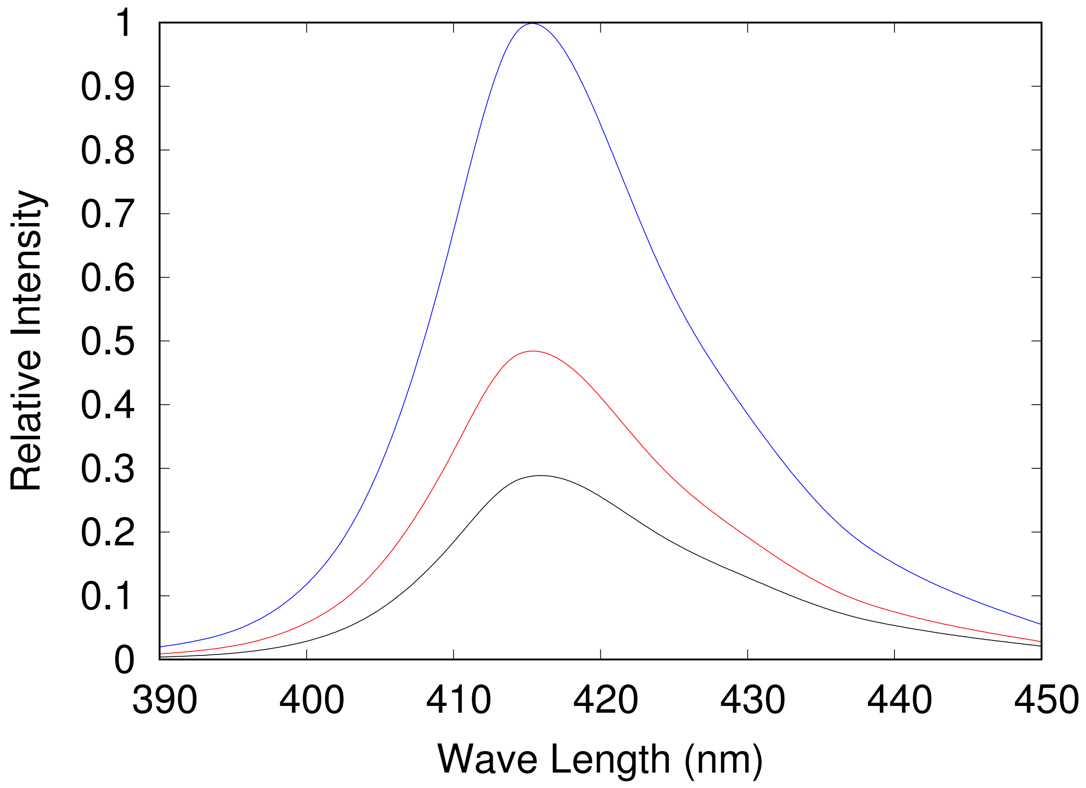

Figure S2: Measured and calculated intensities versus wave length. Blue: the intensity delivered by the LED. Red: the expected intensity after passing the filter N1.7. Black: the intensity expected to be recorded by the camera chip.

2 Calibration of the camera response

Figure S2 (blue) shows the spectrum of the LED as supplied by the manufacturer. To dim the illumination by the LED, a set of six neutral gray filters was used. The transmittance through these filters was measured as a function of incident wave length using the spectrometer AVASPEC-ULS2048CL-EVO-RS-UA (Apeldoorn, The Netherlands). Multiplication of the respective functions with the spectrum of the LED results in the spectrum of the transmitted light. In Figure S2 the resulting curve is shown in red for the filter called N1.7. The red curve in turn was multiplied with the spectral sensitivity of the camera chip resulting in the black curve in Figure S2. The area under the black curve was taken as a relative measure of the expected camera response.

Gray values (GVs) were observed in the background of a microscopic image prepared as described in Section 2.2. Without dimming, the intensity of the illumination was chosen as bright as possible but with the background peak completely contained in the histogram of GVs. A series of images was recorded placing the neutral gray filters in the optical path. In these images, a region of 285x129 pixels was selected which is large enough to average out oscillations of GV. In Figure S3 the average GVs inside this region are plotted versus the expected response of the camera. The linearity warrants to use GVs directly as measures of the reduction in transmitted light by the cytoplasmic hemoglobin (Hb).

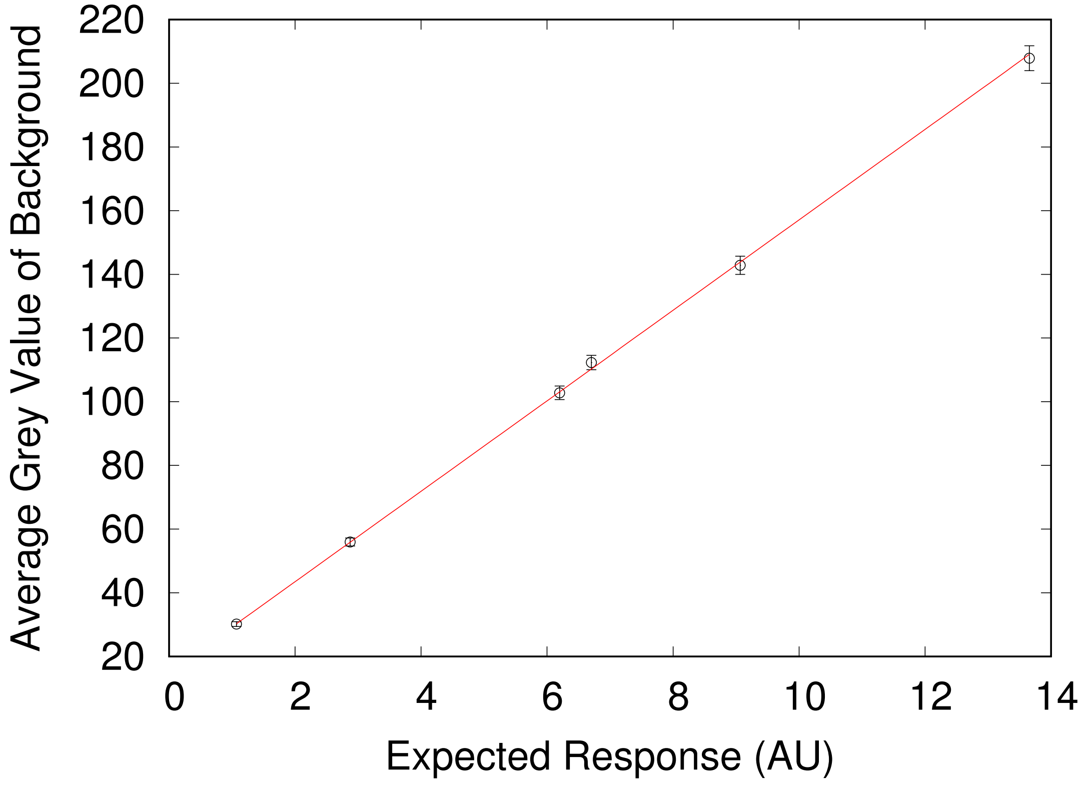

Figure S3: Measured GVs versus calculated intensities. Black: data with SD. Red: linear regression.

3 Accounting for the absorption of plasma

The situation in the experiment is described by a serial application of Equation 1:

$$I_\mathrm{p} = I_0 e^{-\mu_\mathrm{p} d_\mathrm{p}}; \qquad I_1 = I_\mathrm{p} e^{-\mu_\mathrm{r} d_\mathrm{r}}, \tag{1}$$

where μ is the absorption coefficient and the indices p and r indicate plasma and RBC, respectively. After combining the two equations:

$$\ln \frac{I_1}{I_0} = -\mu_\mathrm{p} d_\mathrm{p} - \mu_\mathrm{r} d_\mathrm{r}. \tag{2}$$

With $d_\mathrm{p} = \mathrm{f}_1 d_\mathrm{r}$ and $\mu_\mathrm{p} = \mathrm{f}_2 \mu_\mathrm{r}$, where f_1 and f_2 are numerical factors we obtain:

$$\ln \frac{I_1}{I_0} = -\mu_\mathrm{r} d_\mathrm{r} (\mathrm{f}_1 \mathrm{f}_2 + 1) \tag{3}$$

and finally the analog to Equation 2:

$$\frac{\ln(\mathrm{GV}_\mathrm{ra}) - \ln(65535)}{\ln(\mathrm{GV}_\mathrm{rb}) - \ln(65535)} = \frac{\mu_\mathrm{r} d_\mathrm{ra} (\mathrm{f}_1 \mathrm{f}_2 + 1)}{\mu_\mathrm{r} d_\mathrm{rb} (\mathrm{f}_1 \mathrm{f}_2 + 1)} = \frac{d_\mathrm{ra}}{d_\mathrm{rb}}. \tag{4}$$

4 Image processing

The backbone of the code are shell scripts running under debian linux. Inside the scripts various jobs were assigned to the following freely available software: imagej [2], gawk, gnuplot, LaTeX, and imageMagick. The following steps were performed sequentially:

1. The frames stored on the hard disk were binarized using the default method of imagej for thresholding. This operation results in black objects on white background. An example of a single cell shows Figure 2b.

2. Small and large objects were excluded from further processing. Small objects were cells completely out of focus because they stuck at the "ceiling" of the chamber or remnants of cells interpreted later

as the result of pitting events in the spleen. Large objects were RBC aggregates or several cells so close to each other that the thresholding routine recognized them as a single object.

3. Each of the remaining objects was cropped resulting in a small image containing the object in its center and possibly parts of neighboring cells at the edge of the image.

4. The background was set to white using the plugin "Non-uniform Background Removal" of imagej. To this end 8 regions of interest (ROIs) were defined in a circle around the central object serving as background samples (Figure 2a). ROIs not representing the actual background but regions outside the frame or close to neighboring cells were removed. The plugin finds a plane through the GVs of the remaining ROIs. Finally, all GVs of the image were corrected by the respective GV of that plane using

$$GV_{\text{corrected}} = \frac{GV_{\text{image}} \cdot 65535}{GV_{\text{plane}}}, \tag{5}$$

where 65535 corresponds to white in 16-bit images required by the plugin.

5. Aggregated doublets or triplets passed the filter step 2. The mean GV of the 16-bit images inside the contour of the digitized objects was calculated and the values displayed as a histogram. These histograms showed two peaks. The peak with the lower GV corresponded to remaining aggregates. The respective objects were removed.

6. In some images neighboring objects were so close that they might disturb the determination of the cell profiles (Figure 3). To remove the respective images, the threshold procedure (step 1) was run with an increased value of the threshold. As a consequence, the objects increase in size, close neighbors coalesce with the cell in the center, and the combined object is removed thanks to the feature that excludes objects touching the rim.

7. To eliminate cells with bad focus, a gradient image was generated from the images converted back to 8-bit. High GVs in these images correspond to steep gradients. From each image, only those pixels were extracted that lined the contour of the digitized images (Figure 2b) on both sides. The positions of these pixels were converted to polar coordinates resulting in a list of GVs along the contour.

   RBCs with lower Hb concentration have higher mean GVs. At same 3D shape they have smaller gradients. To compensate, the gradients were renormalized to values that would prevail if the mean GV of the respective RBC was 0.4 the GV of white (Figure S4).

   The resulting data were smoothed using a spline. With two thresholds one for the average GV and another one for the minimum GV of this spline, most images with unfocused cells were eliminated. Since the contour and the maximum gradient did not always coincide, another filter followed (step 8).

8. To eliminate remaining unfocused cells, the GVs of the gradient image were recorded along a ray through the center of the cell and including an angle α with the horizontal. The GVs along this ray had a local maximum at the location of the cell border. The three highest GVs of each ray were averaged. This averaged value was recorded for 180 equally spaced angles α resulting in a list of GVs along the contour. As in step 7, these data were renormalized, smoothed by a spline, and two thresholds one for the average GV and another one for the minimum GV were applied to eliminate remaining unfocused cells.

9. The orientation of the major axis was obtained by fitting an ellipse to the digitized image (Figure 2b). The respective angle corresponds to the principal direction of the inertia tensor of the digitized image. Major and minor axes were obtained from the size of the smallest rectangle enclosing the digitized image. Profiles (Figure 3) were obtained from the GVs of the 16-bit images inside slender rectangles oriented along the principal axes (Figure 2c). The width of the rectangle along the major axis was 0.005 times the minor axis and accordingly for the minor axis.

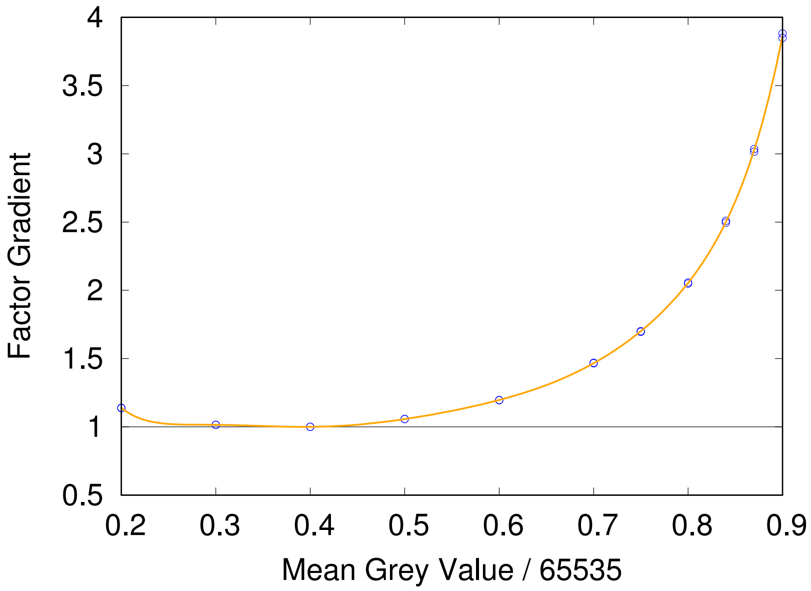

Figure S4: Renormalization of GV-gradients. Abscissa: the relative mean GV of an RBC. Ordinate: the factor to renormalize GV-gradients of RBCs with relative mean GVs > 0.4.

5 Identification of stomatocytes

In order to find criteria to filter stomatocytes, a series of extra experiments was made. Washed RBCs obtained by finger prick were suspended at extremely low concentration in 26% OptiPrep and 74% PBS to avoid sedimentation and supplemented with 0.1 g% bovine albumin in order to achieve moderate stomatocytes despite the glass effect. The suspension was filled in a cone-plate chamber in which both the cone and the plate could be moved in x and y (with the optical axis being along z). With this system, it was possible to rotate cells about any axis lying in the x,y-plane and at the same time keeping it inside the camera image. The optics comprised an objective NPl 100/1.3, a condenser of 0.9 aperture and bright-field illumination by an LED (416 nm). Most RBCs were stomatocytic. Some cells were discocytes on the verge of becoming stomatocytes.

Selected cells were rotated and filmed. From these films, one or two edge-on orientations and one face-on orientation were selected. The face-on orientations were processed as described above. Figures S5, S6, S7, S8 show the results.

The two graphs in line 1 of each of these figures show profiles of GV along the major and minor principal axes as in Figure 3. The two directions are referred to as long and wide in the following. As distinguished from Figure 3, only the central part is shown. Line 2 shows the second derivatives of the red curves in line 1. Line 3 shows microscopic images in the face-on and edge-on orientation.

The vertical black lines in line 2 indicate the locations of the two minima in the graphs of line 1 and the maximum in between. The value of the second derivative at these three locations can be interpreted as the curvature of the profile. Comparing Figures S5 and S6 with Figures S7 and S8 shows that (i) the curvature at the maximum is much lower in stomatocytes than in discocytes and (ii) the curvatures at the minima is much higher. Of the two values at the maximum obtained in long and wide the smaller absolute value was selected. Of the four values at the minima obtained in long and wide the largest two were selected. A combination of both values was used as a criterion to filter stomatocytes.

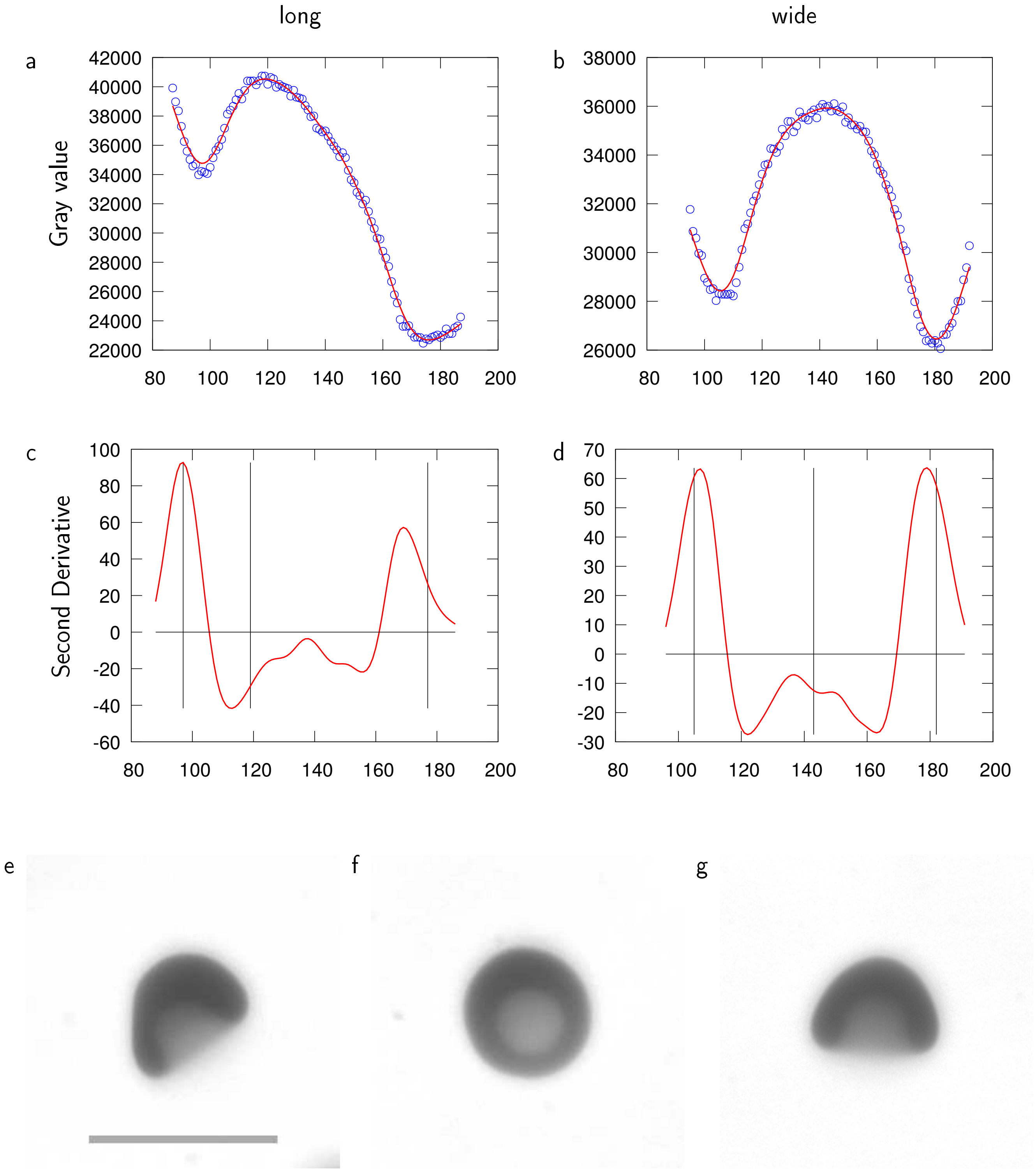

Figure S5: Data of a severely asymmetric stomatocyte; line 1: profiles as in Figure 3, however, only the central part is shown (a: along the major axis, b: along the minor axis); line 2: Second derivative of the respective red curves in line 1; e: view edge-on corresponding approximately to the section in a, the scale bar indicates 10 µm; f: view face-on; g: view edge-on corresponding approximately to the section in b.

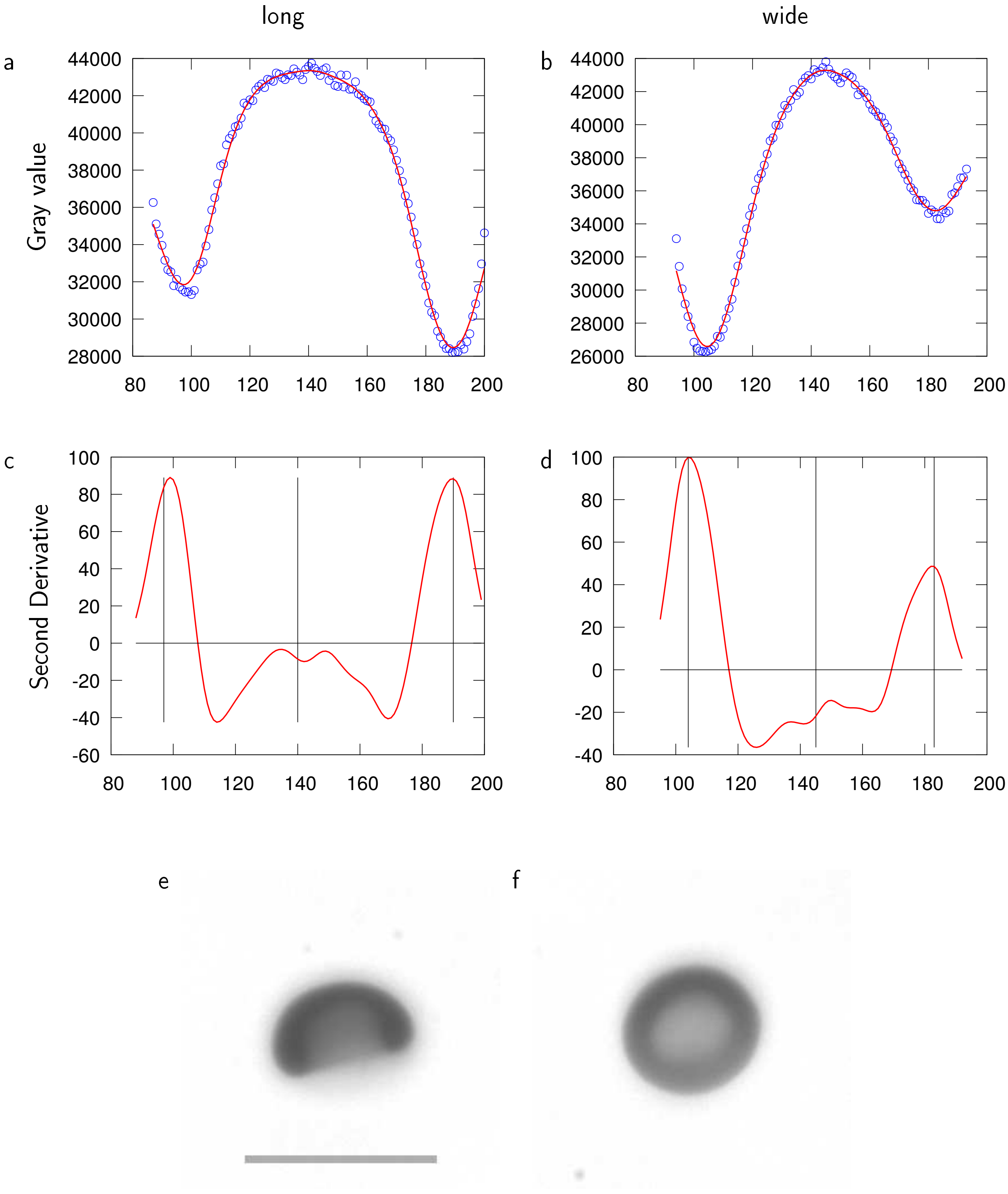

Figure S6: Data of a moderately asymmetric stomatocyte; line 1: profiles as in Figure 3, however, only the central part is shown (a: along the major axis, b: along the minor axis); line 2: Second derivative of the respective red curves in line 1; e: view edge-on corresponding approximately to the section in a, the scale bar indicates 10 µm; f: view face-on.

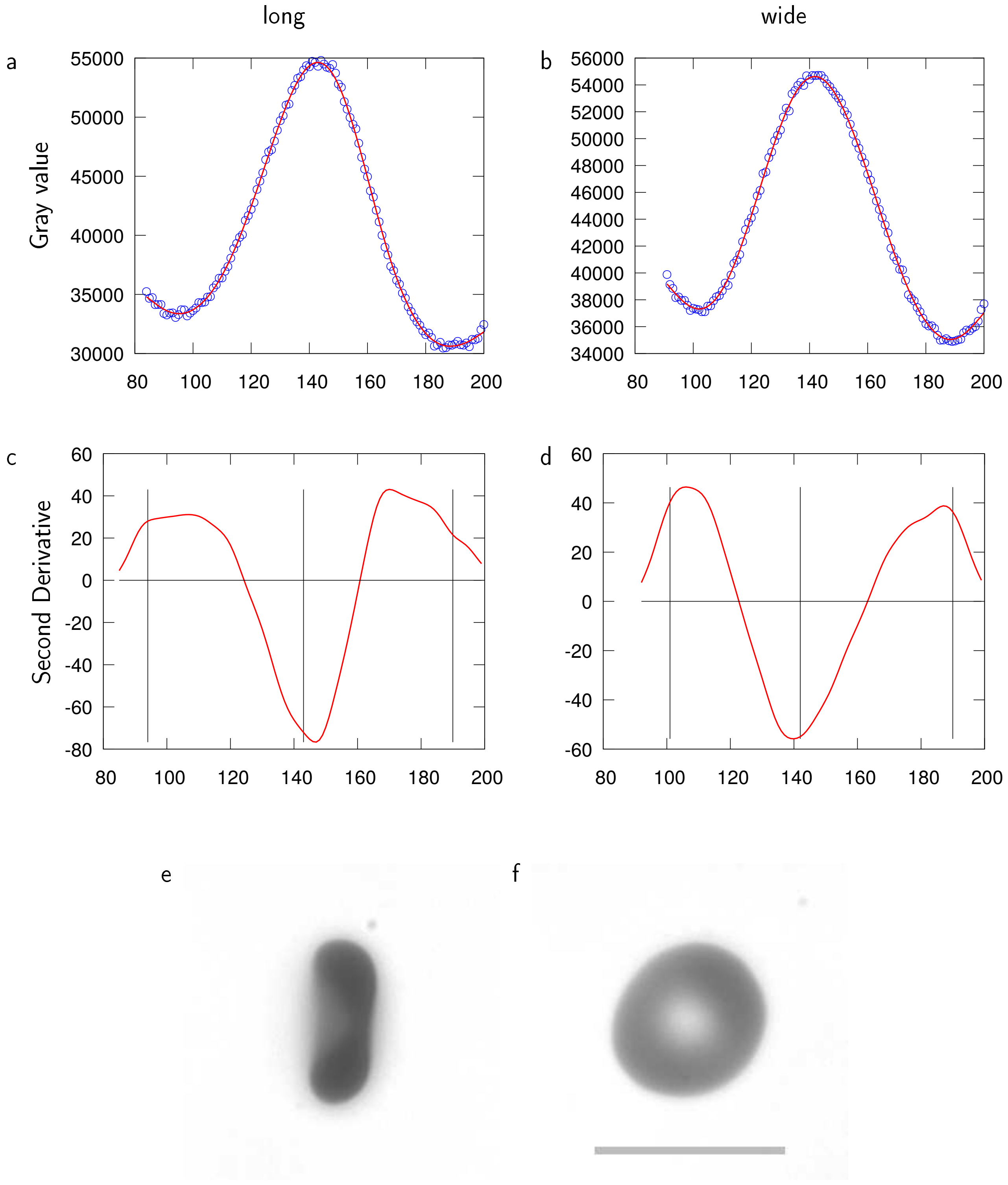

Figure S7: Data of a monoconcave discocyte; line 1: profiles as in Figure 3, however, only the central part is shown (a: along the major axis, b: along the minor axis); line 2: Second derivative of the respective red curves in line 1; e: view edge-on corresponding approximately to the section in a; f: view face-on, the scale bar indicates 10 µm.

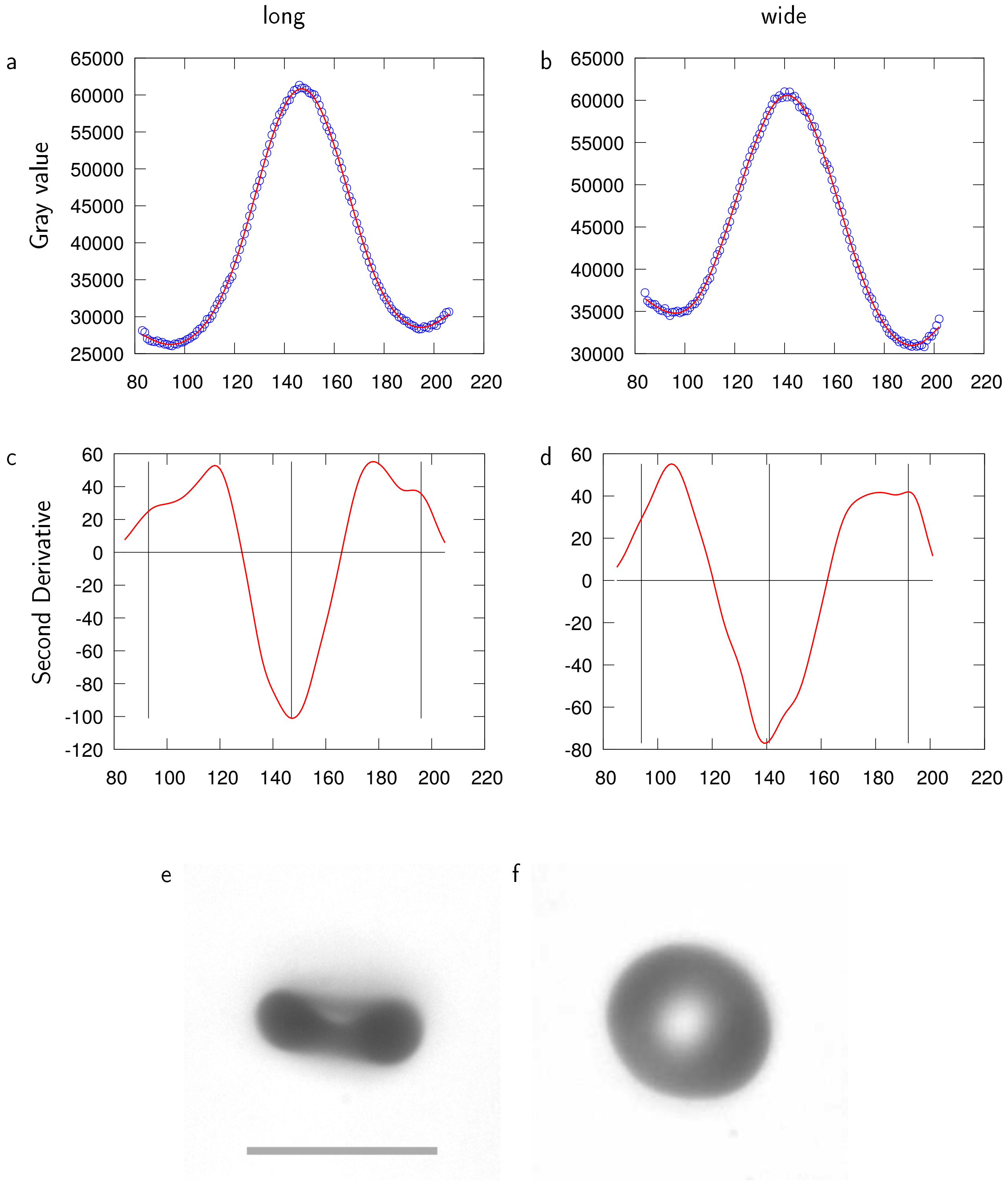

Figure S8: Data of a biconcave discocyte; line 1: profiles as in Figure 3, however, only the central part is shown (a: along the major axis, b: along the minor axis); line 2: Second derivative of the respective red curves in line 1; e: view edge-on corresponding approximately to the section in a, the scale bar indicates 10 µm; f: view face-on.

6 Identification of echinocytes

The method to identify echinocytes was based on the plugin "Contour Curvature" in imagej. Figure S9a shows the original image of an E2 after background removal. Figure S9b shows in black and white the same cell after conversion to a binary object. Figure S9c shows in light blue the contour of the shape in Figure S9b. This contour is smoothed by the plugin using Fourier shape-descriptors with 12 Fourier coefficient-pairs (red in Figure S9c). In order to distinguish the two colors it necessary to zoom in. The curvature of the smoothed outline and its derivative are shown in Figure S9e. Echinocytes were filtered using the SD of the curvature and the minimum of the derivative.

Echinocytes found via this procedure were further differentiated using the plugin "Oval Profile" in imagej. An ellipse was defined inside the contour of the RBC (see Figure S9d). The axes of the ellipse were 0.7 times the major and minor axis of the RBC. The variation of the GV along this line is shown in Figure S9f with blue circles. The red curve is a spline through the data. Figure S9g shows the derivative of the spline. The criterion to filter E2 was a threshold for value of max{max(derivative), -min(derivative)}. In Figure S9g, the value would be ≈ 1.4. Echinocytes not classified as E2 were taken as E1.

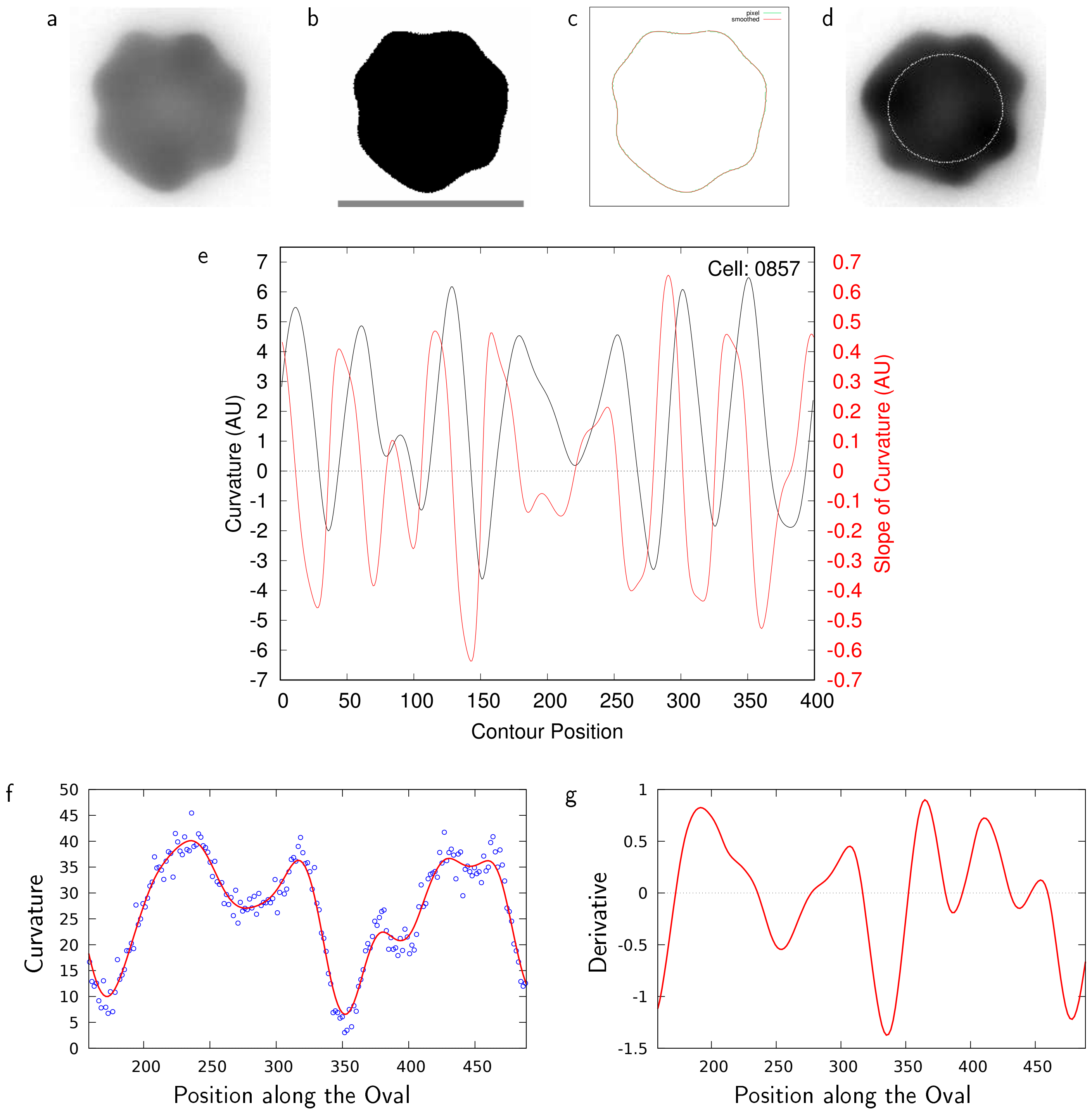

Figure S9: For details see text. b: The scale bar indicates 10 µm. d: Please note that the cell is rotated to make the major axis horizontal.

7 Poikilocytes

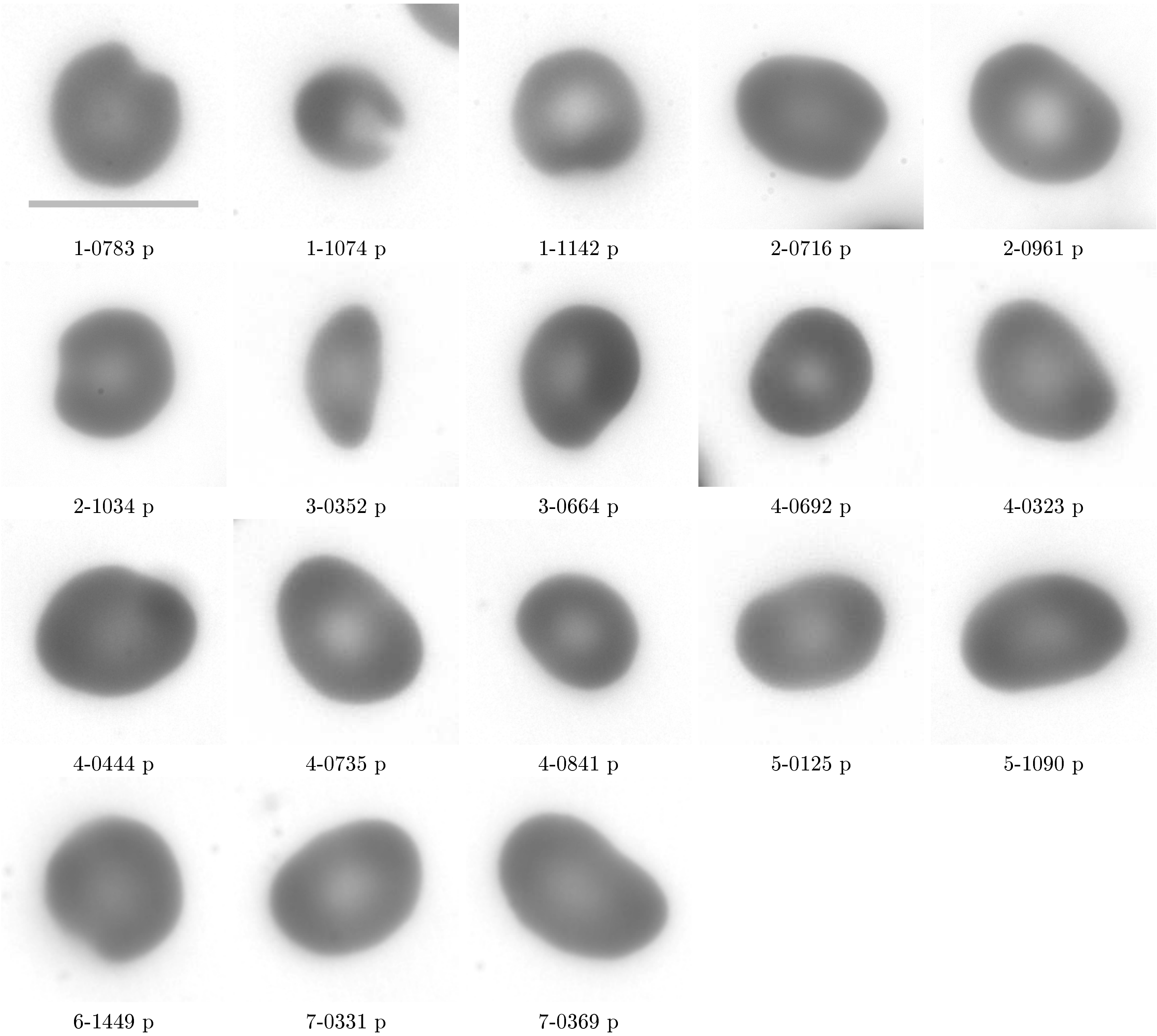

Figure S10: All pitting type 1 cells found in plasma suspensions. The code under each cell means: donor-cellNumber plasma. The scale bar indicates 10 µm.

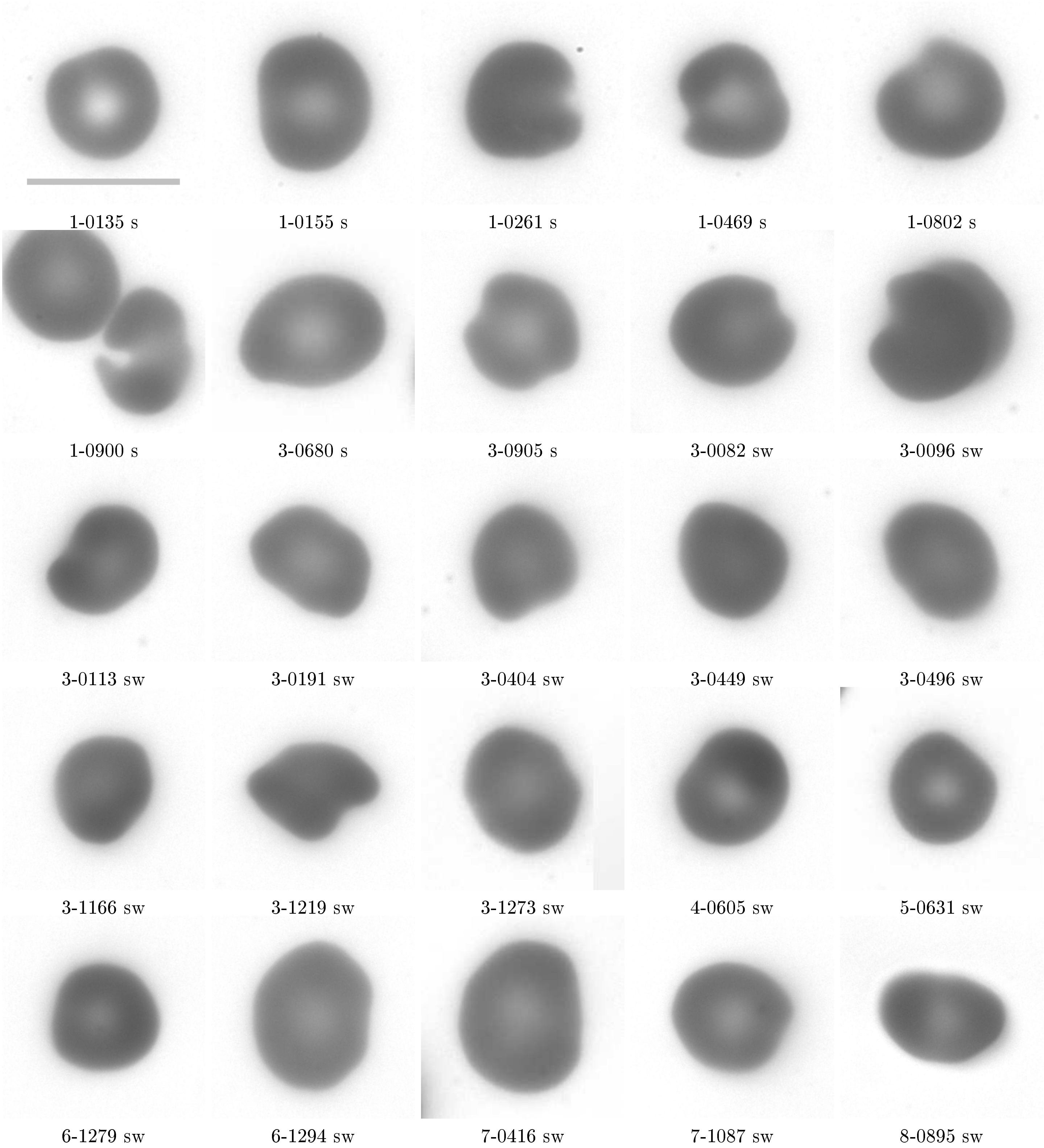

Figure S11: All pitting type 1 cells found in serum suspensions. The code under each cell means: donor-cellNumber serum. The object 3-0094 is an aggregated doublet. The scale bar indicates 10 µm.

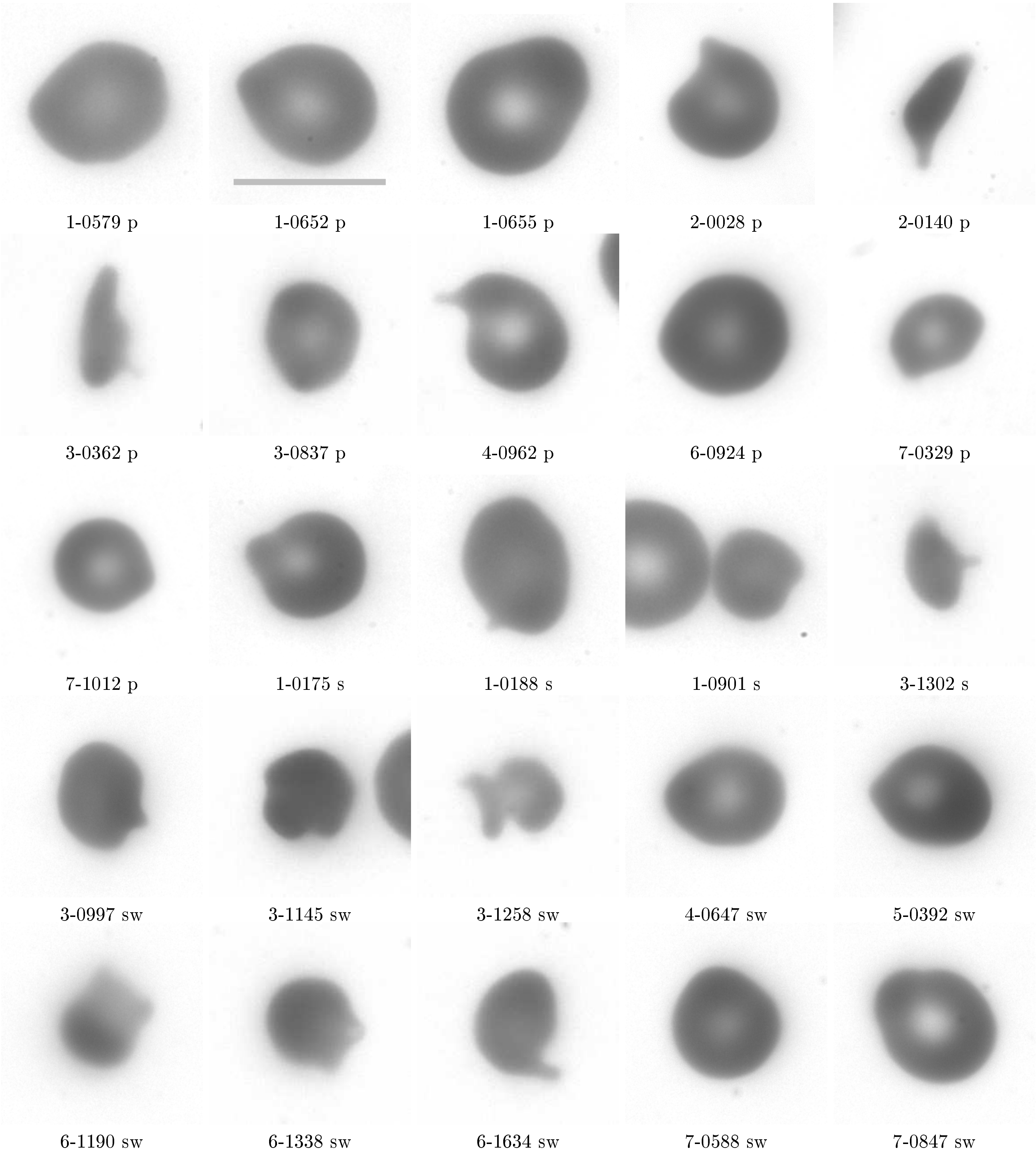

Figure S12: All pitting type 2 cells found in plasma or serum suspensions. The code under each cell means: donor-cellNumber plasma or serum. The scale bar indicates 10 µm.

8 Comparison of the focus along the major and minor axes

In order to check whether an inclined orientation of red RBCs dupes an increased aspect ratio, the focus of RBCs along the major and minor axes was compared. To this end, the list of GVs along the contour obtained during image processing (Section S4, step S8) was used. This list represents the gradients in GV at the border of the contrast enhanced image.

Four selections from this list were used. In cells with an aspect ratio of unity, each of the four selections comprised the same number of data. With increasing aspect ratio, this number decreased along the major axis and increased along the minor axis in a linear fashion. An example is shown in Figure S13.

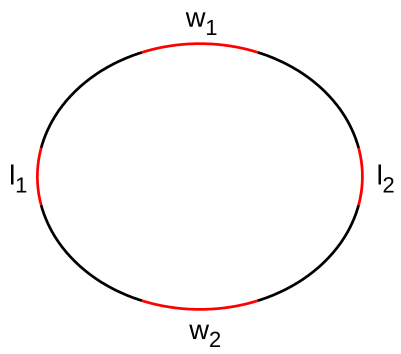

Figure S13: The red parts of the circumference indicate the position and size of the four selections.

The values in each of the four selections were averaged and denoted as shown in Figure S13. The ratio $\frac{|w_1 - w_2|}{|l_1 - l_2|}$ was plotted versus aspect ratio. An example (of donor 4) is shown in Figure S14. The plots for all donors are shown in the data-sheets (Supplementary Materials, Document 2). All correlation coefficients including the values for plasma and serum suspension range from -0.065 to 0.059.

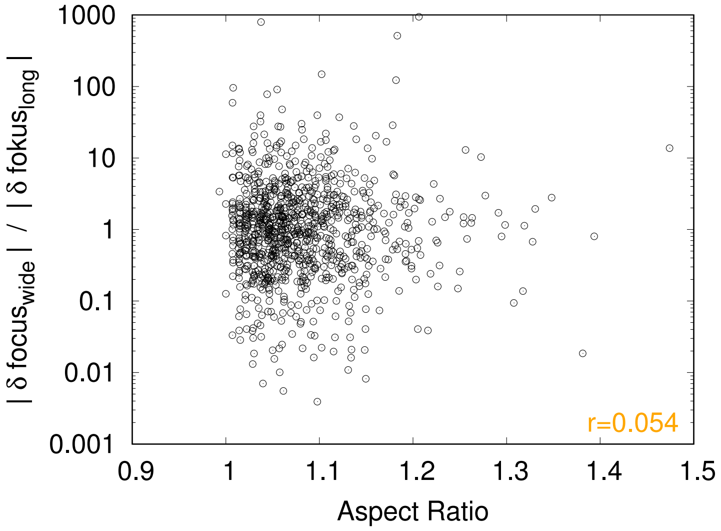

Figure S14: Correlation of $\frac{|w_1 - w_2|}{|l_1 - l_2|}$ with aspect ratio. The orange number denotes the correlation coefficient.

9 Cumulated data

9.1 Distributions of medians and means, plasma

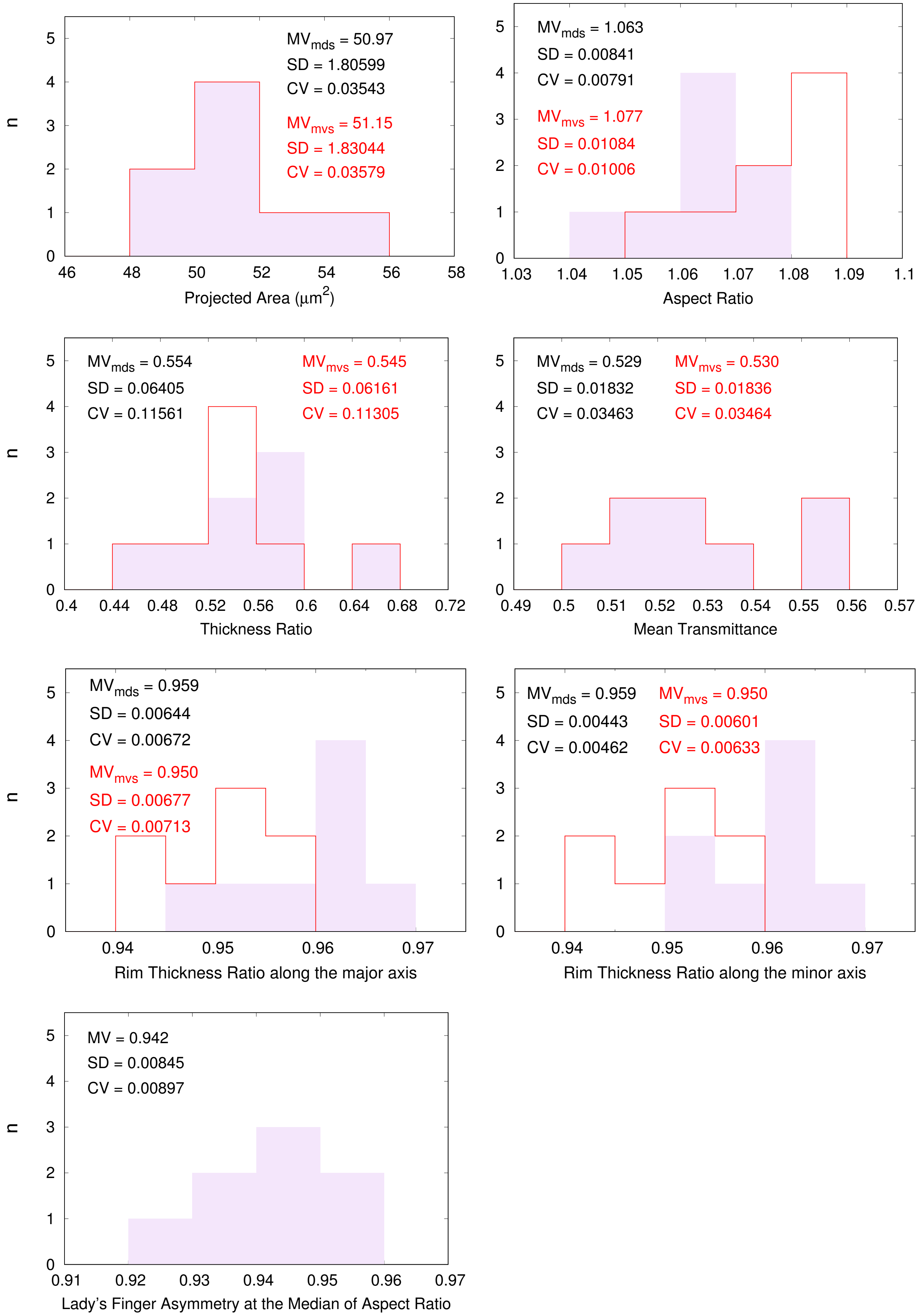

Figure S15: Distributions of the medians (gray) and means (red) of seven parameters of eight donors. Suspension of the RBCs in plasma. SDs correspond to standard errors of the means and medians, respectively.

9.2 Distributions of medians and means, serum

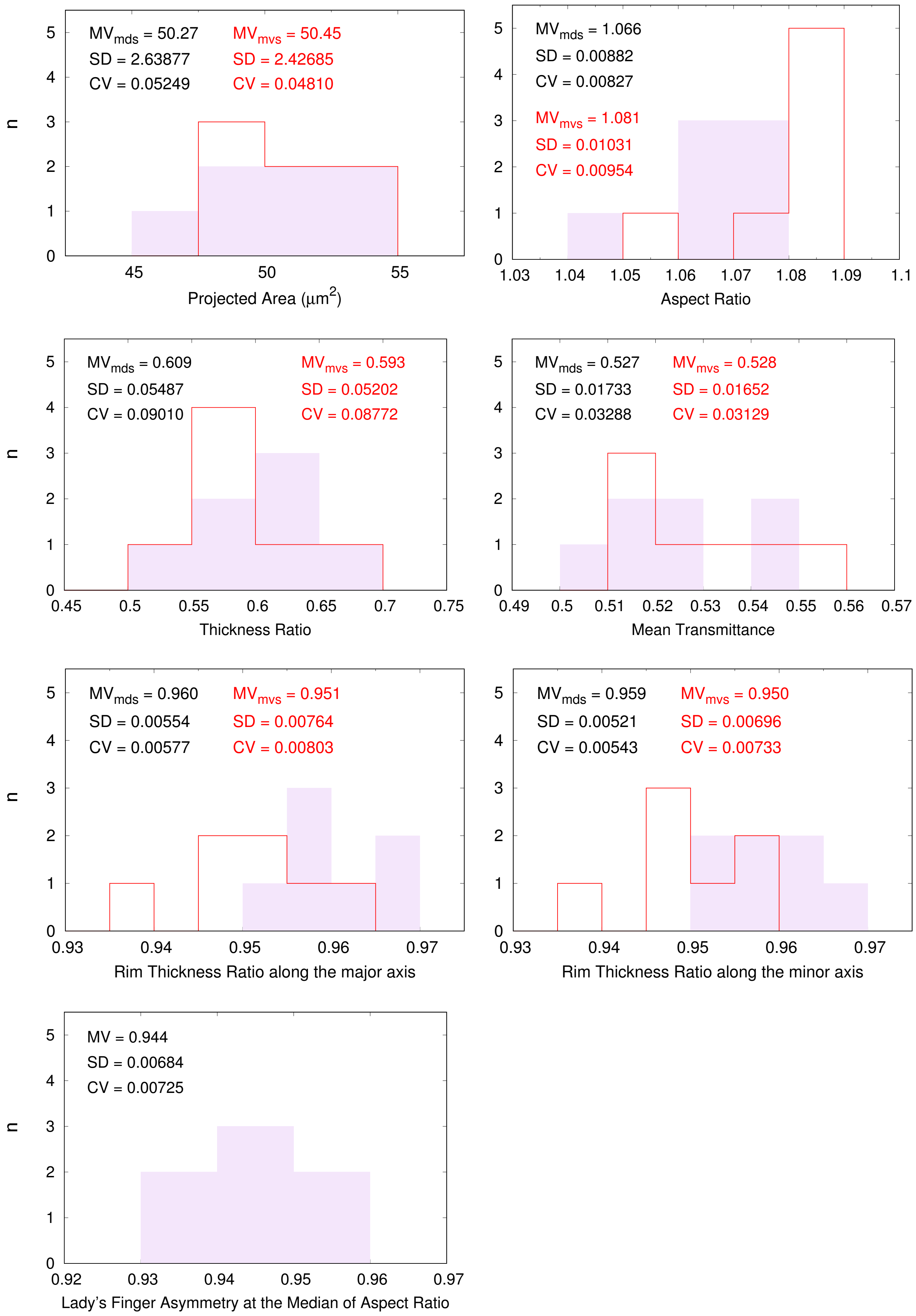

Figure S16: Distributions of the medians (gray) and means (red) of seven parameters of seven donors. Suspension of the RBCs in serum. SDs correspond to standard errors of the means and medians, respectively.

9.3 Correlation coefficients of the scatter plots

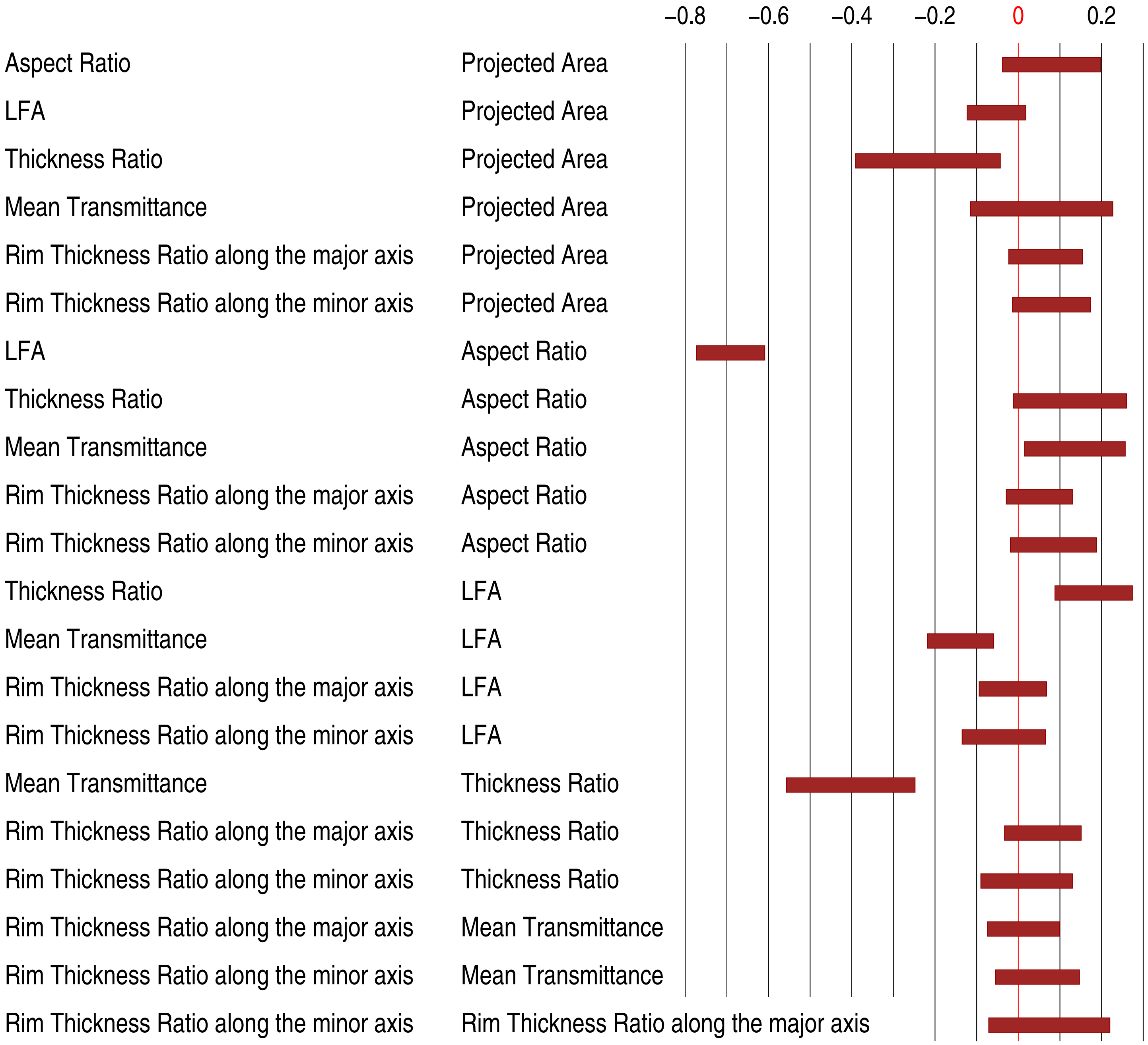

Figure S17: Scatter plots show the dependence of a pair of two quantities. Seven quantities result in 21 scatter plots. The plots are shown in each donor-specific data-sheet (Supplementary Materials, Document 2). Here the ranges of the correlations coefficients including the values for plasma and serum suspension are shown. Because of limited space the labels "Rim Thickness Ratio along the major axis" or "Rim Thickness Ratio along the minor axis" are used instead of: ratios between rim thicknesses at the two opposite ends of the major and minor axis, respectively.

9.4 Comparison between plasma and serum

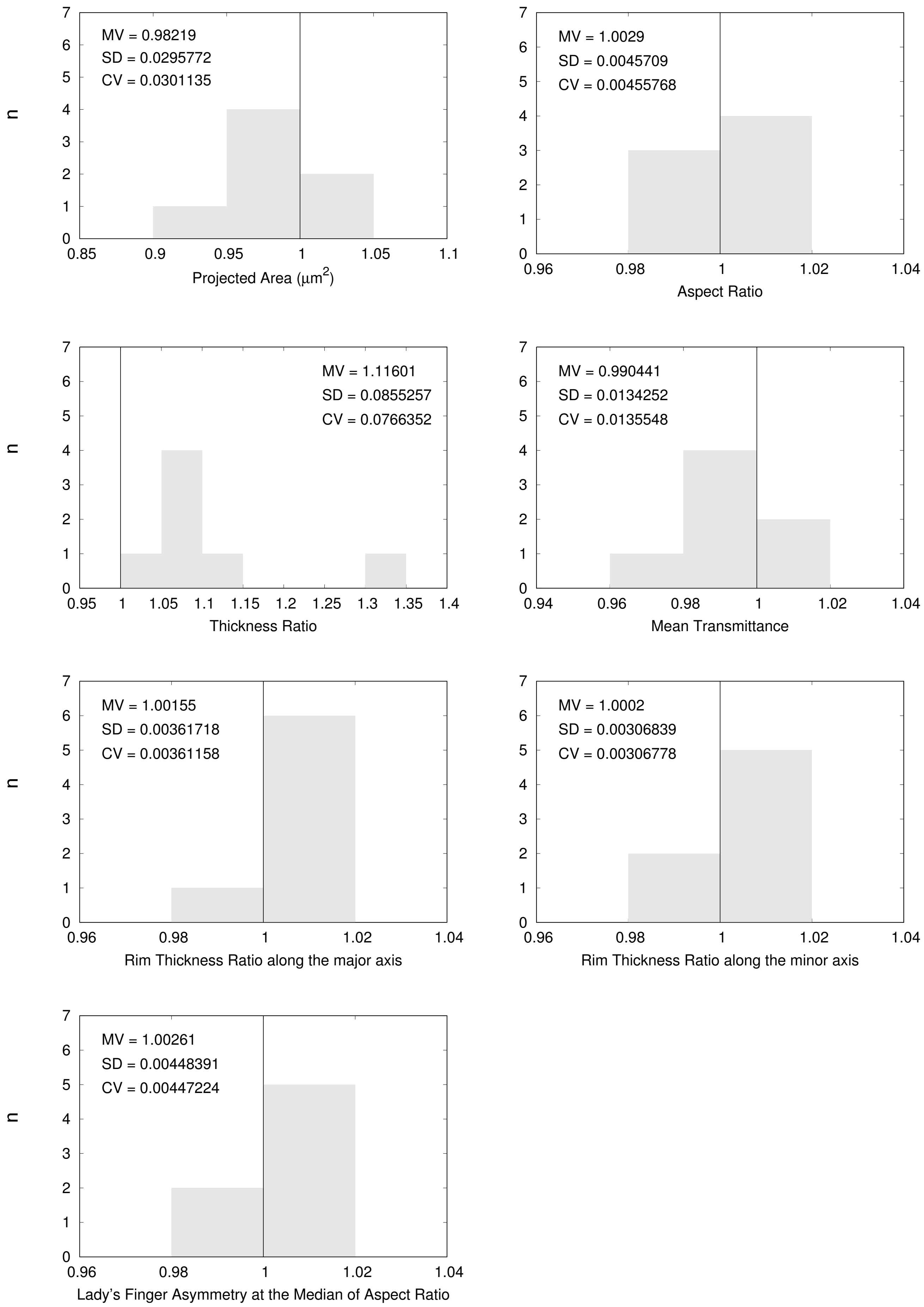

Figure S18: Distributions of the ratio of values obtained for serum suspension and plasma suspension. Plot 1–6: the medians of the respective distributions. Plot 7: <LFA>.

10 Thermal effects

10.1 Lateral diffusion

After collection of the images of donor 4, a movie was taken at low light level with 1 frame/s with the same preparation. A movie (Supplementary Materials, Movie 2) shows a clipping with three cells. The movie is speeded up 25 times. Figure S19 shows the random walks of these three cells during 14.8 min. The rotational diffusion is shown in another experiment lasting 5.1 s (Figure 11a). The results show that RBCs suspended in plasma do not adhere to glass.

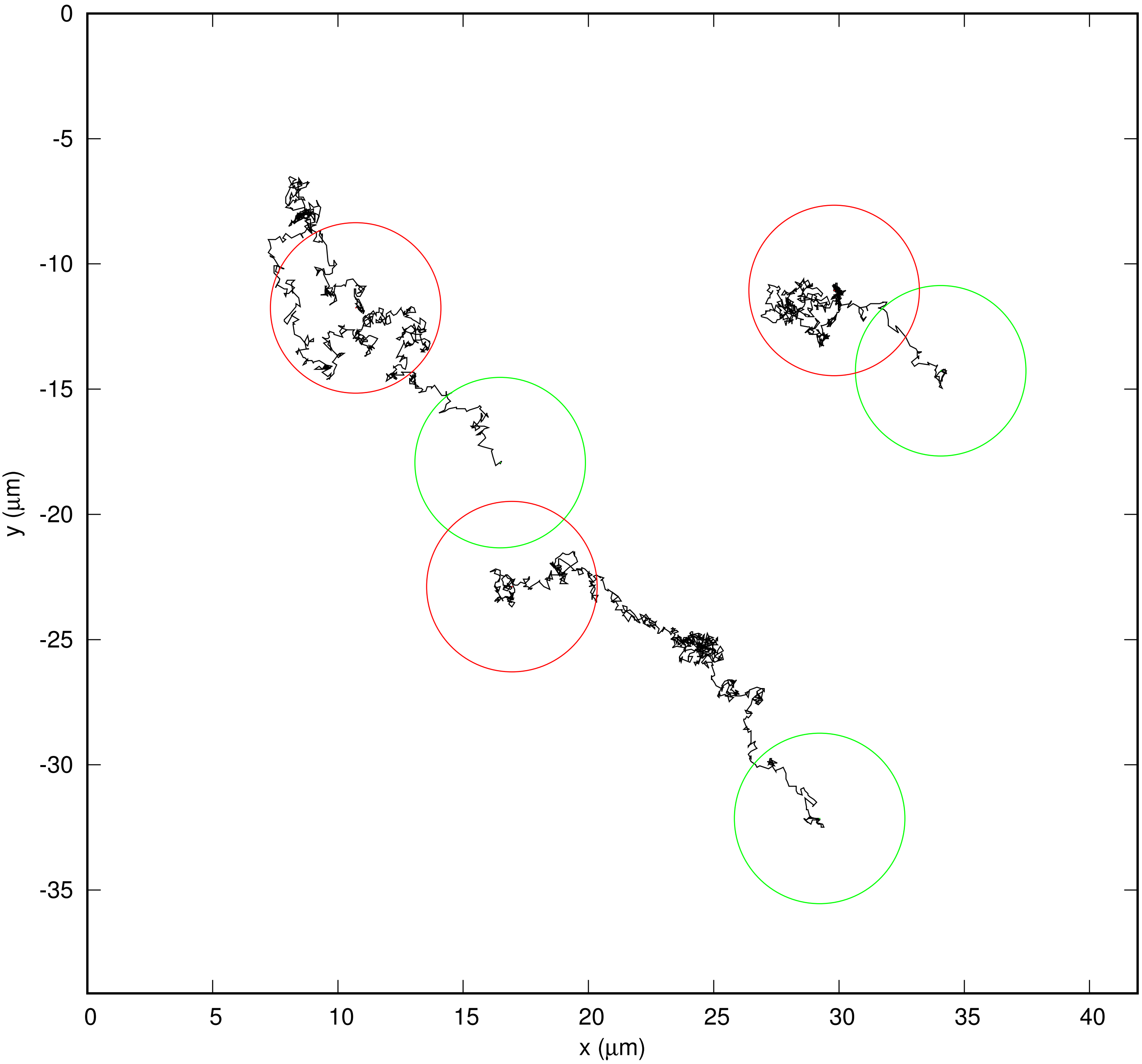

Figure S19: Random walks of three RBCs in plasma lasting 14.8 min. The beginning of each random walk is indicated by a green circle, the end by a red circle. The size of the circles corresponds approximately to the size of the RBCs.

10.2 Camera noise and flickering

A suspension of RBCs in plasma was filmed with 400 frames/s for about 5 s. A clipping of a single RBC was evaluated in the same way as all red cells in this study. Figure S20 shows the time dependence of the GV of the four rim positions and the two dimple values.

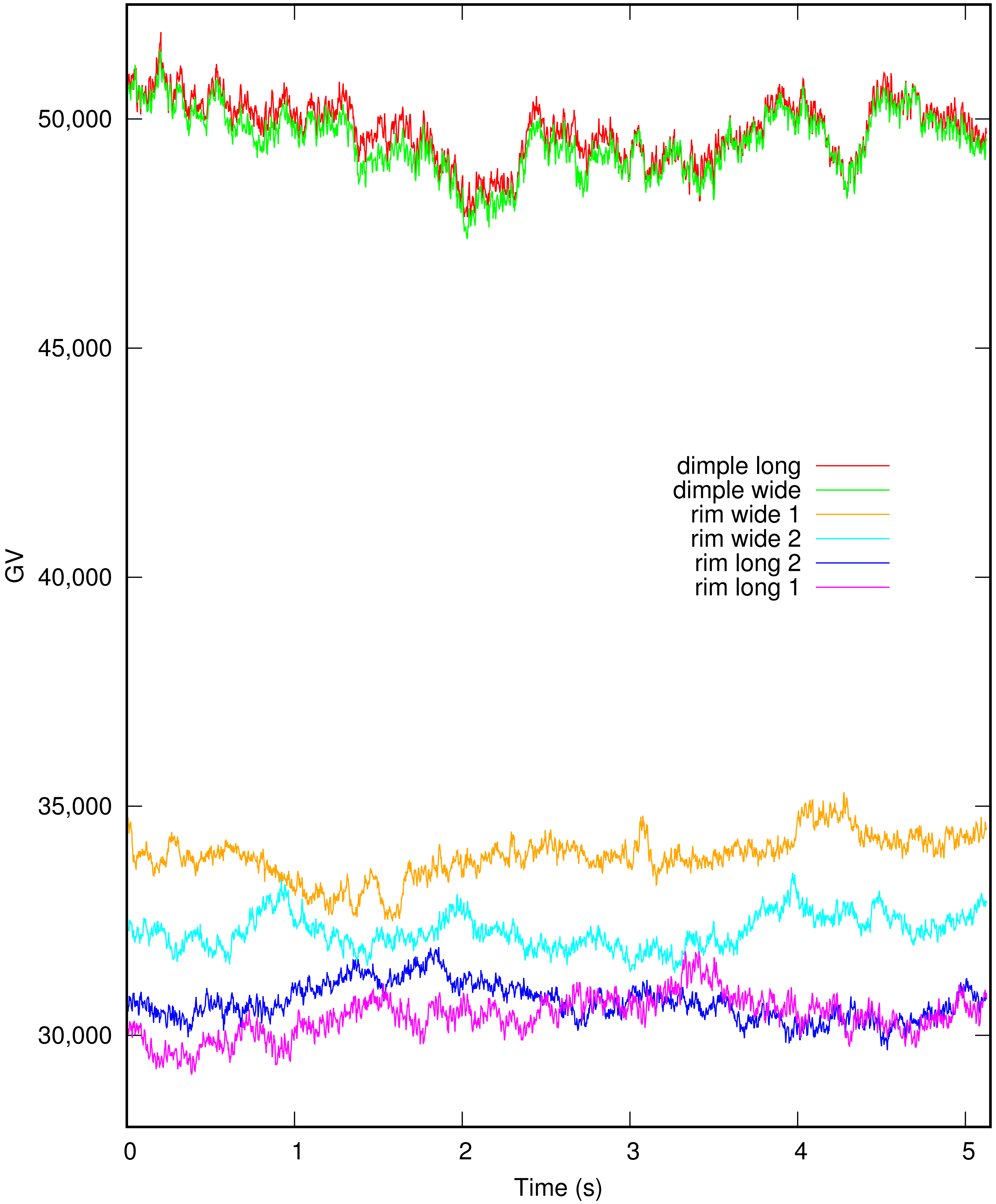

Figure S20: Time series of GVs taken from a movie taken with 400 frames/s.

11 Effects of gravity

Distortions due to gravity of the shape of phospholipid vesicles resting on a solid surface have been studied theoretically [3]. In the absence of adhesion, a critical number g $\simeq 0.45$ was provided separating two regimes (i) g < 0.45 resulting in a point contact, i.e. no distortion and (ii) contact of an area > 0 due to distortion.

$$\mathrm{g} = \frac{g_0 \ \Delta\rho \ R_0^4}{\kappa}, \tag{6}$$

with the gravitational constant $g_0 = 9.81\,\mathrm{m/s^2}$, the density difference between cytoplasm of the RBC and plasma $\Delta\rho = 0.08\,\mathrm{kg}/\ell$ [4], the radius of the vesicle, here representative for the RBC chosen as $\mathrm{R}_0 = 3\,\mu\mathrm{m}$, and the bending stiffness of the vesicle, here for the RBC chosen as $\kappa = 2 \cdot 10^{-19}\,\mathrm{J}$.

Inserting the numbers results in g=0.3. This is a conservative estimate since the RBC membrane is much stiffer than a vesicle membrane due to its membrane skeleton. The estimate shows that deformations due to gravity are virtually absent.

12 Design of an average RBC

I admit that I do not have experience with *in silico* modeling. Therefore, some of the following suggestions may be naive.

surface area: A value can be adopted from the average of two donors obtained by Gifford et al. [5]. The value pertaining for RBCs in the middle of their mature life was found as 147.3 $\mu\mathrm{m}^2$.

starting conditions: A spherical triangulated network with bending stiffness [6, 7]. The spontaneous curvature may be zero. Its value is adjusted in a later step. If necessary for numerical stability, some shear stiffness (relaxed in the spherical shape) may be added.

volume: Upon reduction of the volume, the surface will adopt a biconcave shape [8]. The final reduced volume may be the accepted value of 0.65.

THR: Its value can be adjusted to 0.55 by changing the spontaneous curvature. An increase of the spontaneous curvature increases the THR and vice versa. For later use, the axis of symmetry is taken as the z-axis of a orthogonal coordinate system.

LFA: This is the most difficult step. The triangles at the maximum rim thickness should be displaced in a sinusoidal fashion to obey the value LFA=0.942. A plot of the tangential GVs of the rim (not shown) gave no evidence for a more specific function than a sine.

aspect ratio: All vertex points of the triangulated network should be moved proportional in x and y to achieve an aspect ratio of 1.07.

tuning: If the final surface area differs from the starting value, go back to step "surface area" and increase or decrease the value appropriately. Alternatively, the values of the major and minor axes could be made to agree with the values 8.38 µm and 7.83 µm found here for n=8.

References

[1] Bessis, M., 1972. Red Cell Shapes. An Illustrated Classification and its Rationale. *Nouvelle Revue Française d'Hématologie* 12:721–746.

[2] Schneider, C. A., W. S. Rasband, and K. W. Eliceiri, 2012. NIH Image to ImageJ: 25 years of image analysis. *Nature Methods* 9:671–675.

[3] Kraus, M., U. Seifert, and R. Lipowsky, 1995. Gravity-Induced Shape Transformations of Vesicles. *Europhysics Letters (EPL)* 32:431–436.

[4] Mohandas, N., A. Johnson, J. Wyatt, L. Croisille, J. Reeves, D. Tycko, and W. Groner, 1989. Automated quantitation of cell density distribution and hyperdense cell fraction in RBC disorders. *Blood* 74:442–447.

[5] Gifford, S. C., J. Derganc, S. S. Shevkoplyas, T. Yoshida, and M. W. Bitensky, 2006. A detailed study of time-dependent changes in human red blood cells: from reticulocyte maturation to erythrocyte senescence. *British Journal of Haematology* 135:395–404.

[6] Helfrich, W., 1973. Elastic properties of lipid bilayers: theory and possible experiments. *Z. Naturforsch., C: Biosci.* 28:693–703.

[7] Deuling, H. J., and W. Helfrich, 1976. Red Blood Cell Shapes as Explained on the Basis of Curvature Elasticity. *Biophys. J.* 16:861–868.

[8] Canham, P. B., 1970. The Minimum Energy of Bending as a Possible Explanation of the Biconcave Shape of the Human Red Blood Cell. *J. Theoret. Biol.* 26:61–81.
