# Supplementary material for: The Shape of Human Red Blood Cells Suspended in Autologous Plasma and Serum
Source: Cells. 2022 Jun 16;11(12):1941. doi: 10.3390/cells11121941 (PMC9222013; doi:10.3390/cells11121941)
Supplement: Supplementary file 1 [file cells-11-01941-s001.zip › cells-1742284-supplementary/Documents S2-S6/cells-1742284 - Suppl. Document S2.pdf]

Donor 1, plasma

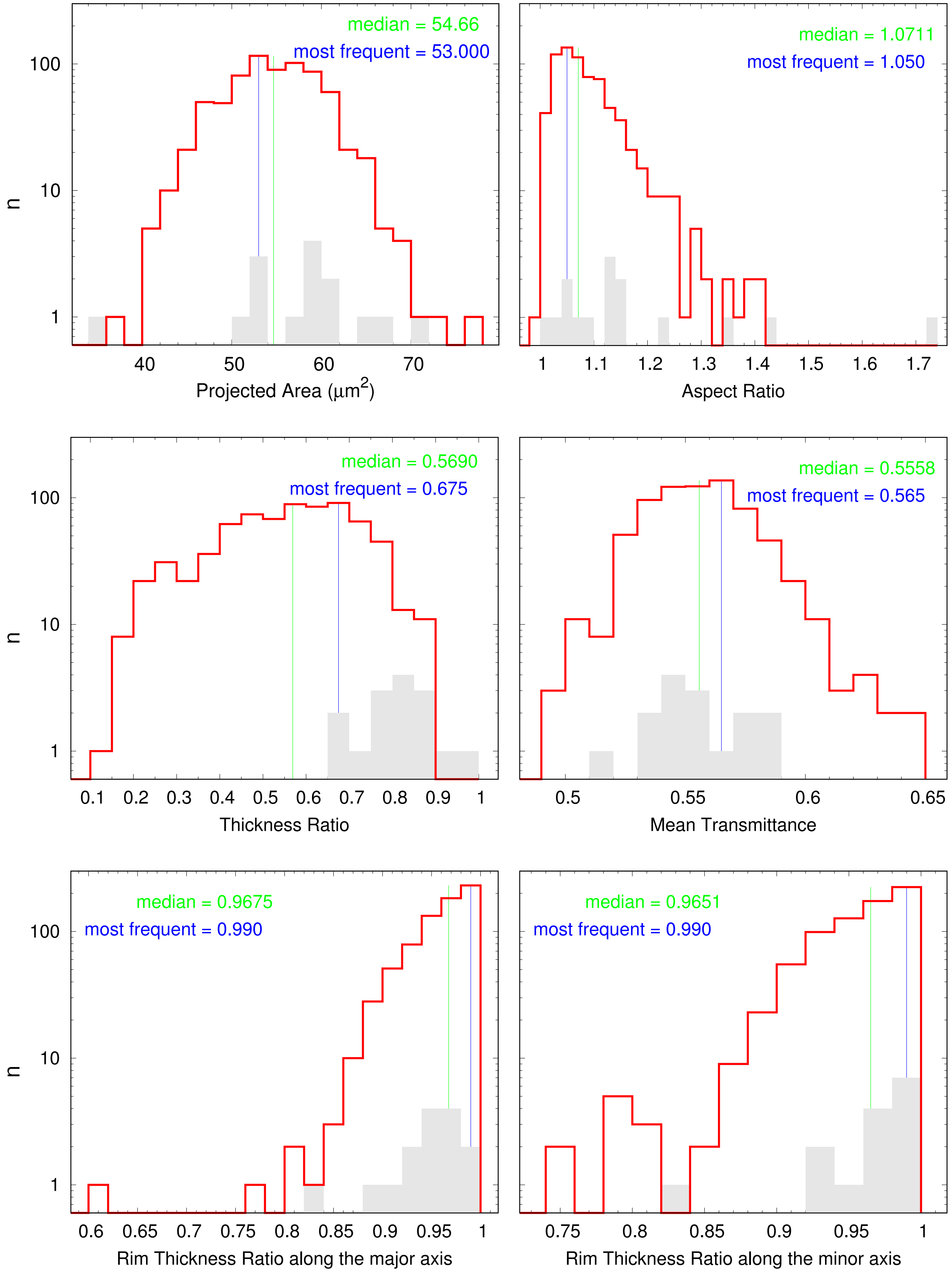

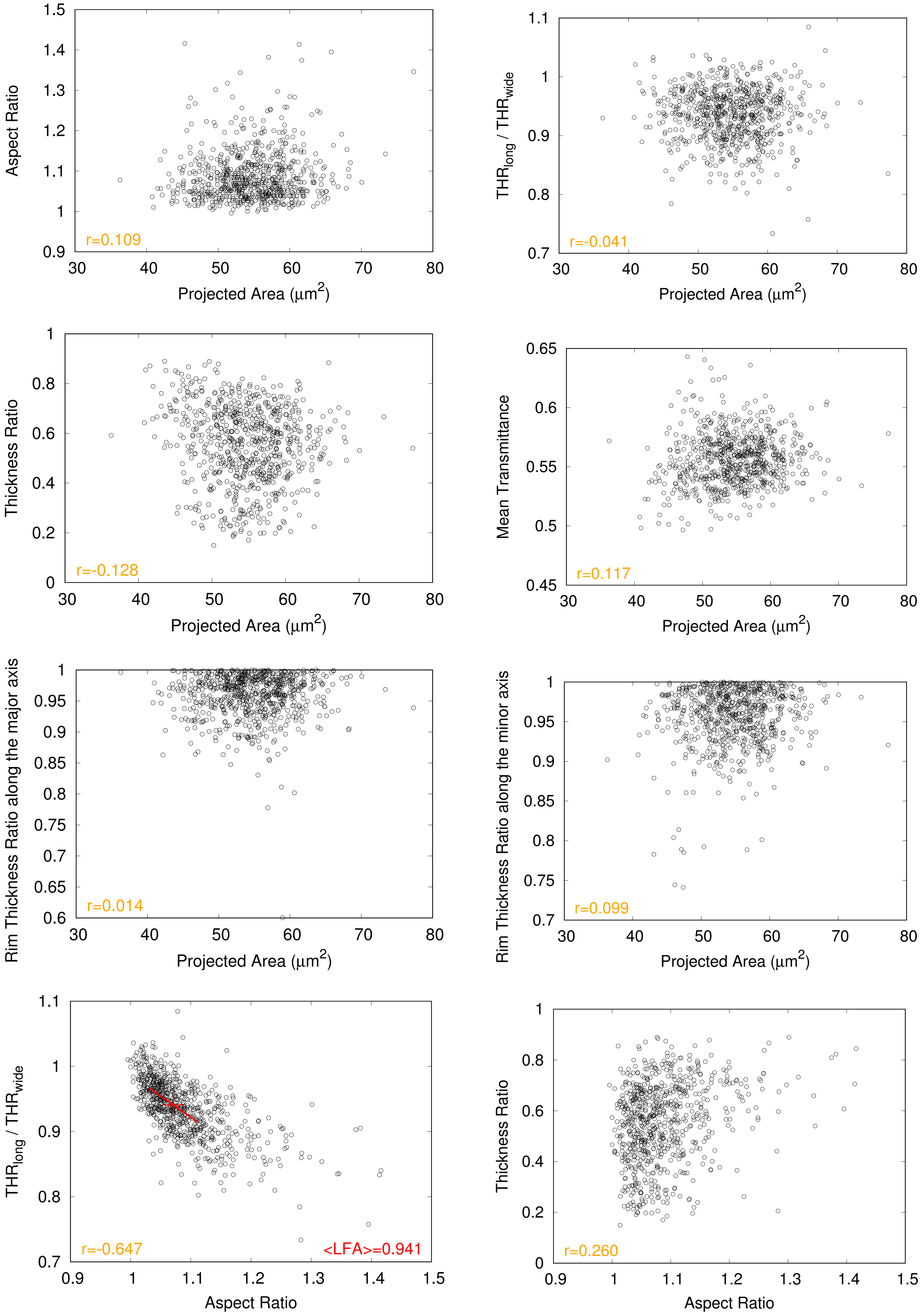

Aspect Ratio
Projected Area (μm²)
r=0.109
THR_long / THR_wide
Projected Area (μm²)
r=-0.041
Thickness Ratio
Projected Area (μm²)
r=-0.128
Mean Transmittance
Projected Area (μm²)
r=0.117
Rim Thickness Ratio along the major axis
Projected Area (μm²)
r=0.014
Rim Thickness Ratio along the minor axis
Projected Area (μm²)
r=0.099
THR_long / THR_wide
Aspect Ratio
r=-0.647
<LFA>=0.941
Thickness Ratio
Aspect Ratio
r=0.260

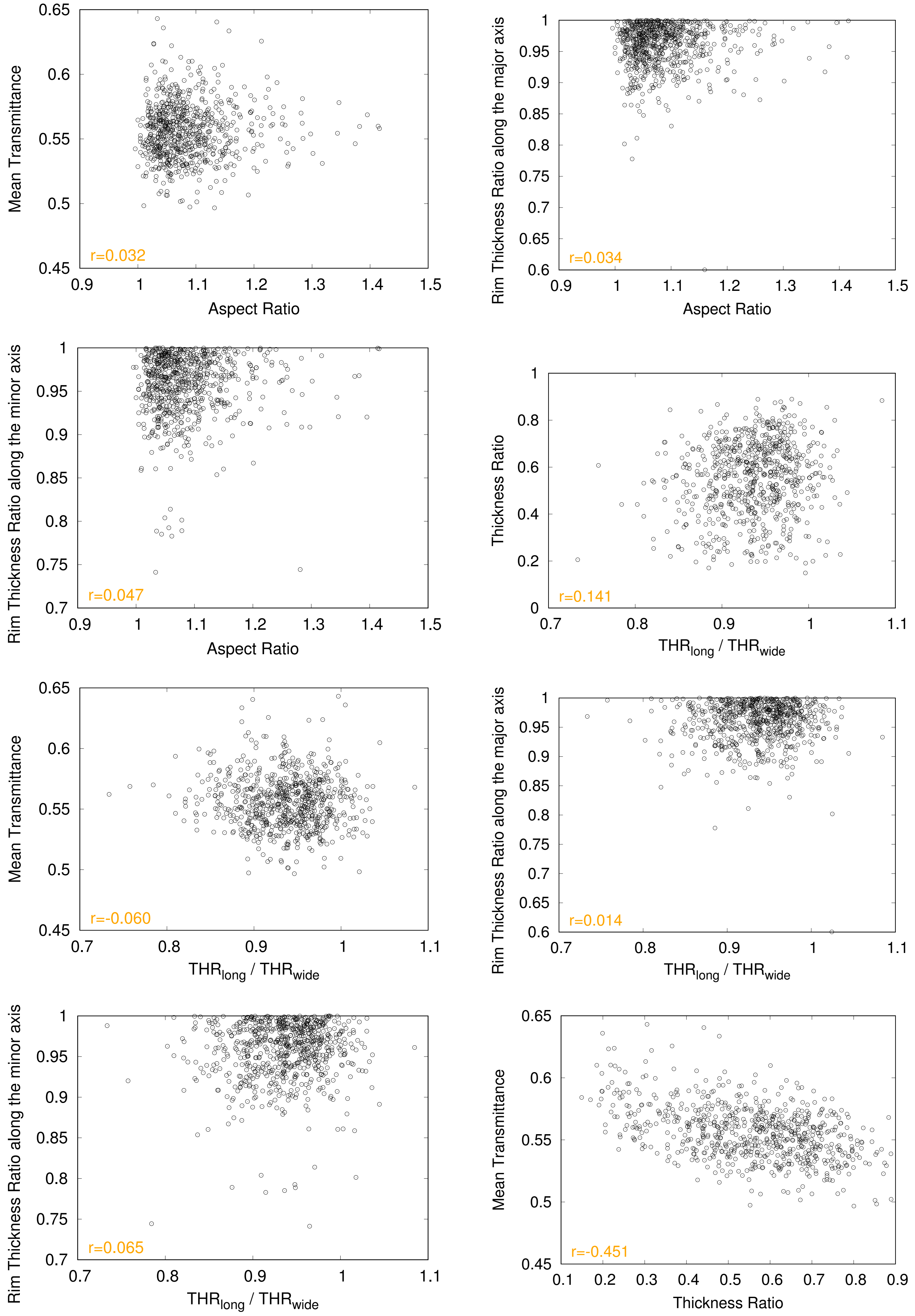
Mean Transmittance
Aspect Ratio
r=0.032
Rim Thickness Ratio along the major axis
Aspect Ratio
r=0.034
Rim Thickness Ratio along the minor axis
Aspect Ratio
r=0.047
Thickness Ratio
THR_{long} / THR_{wide}
r=0.141
Mean Transmittance
THR_{long} / THR_{wide}
r=-0.060
Rim Thickness Ratio along the major axis
THR_{long} / THR_{wide}
r=0.014
Rim Thickness Ratio along the minor axis
THR_{long} / THR_{wide}
r=0.065
Mean Transmittance
Thickness Ratio
r=-0.451

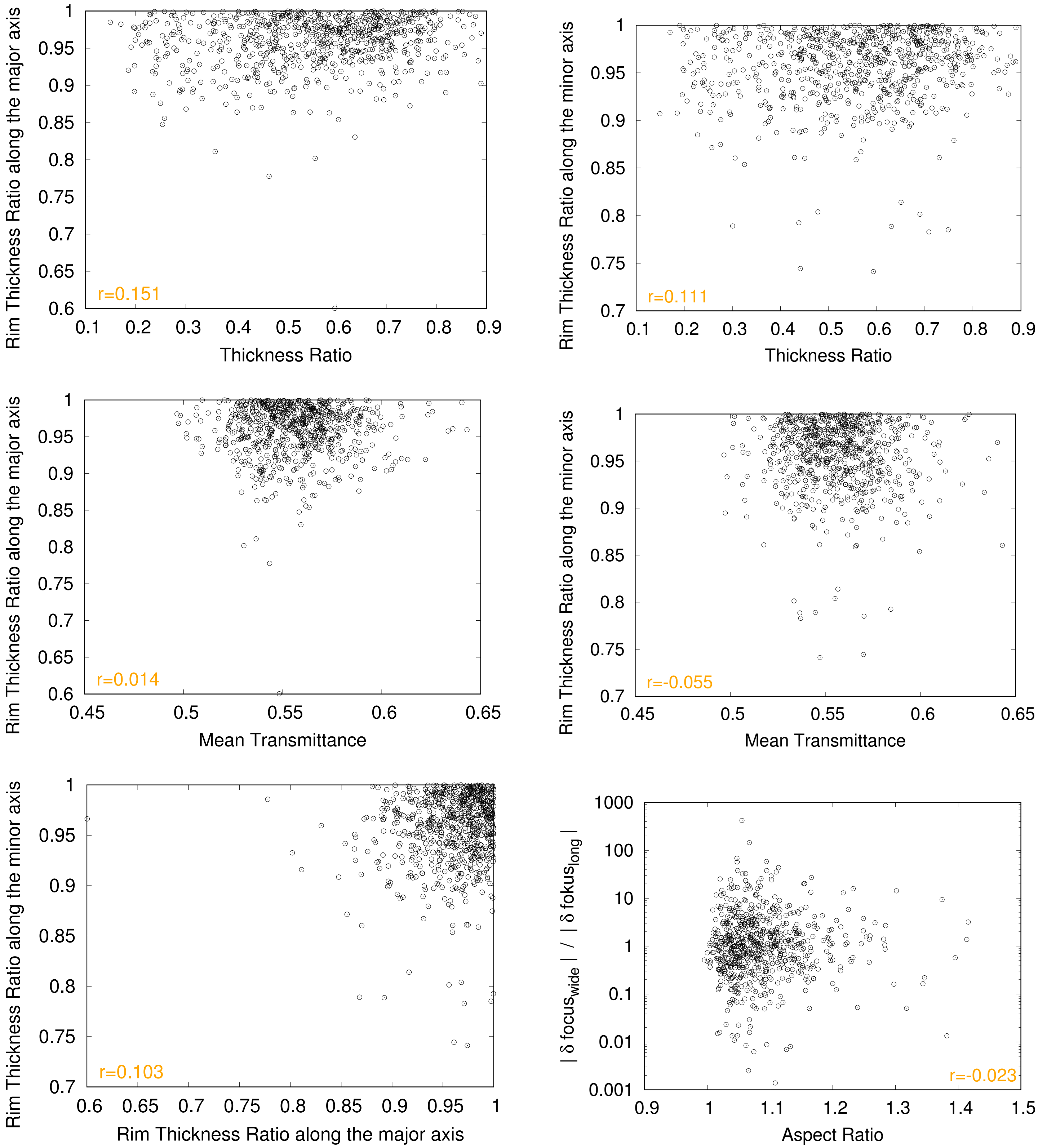

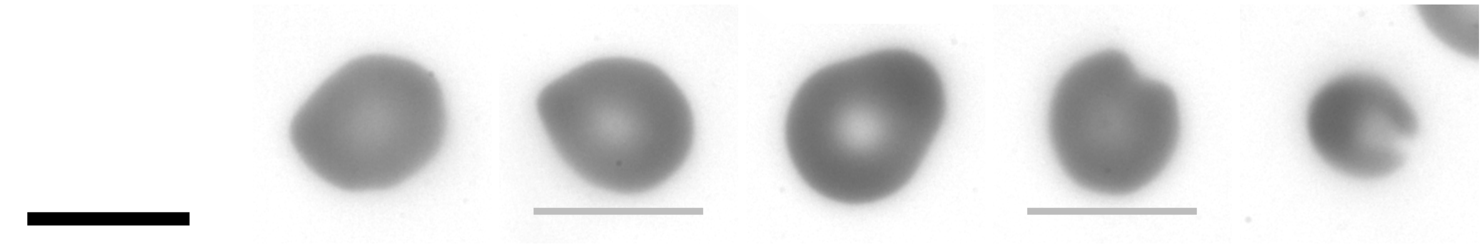

10 µm 0579 0.9005 0652 ob 0655 0.6943 0783 64.7 1074 398

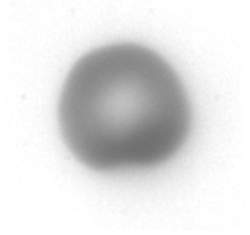

1142 oB

Donor 1, serum

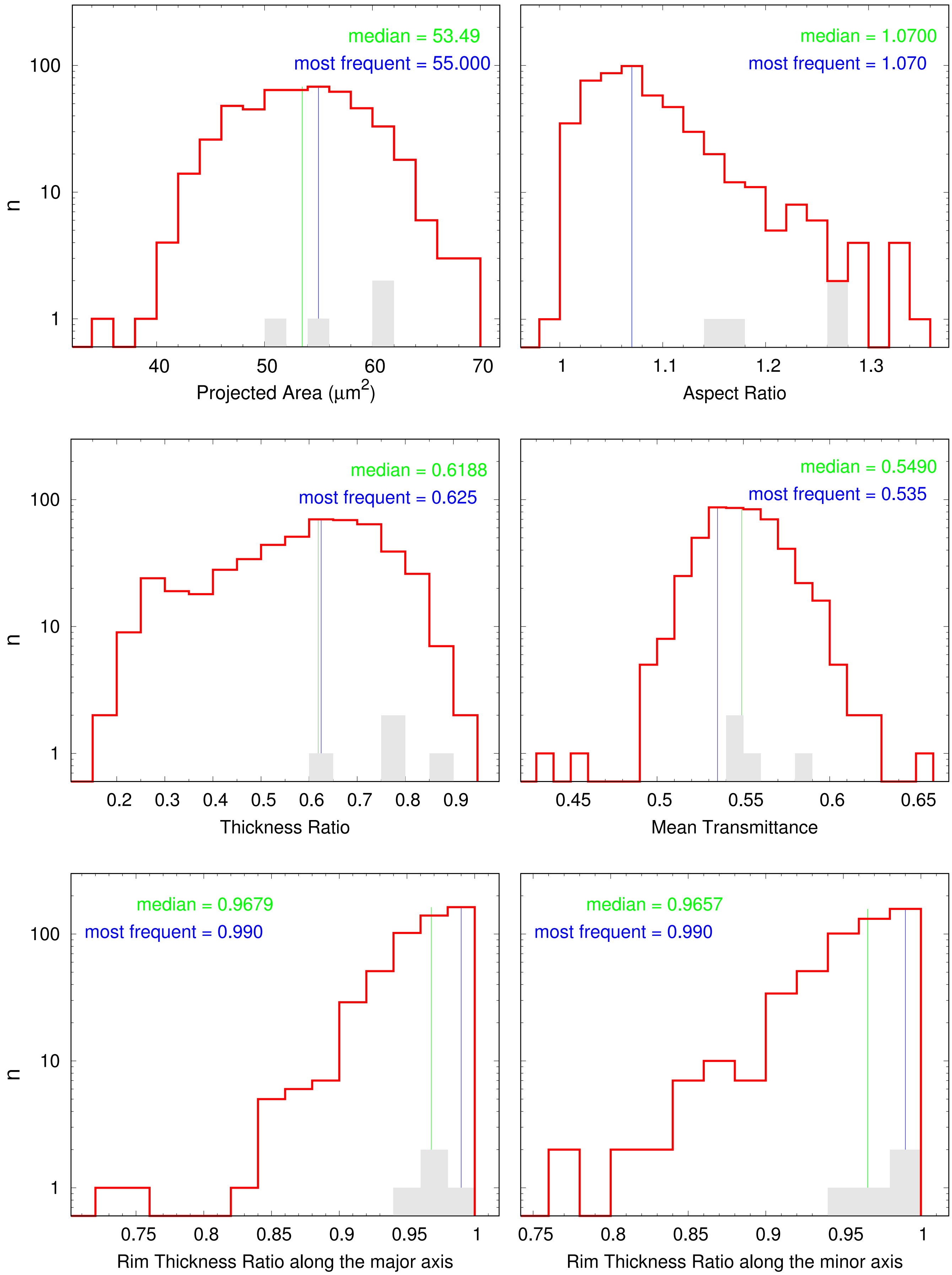

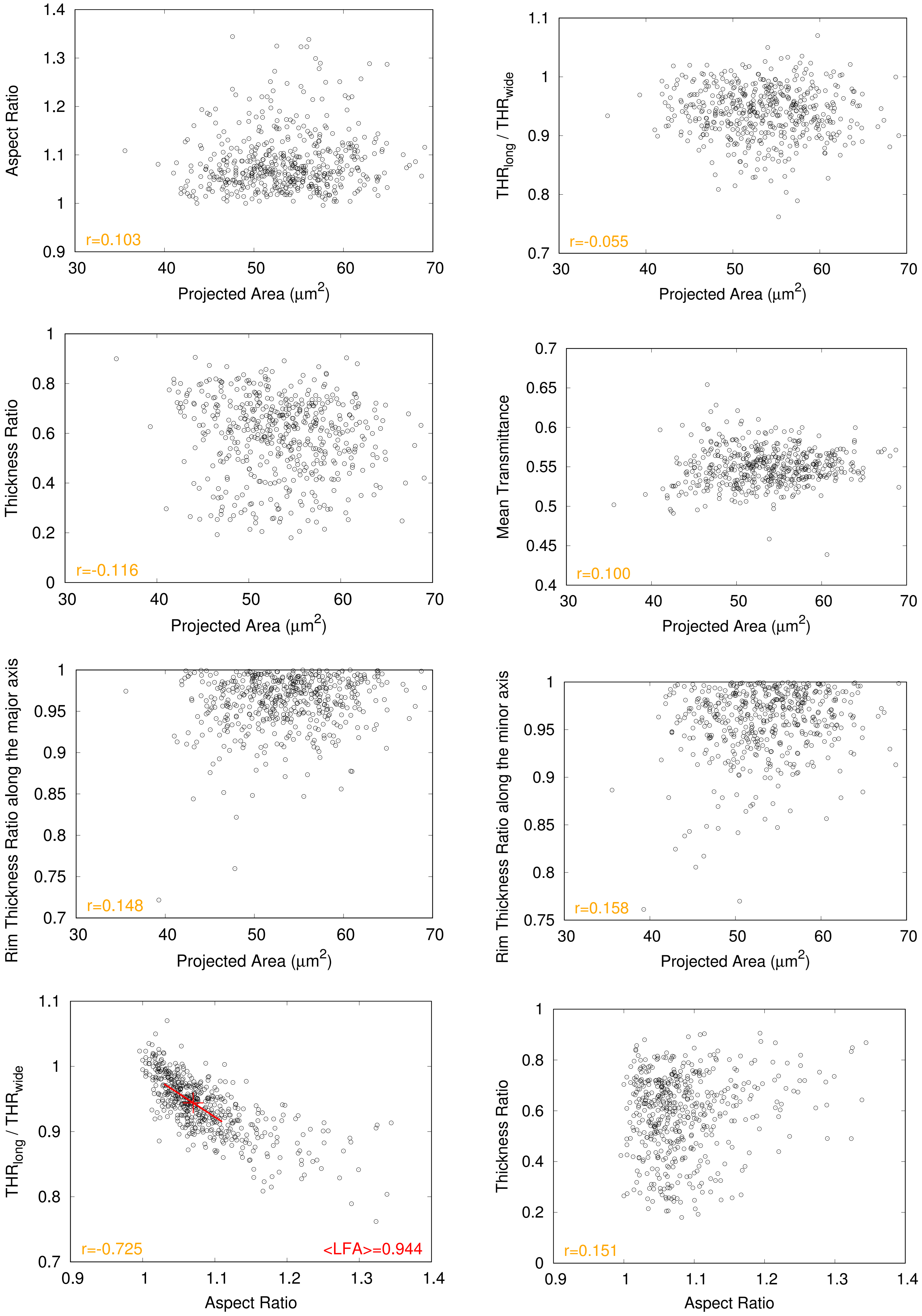

Aspect Ratio
Projected Area (μm2)
r=0.103
THRlong / THRwide
Projected Area (μm2)
r=-0.055
Thickness Ratio
Projected Area (μm2)
r=-0.116
Mean Transmittance
Projected Area (μm2)
r=0.100
Rim Thickness Ratio along the major axis
Projected Area (μm2)
r=0.148
Rim Thickness Ratio along the minor axis
Projected Area (μm2)
r=0.158
THRlong / THRwide
Aspect Ratio
r=-0.725
<LFA>=0.944
Thickness Ratio
Aspect Ratio
r=0.151

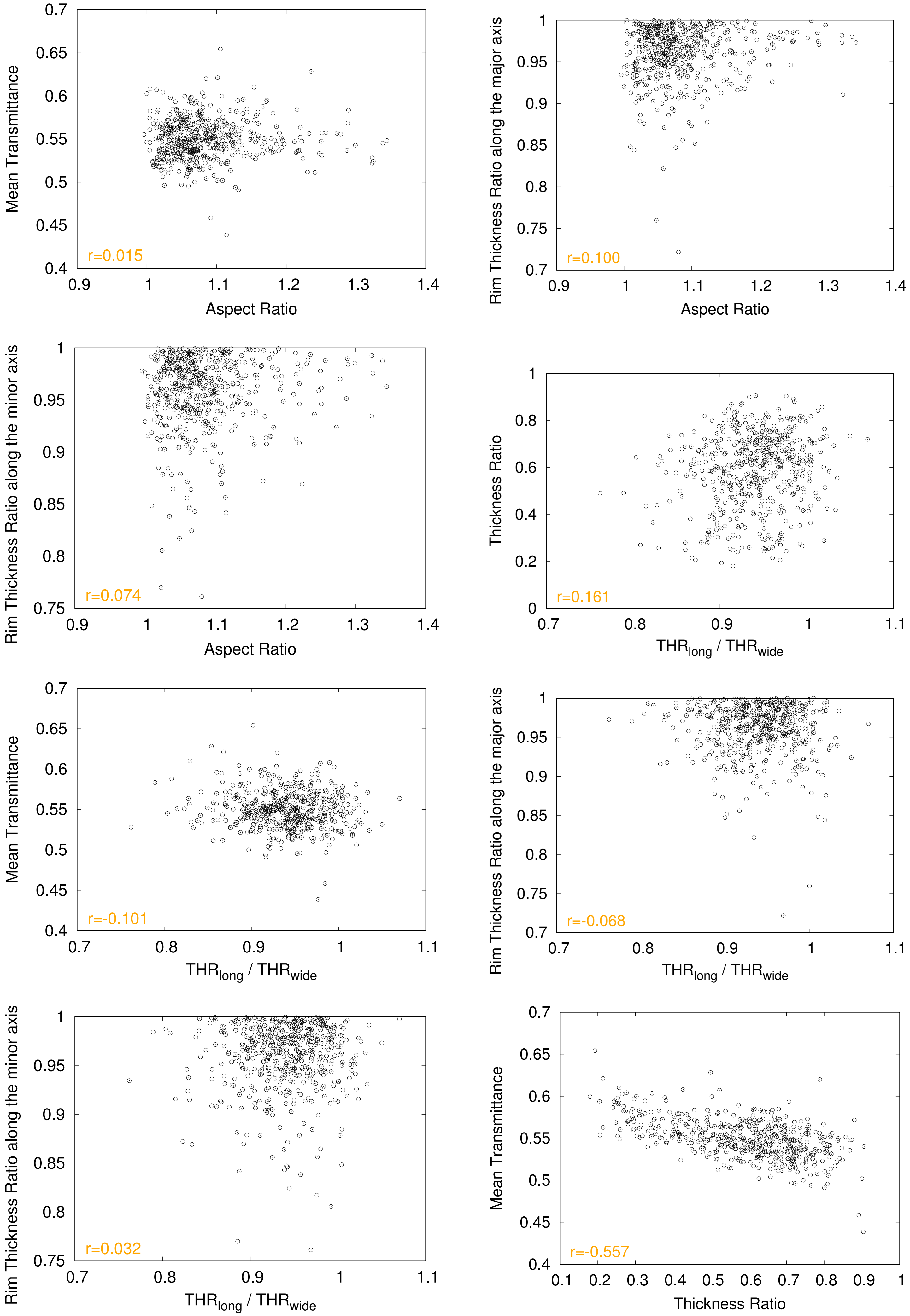
Mean Transmittance
Aspect Ratio
r=0.015
Rim Thickness Ratio along the major axis
Aspect Ratio
r=0.100
Rim Thickness Ratio along the minor axis
Aspect Ratio
r=0.074
Thickness Ratio
THR_{long} / THR_{wide}
r=0.161
Mean Transmittance
THR_{long} / THR_{wide}
r=-0.101
Rim Thickness Ratio along the major axis
THR_{long} / THR_{wide}
r=-0.068
Rim Thickness Ratio along the minor axis
THR_{long} / THR_{wide}
r=0.032
Mean Transmittance
Thickness Ratio
r=-0.557

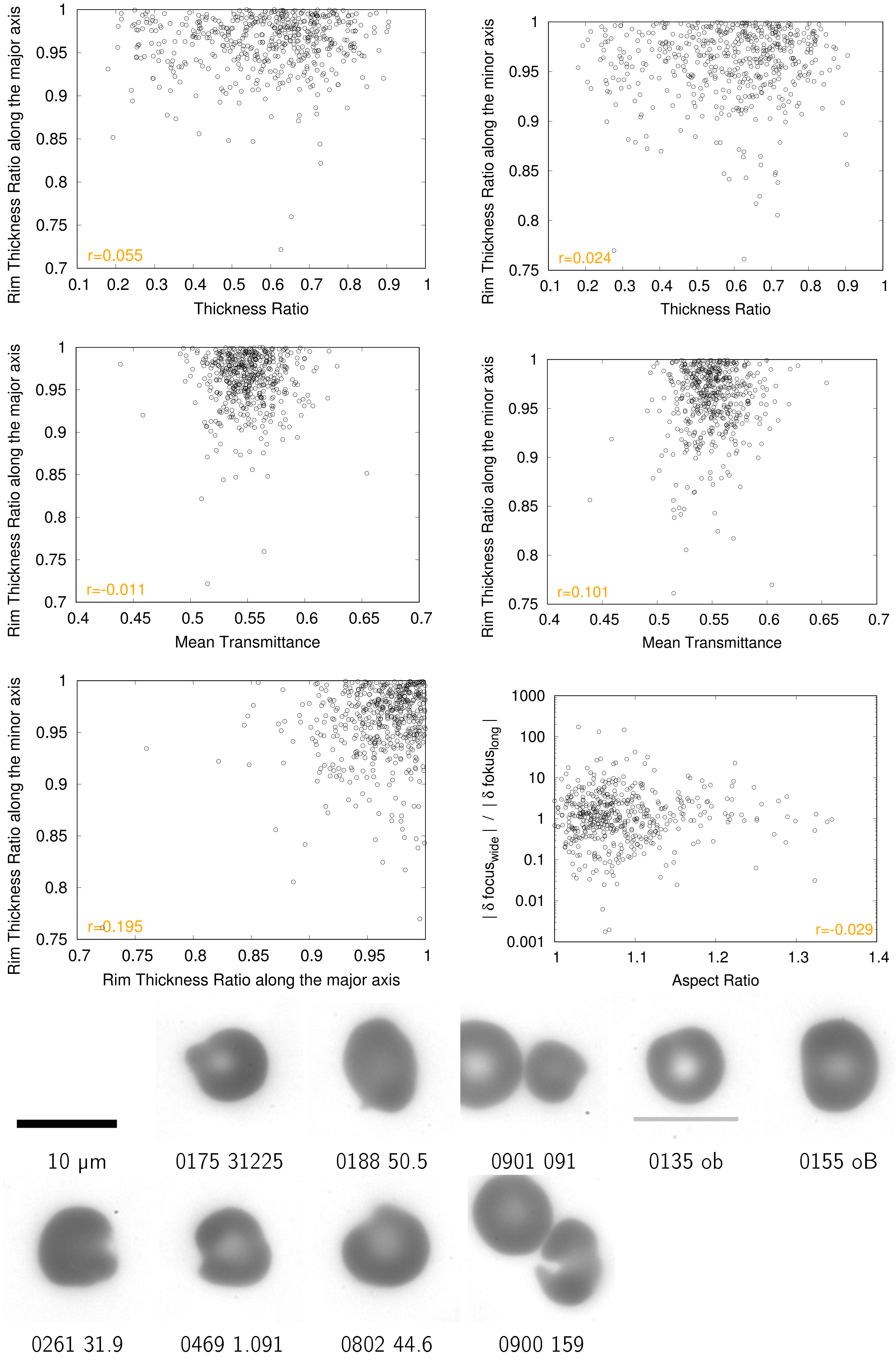
Rim Thickness Ratio along the major axis
Thickness Ratio
r=0.055
Rim Thickness Ratio along the minor axis
Thickness Ratio
r=0.024
Rim Thickness Ratio along the major axis
Mean Transmittance
r=-0.011
Rim Thickness Ratio along the minor axis
Mean Transmittance
r=0.101
Rim Thickness Ratio along the minor axis
Rim Thickness Ratio along the major axis
r=0.195
| δ focus$_{wide}$ | / | δ fokus$_{long}$ |
Aspect Ratio
r=-0.029
10 μm
0175 31225
0188 50.5
0901 091
0135 ob
0155 oB
0261 31.9
0469 1.091
0802 44.6
0900 159

Donor 2, plasma

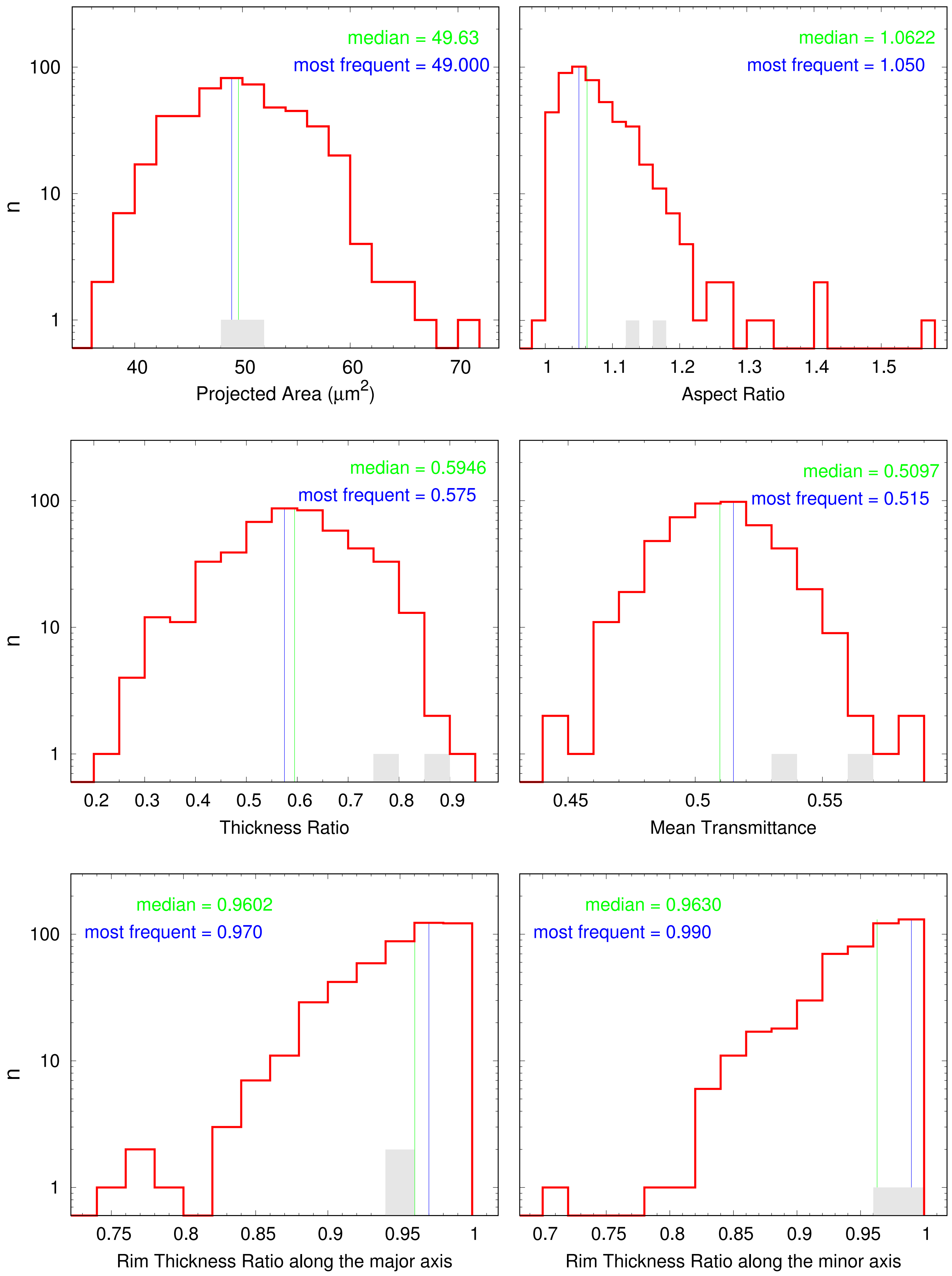

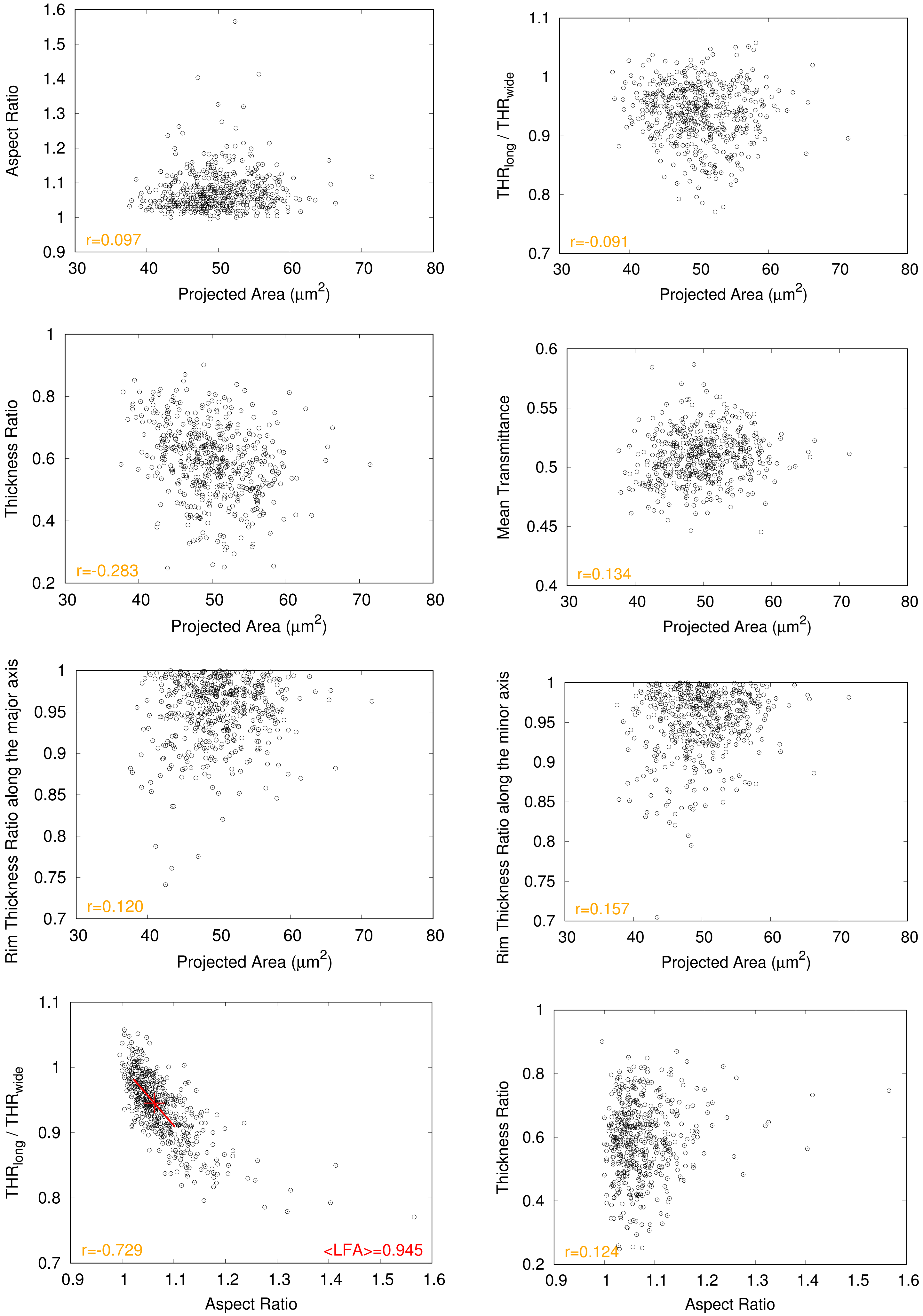

Aspect Ratio
Projected Area (μm2)
r=0.097
THRlong / THRwide
Projected Area (μm2)
r=-0.091
Thickness Ratio
Projected Area (μm2)
r=-0.283
Mean Transmittance
Projected Area (μm2)
r=0.134
Rim Thickness Ratio along the major axis
Projected Area (μm2)
r=0.120
Rim Thickness Ratio along the minor axis
Projected Area (μm2)
r=0.157
THRlong / THRwide
Aspect Ratio
r=-0.729
<LFA>=0.945
Thickness Ratio
Aspect Ratio
r=0.124

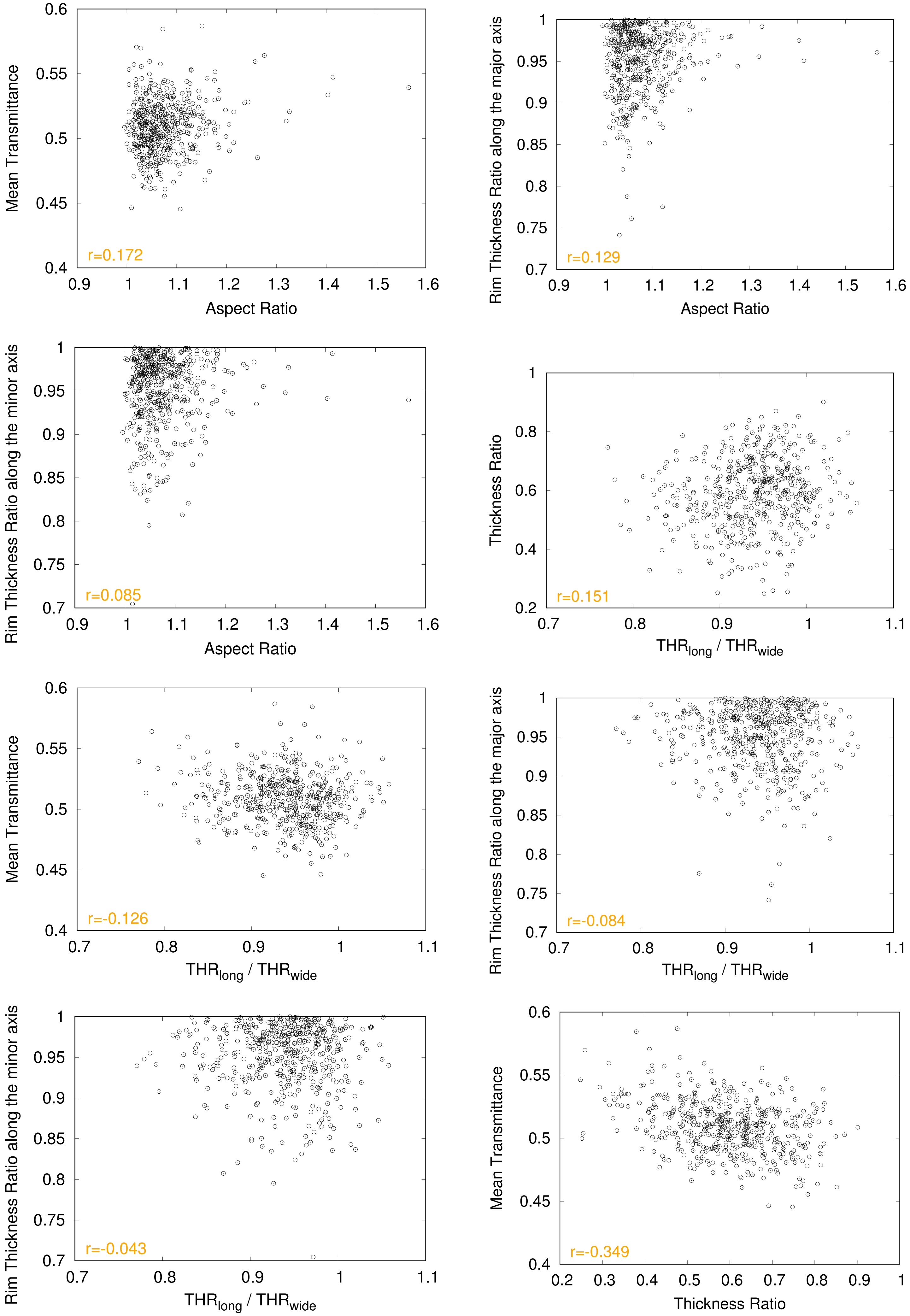

Mean Transmittance
Aspect Ratio
r=0.172
Rim Thickness Ratio along the major axis
Aspect Ratio
r=0.129
Rim Thickness Ratio along the minor axis
Aspect Ratio
r=0.085
Thickness Ratio
THR_{long} / THR_{wide}
r=0.151
Mean Transmittance
THR_{long} / THR_{wide}
r=-0.126
Rim Thickness Ratio along the major axis
THR_{long} / THR_{wide}
r=-0.084
Rim Thickness Ratio along the minor axis
THR_{long} / THR_{wide}
r=-0.043
Mean Transmittance
Thickness Ratio
r=-0.349

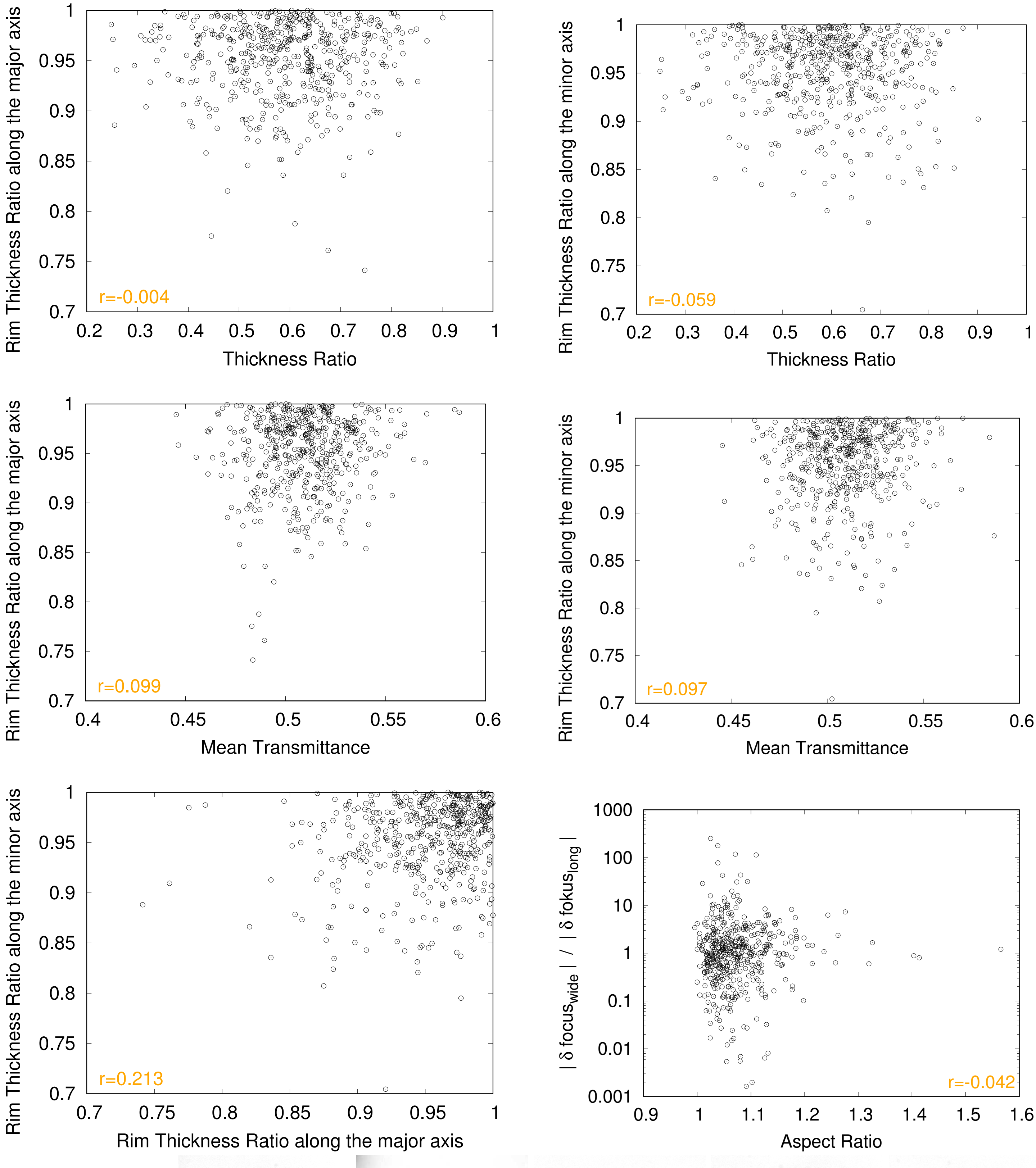

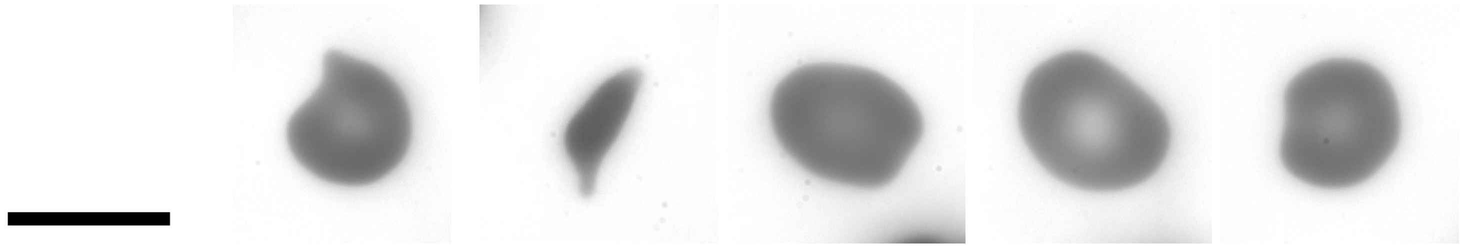

10 μm 0028 53.3 0140 018 0716 ob 0961 ob 1034 1.0095

Donor 3, plasma

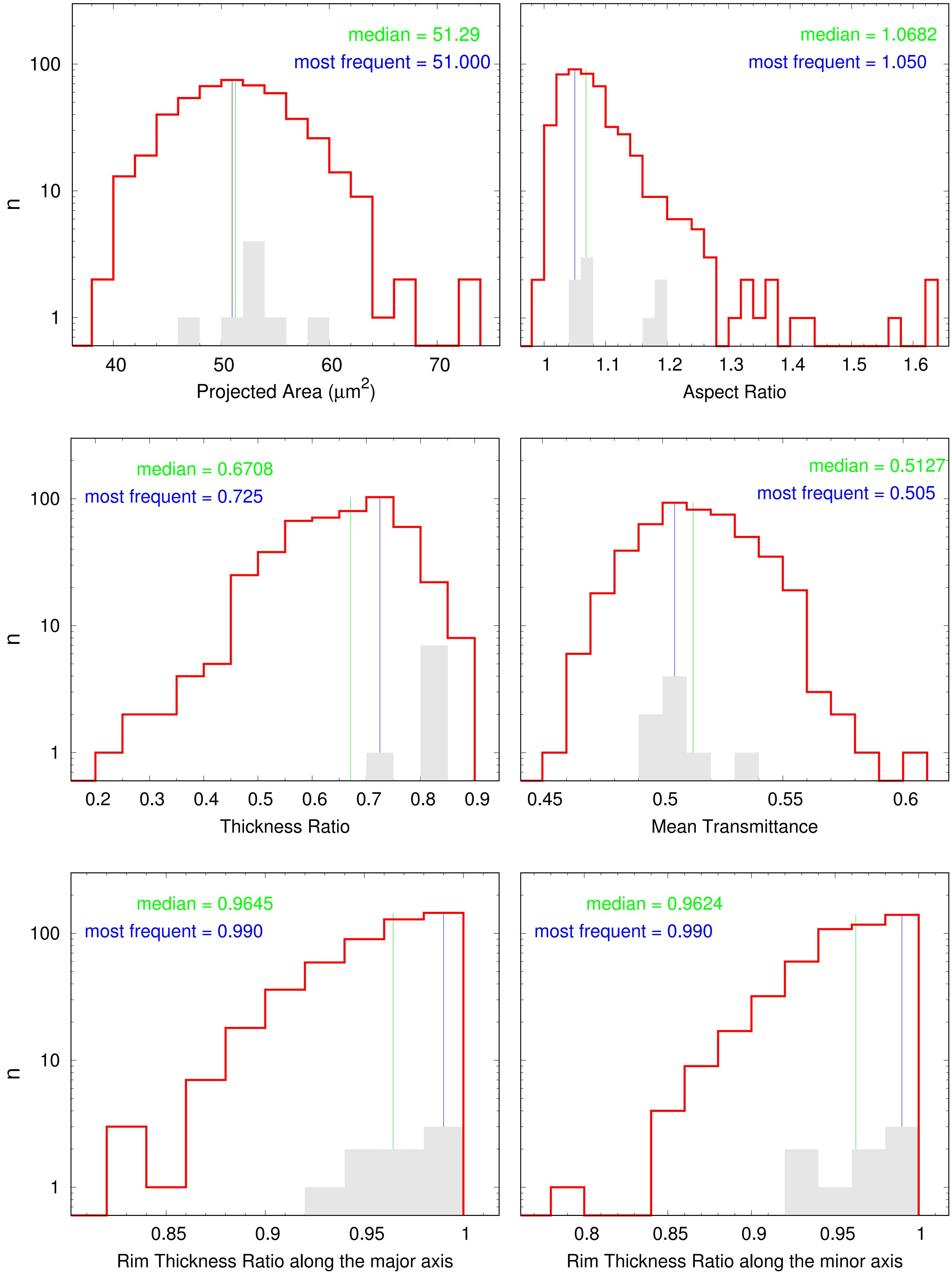

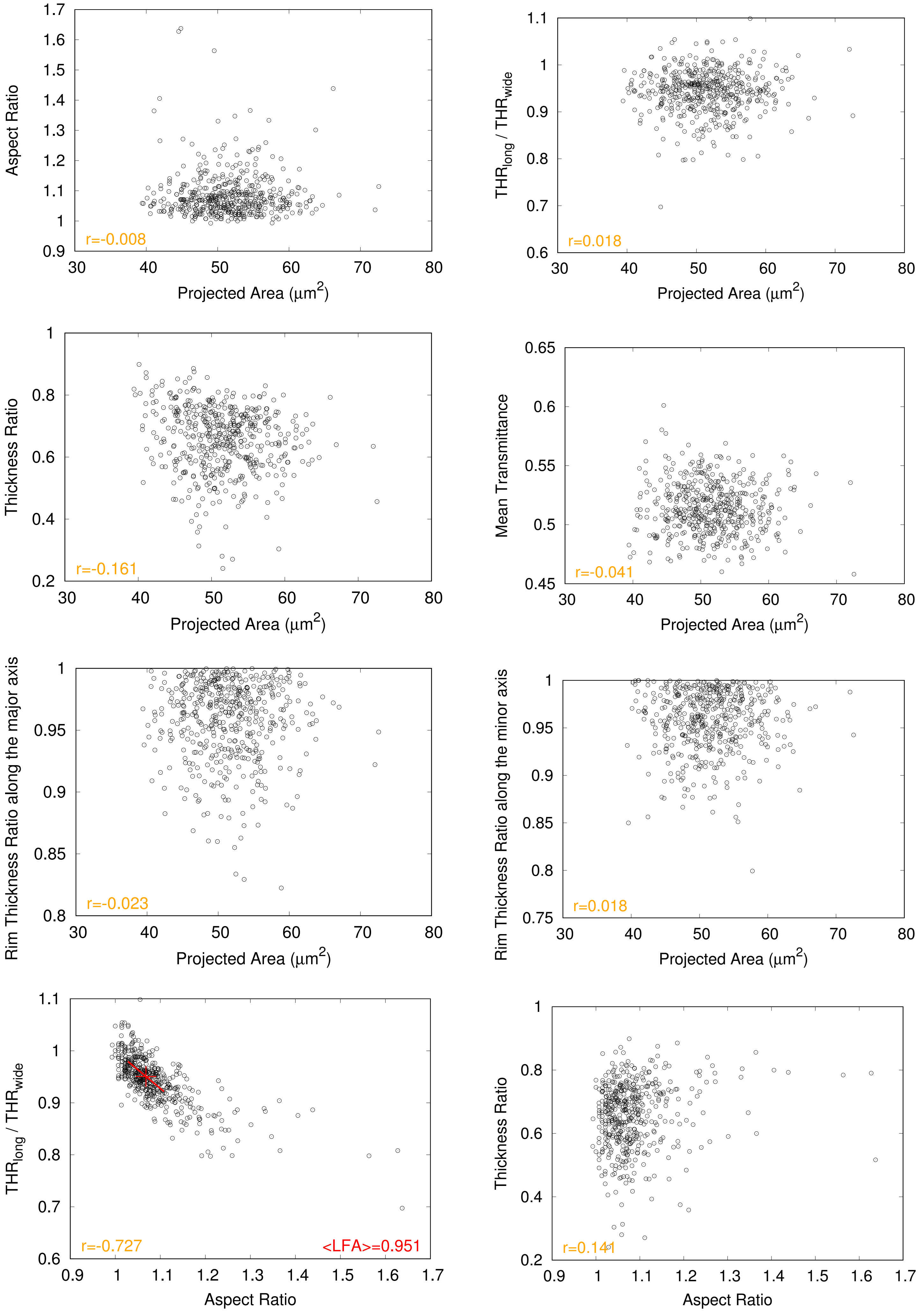

Aspect Ratio
Projected Area (μm2)
r=-0.008
THRlong / THRwide
Projected Area (μm2)
r=0.018
Thickness Ratio
Projected Area (μm2)
r=-0.161
Mean Transmittance
Projected Area (μm2)
r=-0.041
Rim Thickness Ratio along the major axis
Projected Area (μm2)
r=-0.023
Rim Thickness Ratio along the minor axis
Projected Area (μm2)
r=0.018
THRlong / THRwide
Aspect Ratio
r=-0.727
<LFA>=0.951
Thickness Ratio
Aspect Ratio
r=0.141

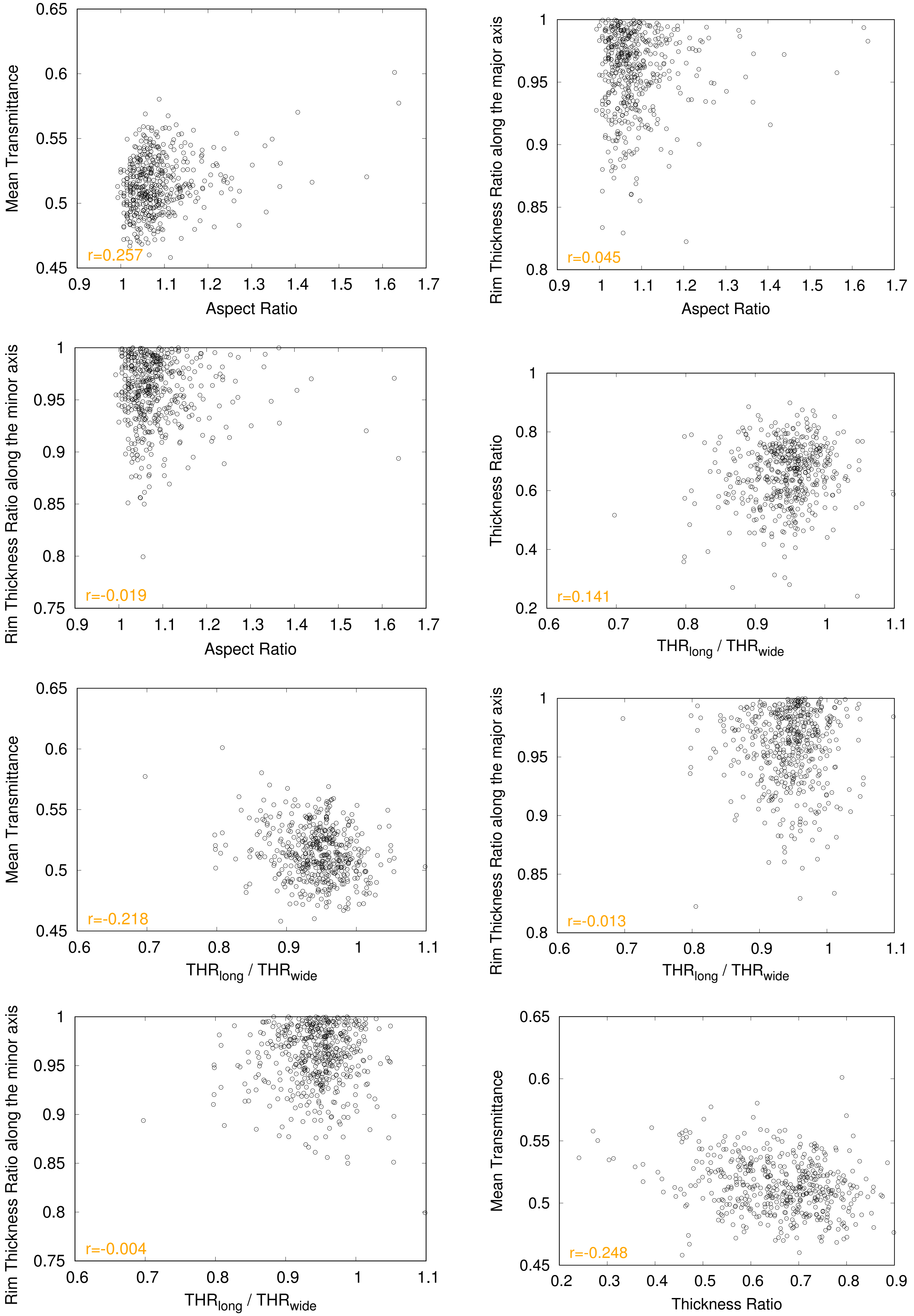
Mean Transmittance
Aspect Ratio
r=0.257
Rim Thickness Ratio along the major axis
Aspect Ratio
r=0.045
Rim Thickness Ratio along the minor axis
Aspect Ratio
r=-0.019
Thickness Ratio
THR_{long} / THR_{wide}
r=0.141
Mean Transmittance
THR_{long} / THR_{wide}
r=-0.218
Rim Thickness Ratio along the major axis
THR_{long} / THR_{wide}
r=-0.013
Rim Thickness Ratio along the minor axis
THR_{long} / THR_{wide}
r=-0.004
Mean Transmittance
Thickness Ratio
r=-0.248

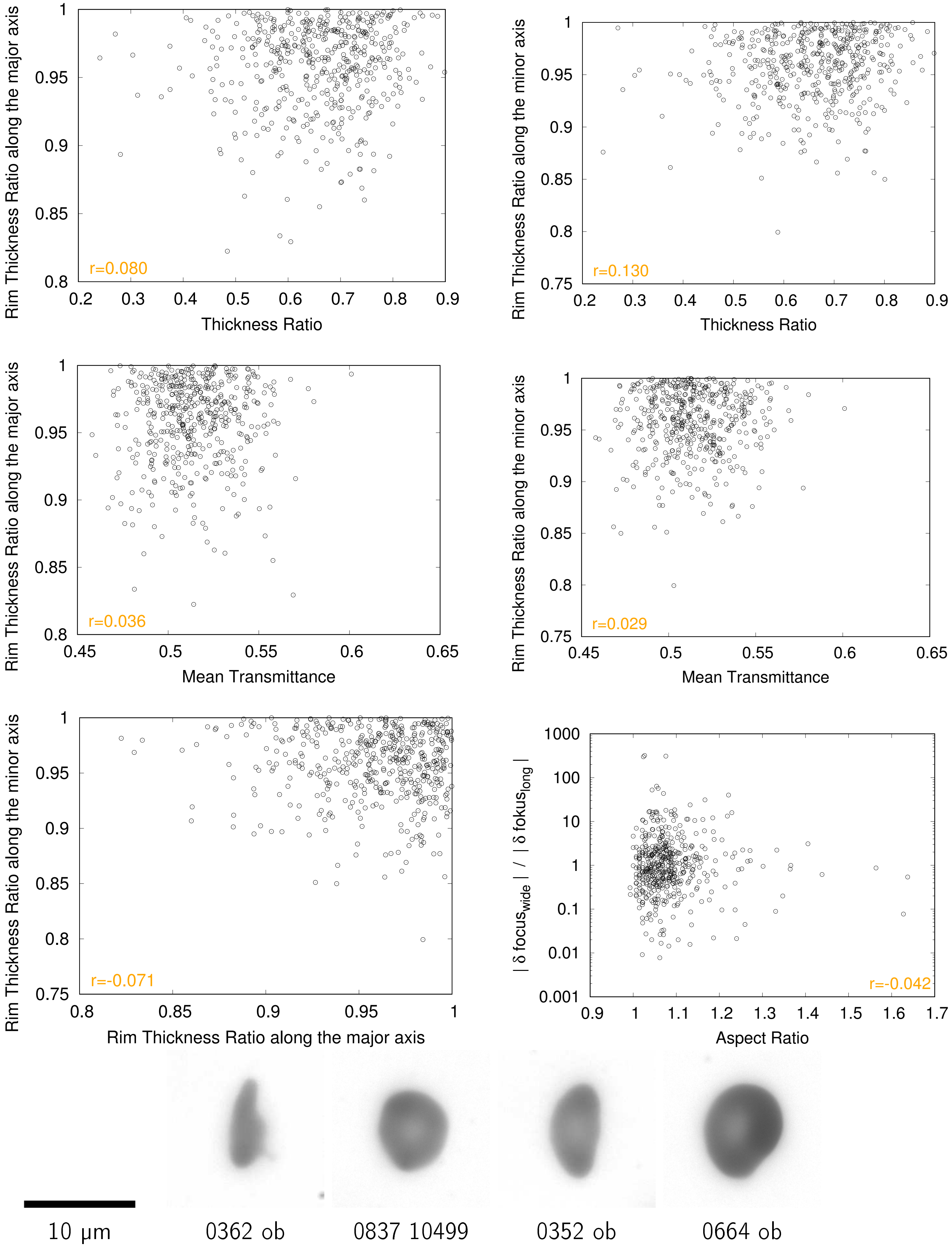

Rim Thickness Ratio along the major axis
Thickness Ratio
r=0.080
Rim Thickness Ratio along the minor axis
Thickness Ratio
r=0.130
Rim Thickness Ratio along the major axis
Mean Transmittance
r=0.036
Rim Thickness Ratio along the minor axis
Mean Transmittance
r=0.029
Rim Thickness Ratio along the minor axis
Rim Thickness Ratio along the major axis
r=-0.071
| δ focus_wide | / | δ fokus_long |
Aspect Ratio
r=-0.042
10 μm
0362 ob
0837 10499
0352 ob
0664 ob

Donor 3, serum

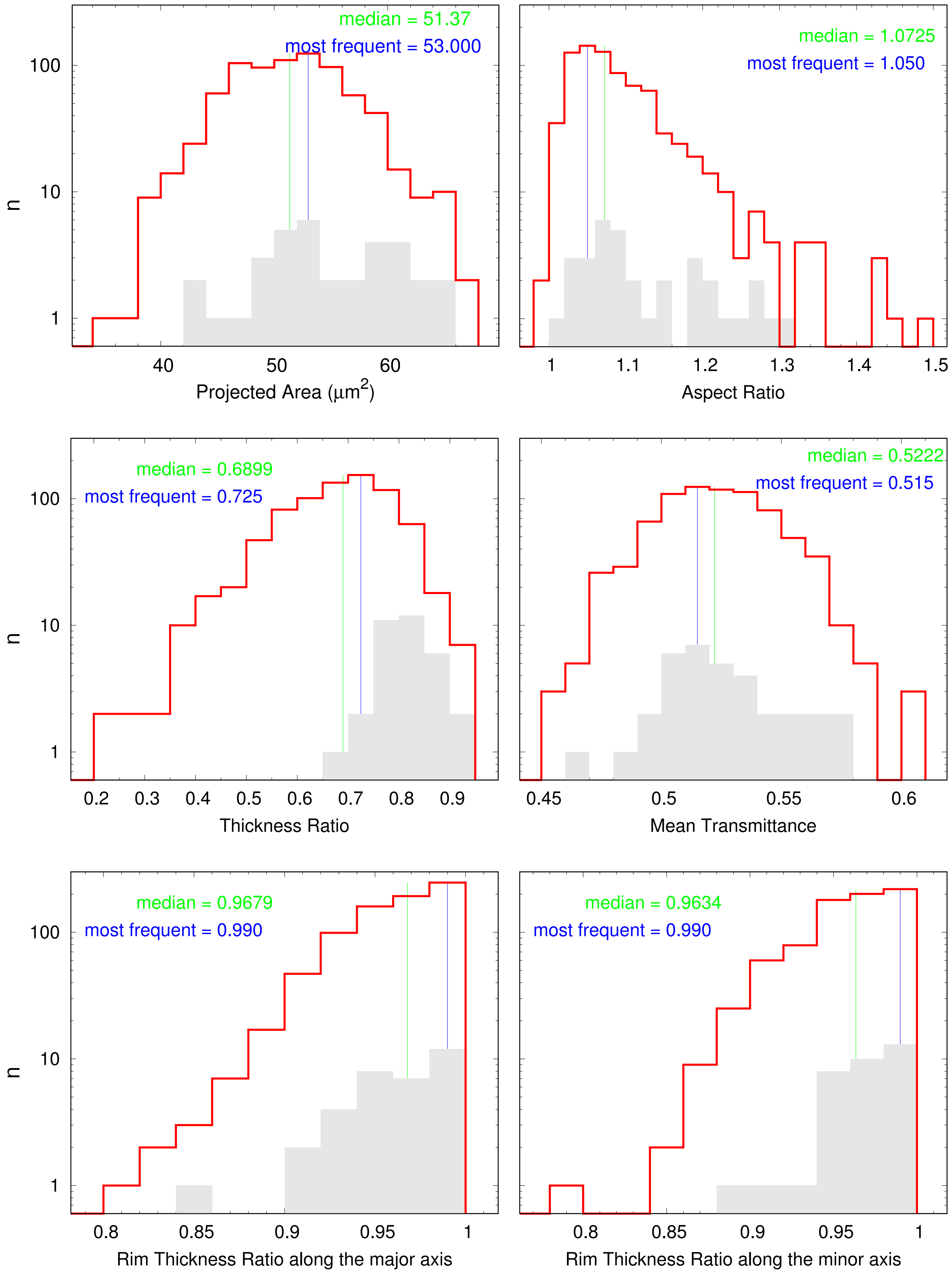

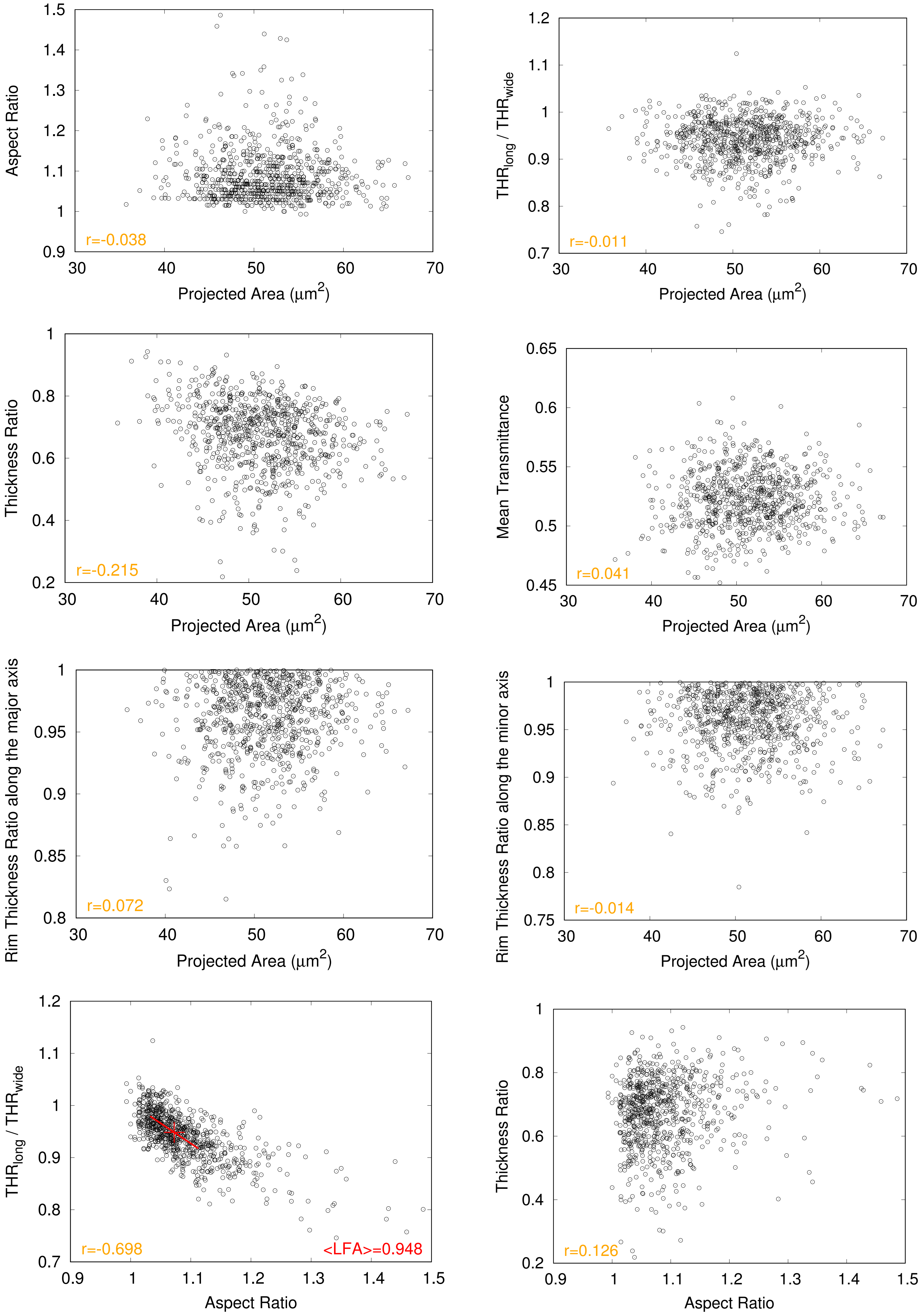

Aspect Ratio
Projected Area (μm²)
r=-0.038
THR_long / THR_wide
Projected Area (μm²)
r=-0.011
Thickness Ratio
Projected Area (μm²)
r=-0.215
Mean Transmittance
Projected Area (μm²)
r=0.041
Rim Thickness Ratio along the major axis
Projected Area (μm²)
r=0.072
Rim Thickness Ratio along the minor axis
Projected Area (μm²)
r=-0.014
THR_long / THR_wide
Aspect Ratio
r=-0.698
<LFA>=0.948
Thickness Ratio
Aspect Ratio
r=0.126

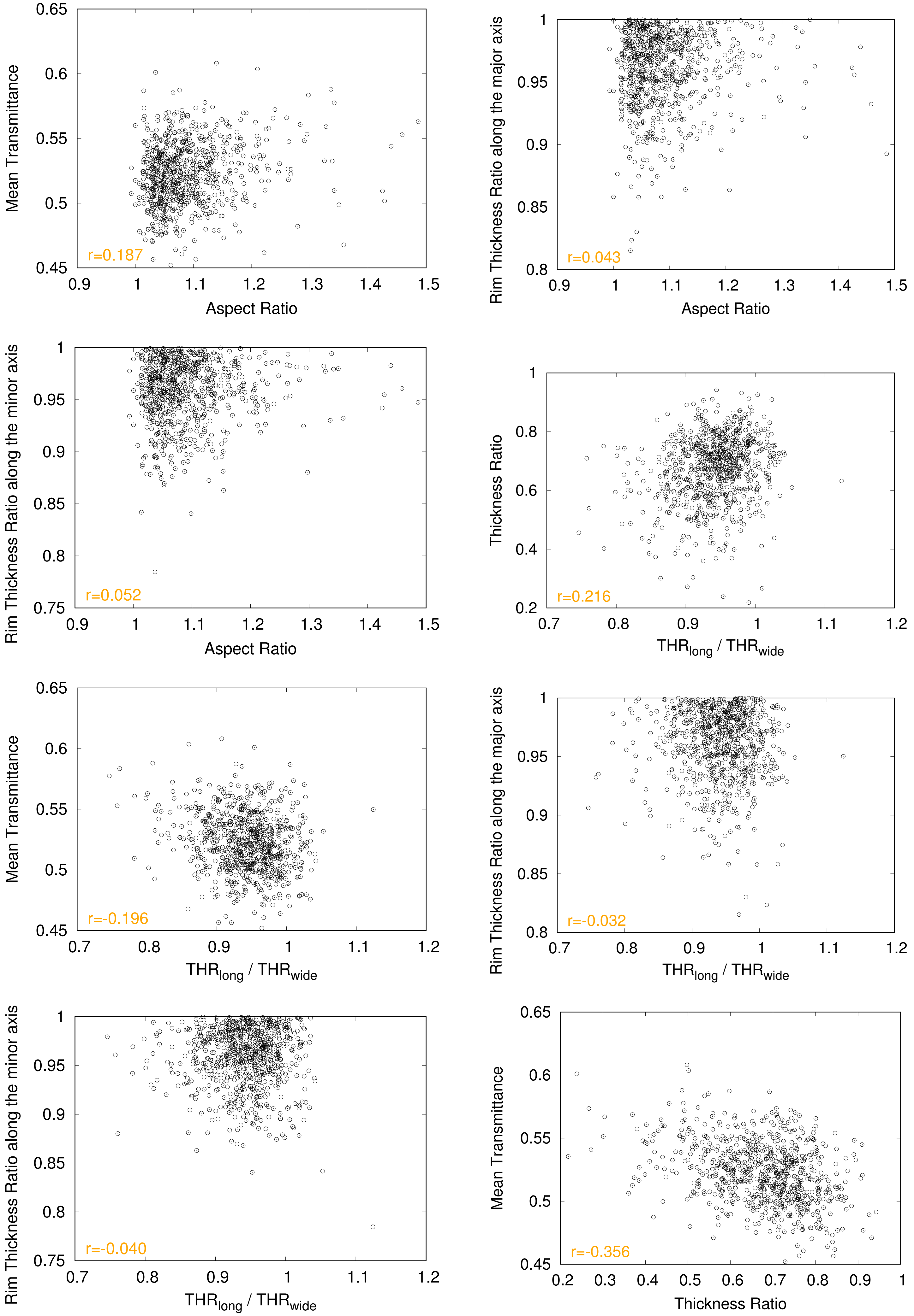
Mean Transmittance
Aspect Ratio
r=0.187
Rim Thickness Ratio along the major axis
Aspect Ratio
r=0.043
Rim Thickness Ratio along the minor axis
Aspect Ratio
r=0.052
Thickness Ratio
THR_{long} / THR_{wide}
r=0.216
Mean Transmittance
THR_{long} / THR_{wide}
r=-0.196
Rim Thickness Ratio along the major axis
THR_{long} / THR_{wide}
r=-0.032
Rim Thickness Ratio along the minor axis
THR_{long} / THR_{wide}
r=-0.040
Mean Transmittance
Thickness Ratio
r=-0.356

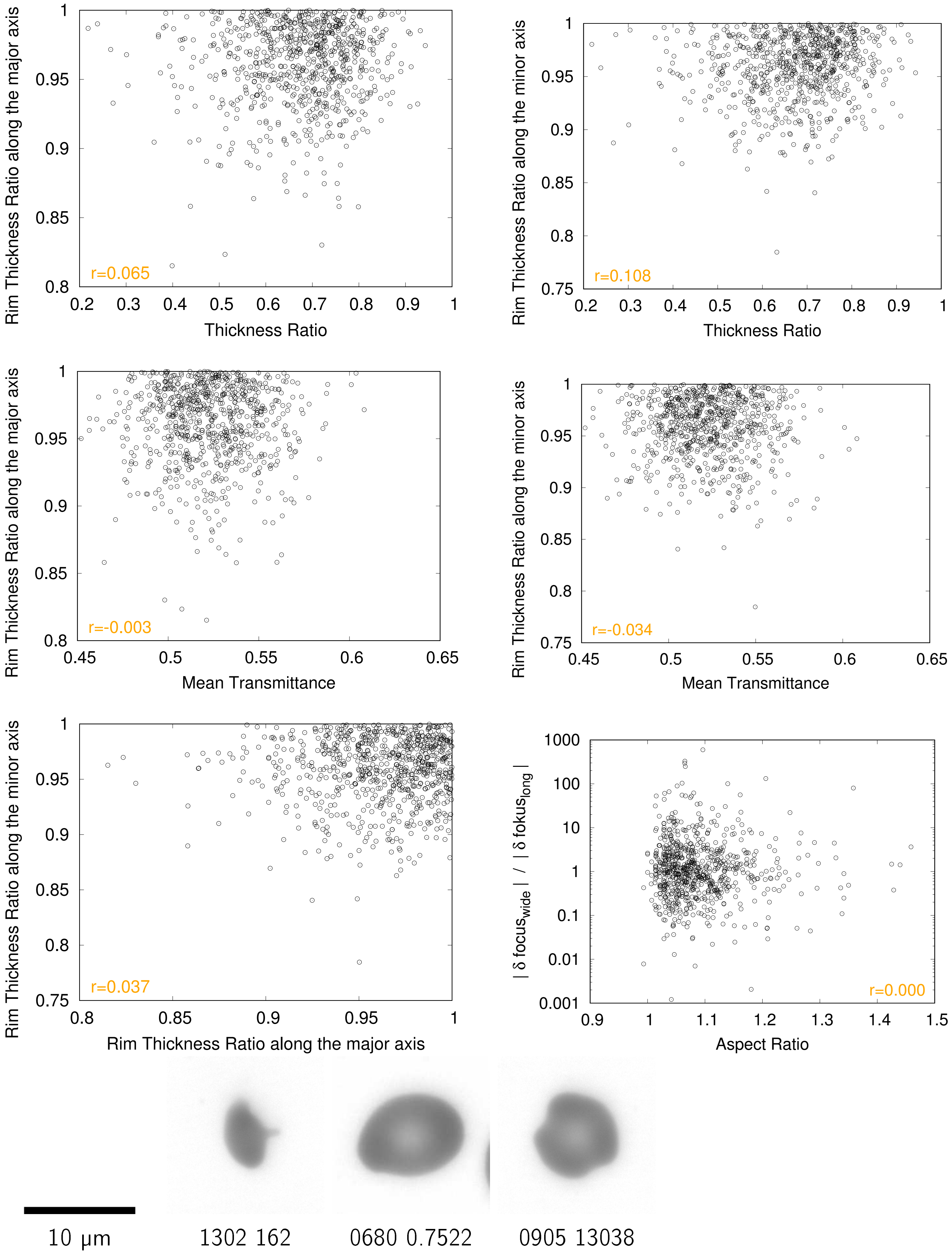

Rim Thickness Ratio along the major axis
Thickness Ratio
r=0.065
Rim Thickness Ratio along the minor axis
Thickness Ratio
r=0.108
Rim Thickness Ratio along the major axis
Mean Transmittance
r=-0.003
Rim Thickness Ratio along the minor axis
Mean Transmittance
r=-0.034
Rim Thickness Ratio along the minor axis
Rim Thickness Ratio along the major axis
r=0.037
$|\delta \, focus_{wide}| \, / \, |\delta \, fokus_{long}|$
Aspect Ratio
r=0.000
10 μm
1302 162
0680 0.7522
0905 13038

Donor 3, serum washed

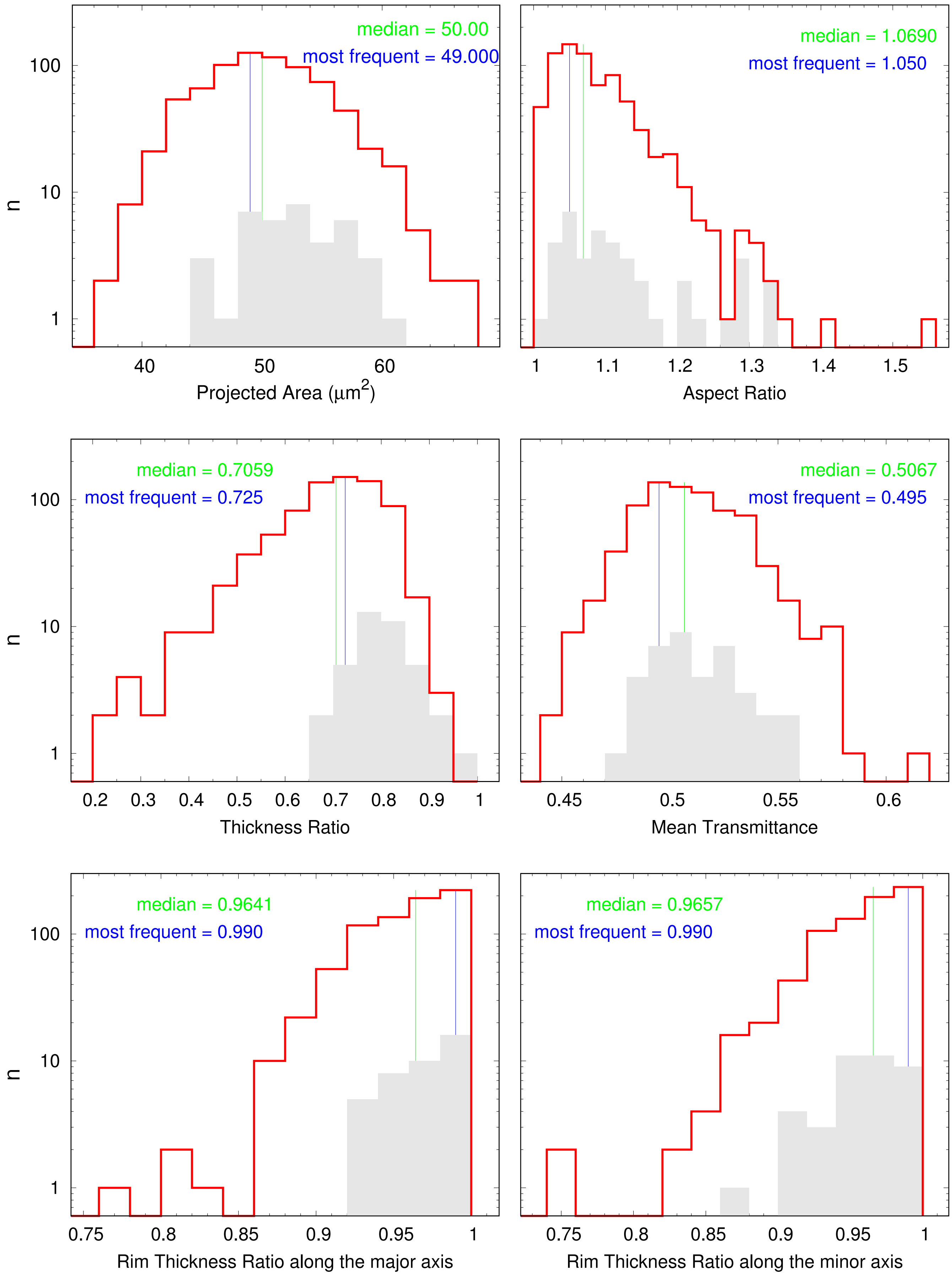

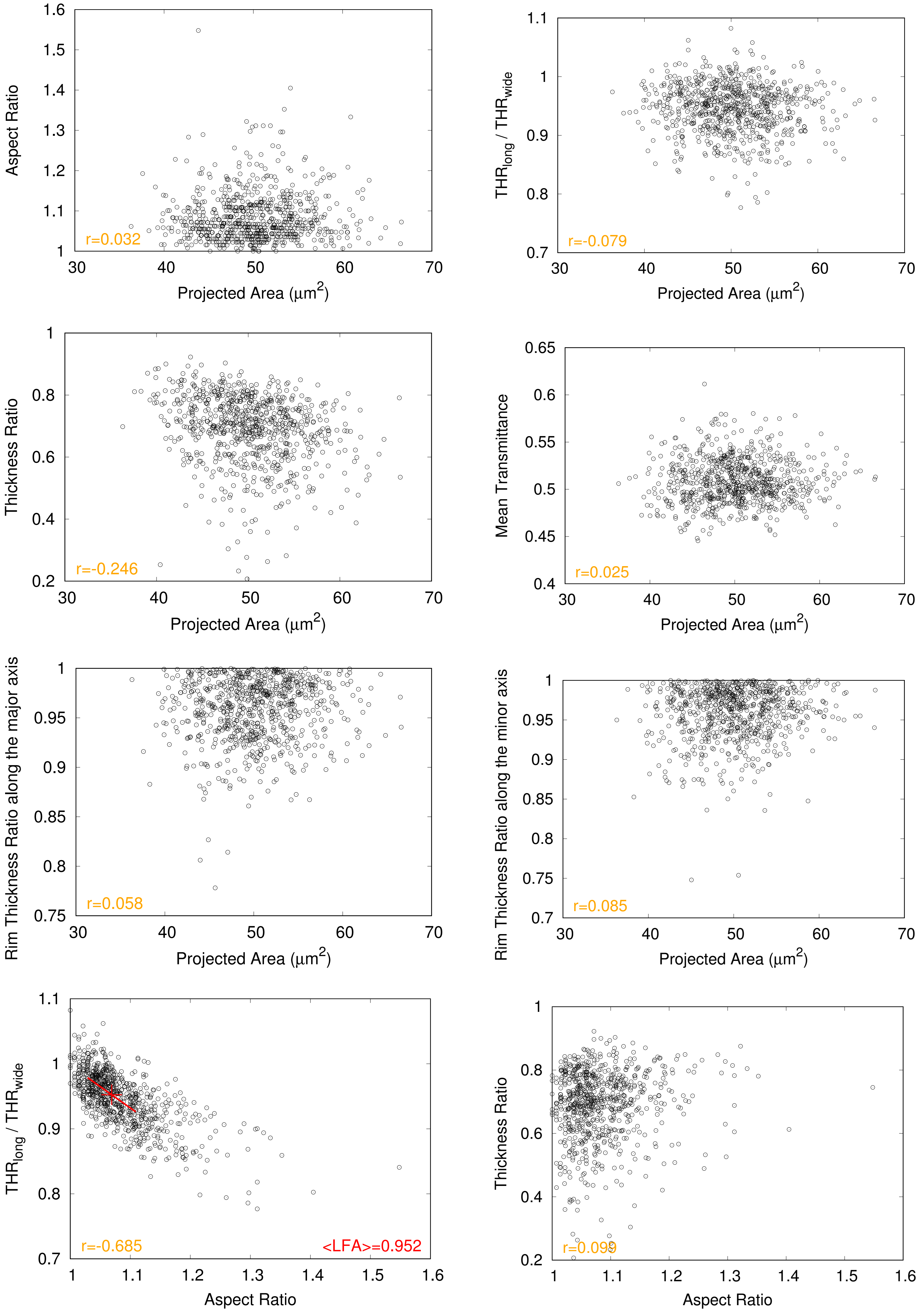

Aspect Ratio
Projected Area (μm2)
r=0.032
THRlong / THRwide
Projected Area (μm2)
r=-0.079
Thickness Ratio
Projected Area (μm2)
r=-0.246
Mean Transmittance
Projected Area (μm2)
r=0.025
Rim Thickness Ratio along the major axis
Projected Area (μm2)
r=0.058
Rim Thickness Ratio along the minor axis
Projected Area (μm2)
r=0.085
THRlong / THRwide
Aspect Ratio
r=-0.685
<LFA>=0.952
Thickness Ratio
Aspect Ratio
r=0.099

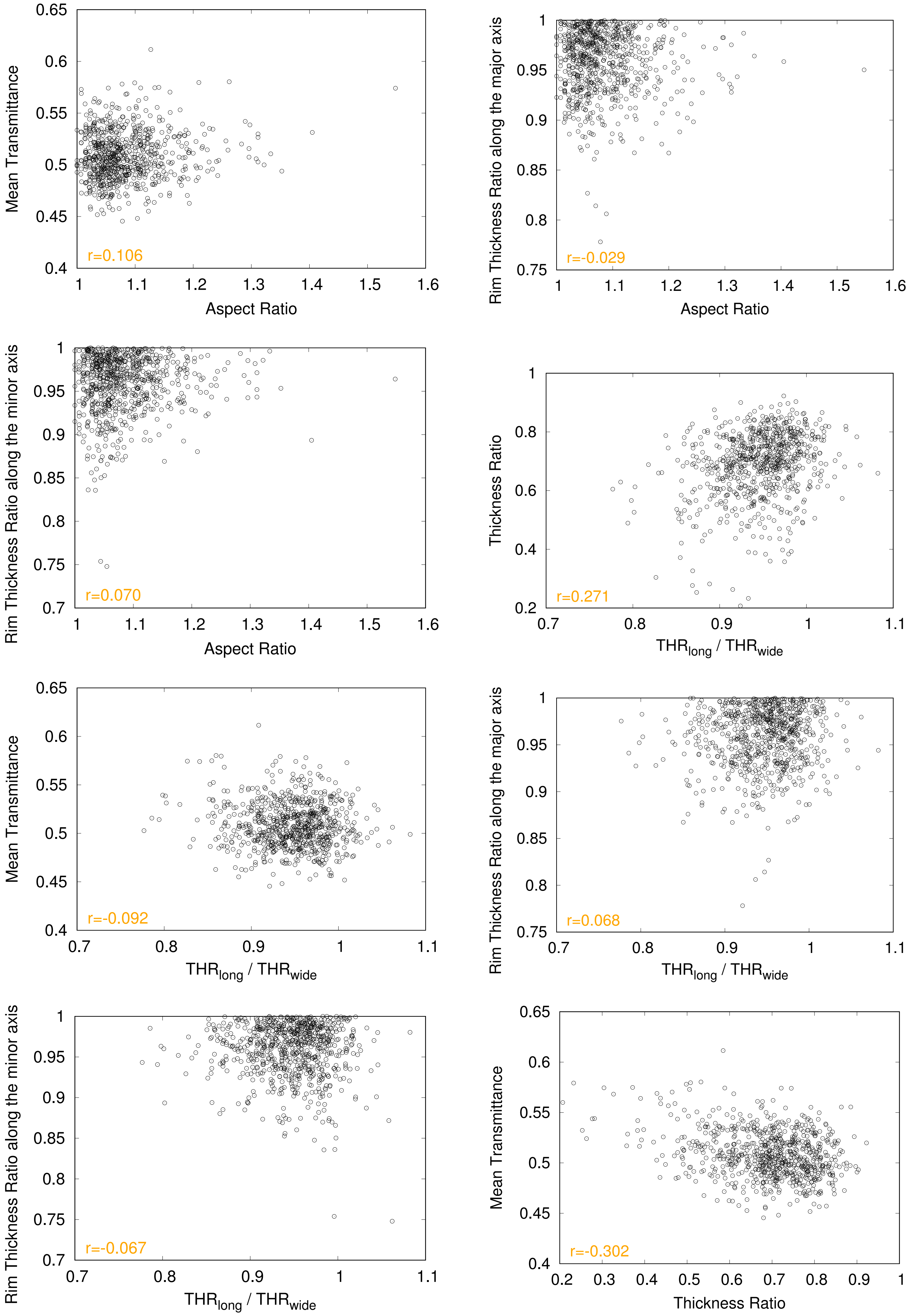

Mean Transmittance
Aspect Ratio
r=0.106
Rim Thickness Ratio along the major axis
Aspect Ratio
r=-0.029
Rim Thickness Ratio along the minor axis
Aspect Ratio
r=0.070
Thickness Ratio
THR_long / THR_wide
r=0.271
Mean Transmittance
THR_long / THR_wide
r=-0.092
Rim Thickness Ratio along the major axis
THR_long / THR_wide
r=0.068
Rim Thickness Ratio along the minor axis
THR_long / THR_wide
r=-0.067
Mean Transmittance
Thickness Ratio
r=-0.302

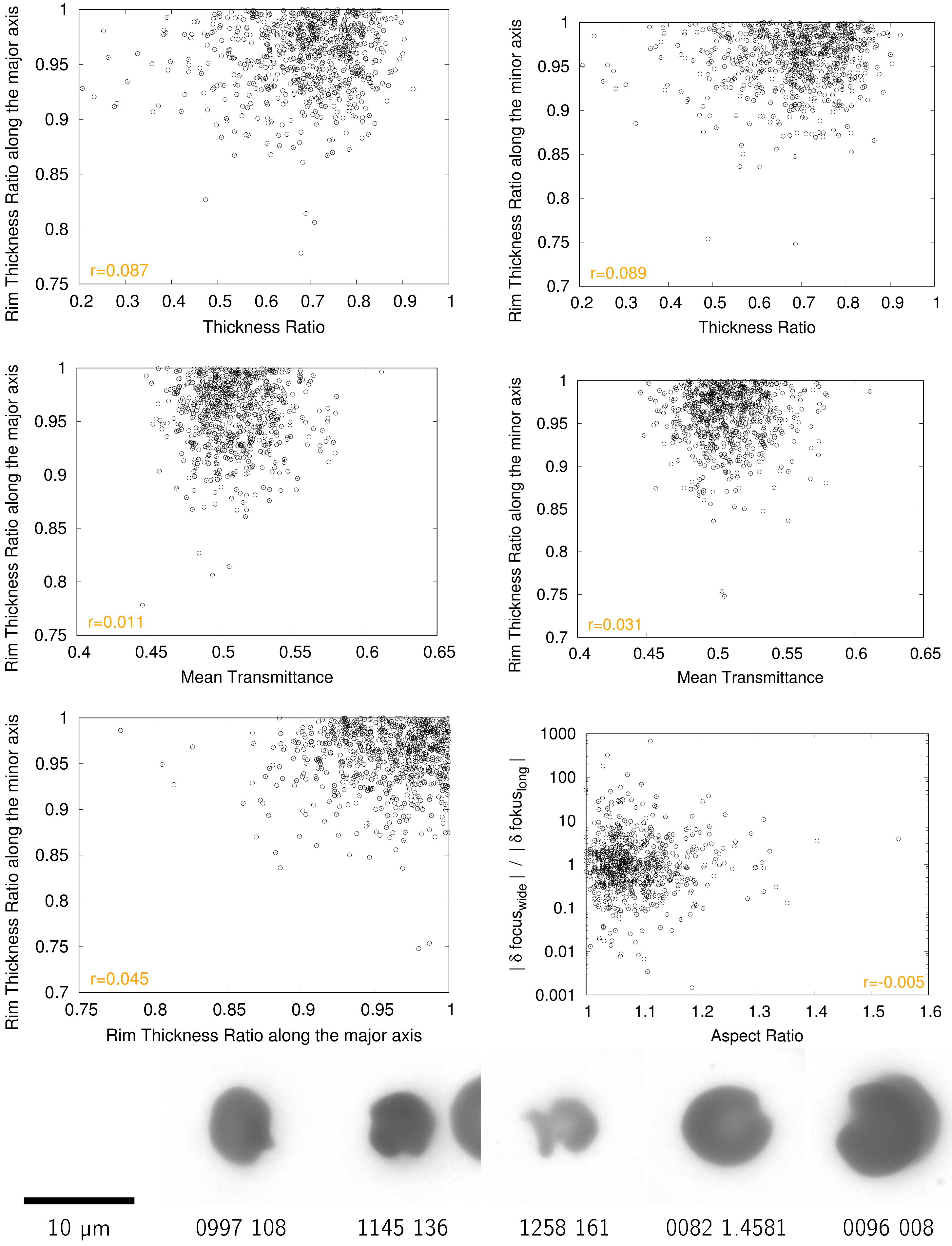
Rim Thickness Ratio along the major axis
Thickness Ratio
r=0.087
Rim Thickness Ratio along the minor axis
Thickness Ratio
r=0.089
Rim Thickness Ratio along the major axis
Mean Transmittance
r=0.011
Rim Thickness Ratio along the minor axis
Mean Transmittance
r=0.031
Rim Thickness Ratio along the minor axis
Rim Thickness Ratio along the major axis
r=0.045
| δ focus$_{wide}$ | / | δ fokus$_{long}$ |
Aspect Ratio
r=-0.005
10 μm
0997 108
1145 136
1258 161
0082 1.4581
0096 008

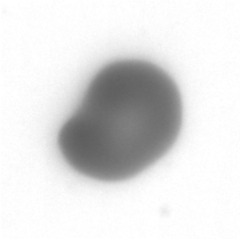

0113 0.8897

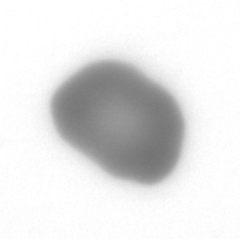

0191 017

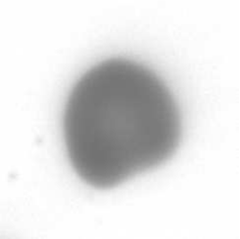

0404 11876

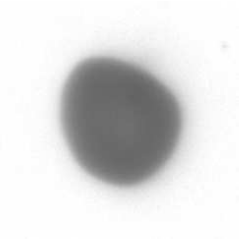

0449 12472

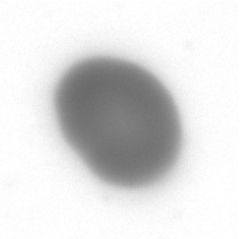

0496 0.6632

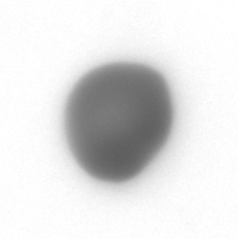

1166 140

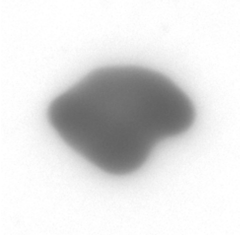

1219 152

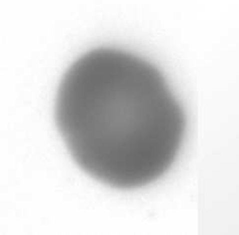

1273 0.9223

Donor 4, plasma

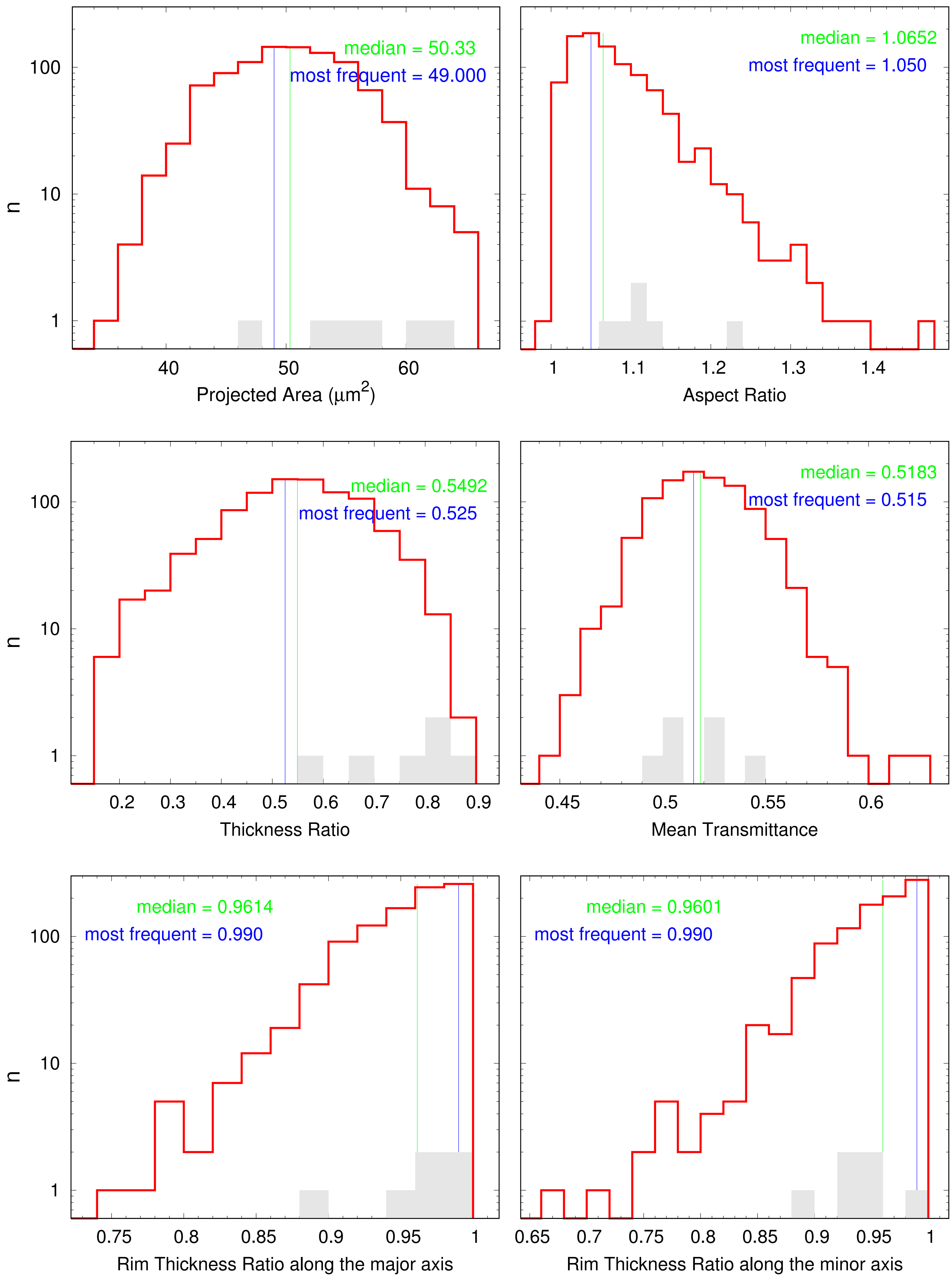

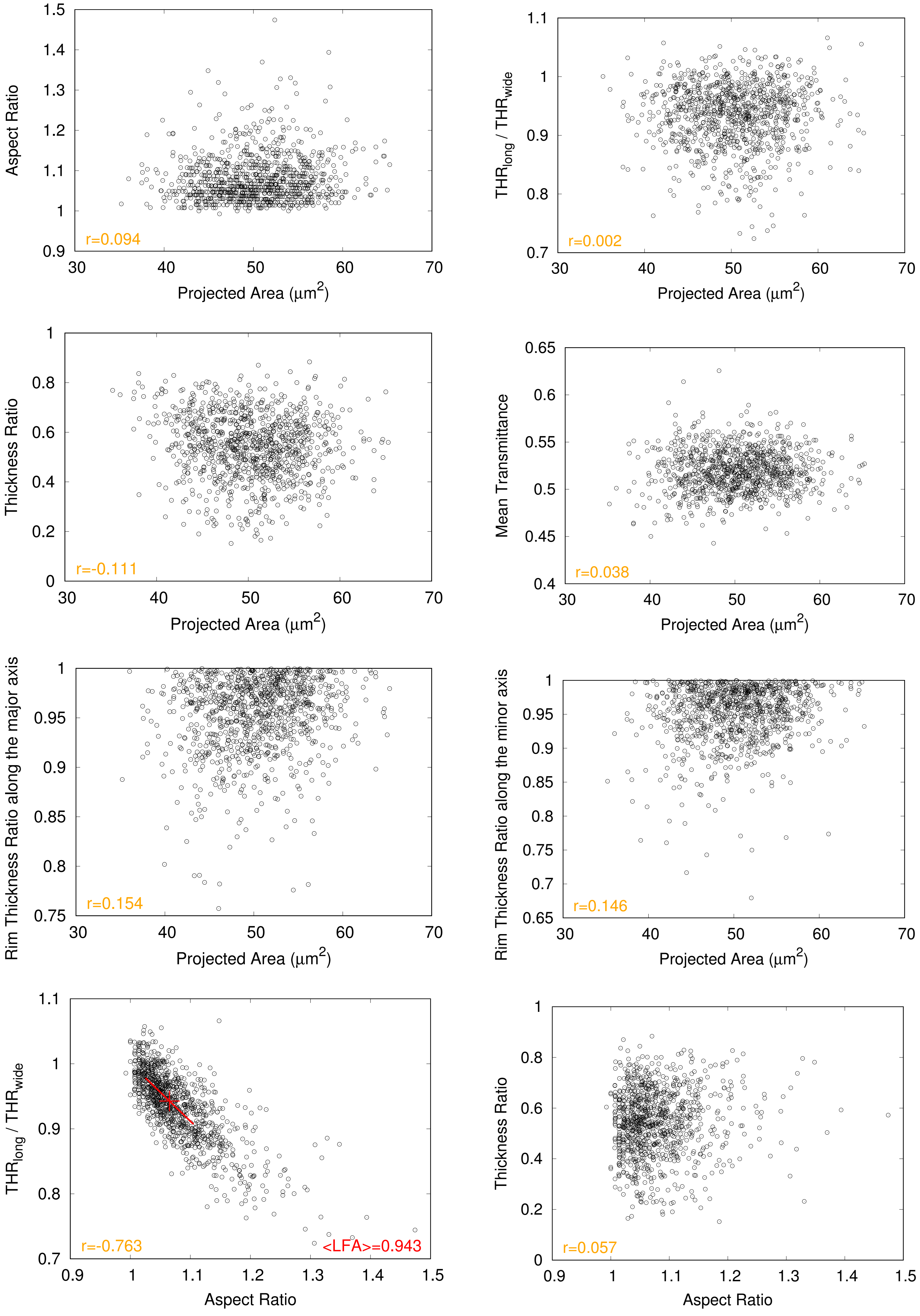
Aspect Ratio
Projected Area (μm²)
r=0.094
THR_long / THR_wide
Projected Area (μm²)
r=0.002
Thickness Ratio
Projected Area (μm²)
r=-0.111
Mean Transmittance
Projected Area (μm²)
r=0.038
Rim Thickness Ratio along the major axis
Projected Area (μm²)
r=0.154
Rim Thickness Ratio along the minor axis
Projected Area (μm²)
r=0.146
THR_long / THR_wide
Aspect Ratio
r=-0.763
<LFA>=0.943
Thickness Ratio
Aspect Ratio
r=0.057

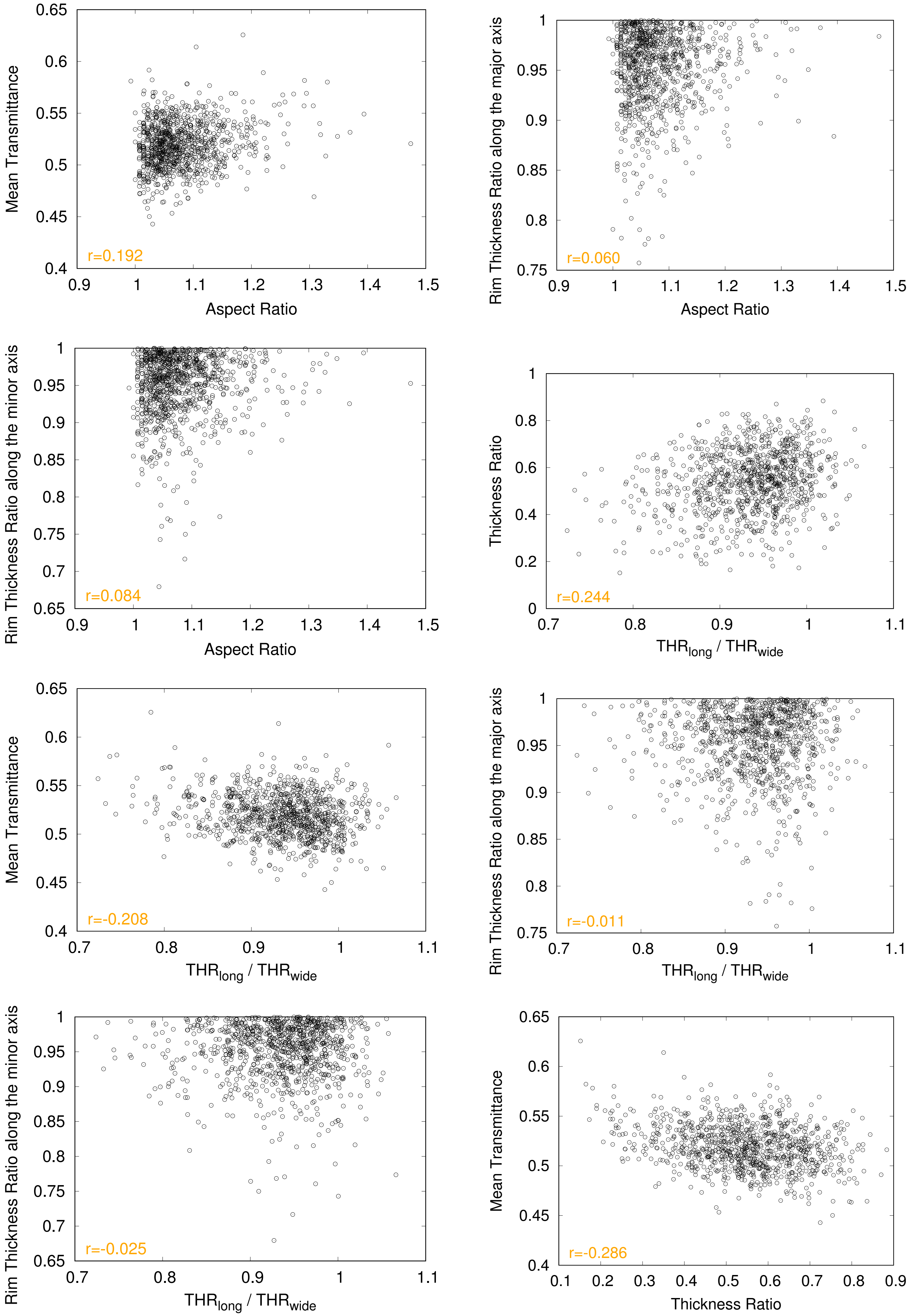

Mean Transmittance
Aspect Ratio
r=0.192
Rim Thickness Ratio along the major axis
Aspect Ratio
r=0.060
Rim Thickness Ratio along the minor axis
Aspect Ratio
r=0.084
Thickness Ratio
THR_long / THR_wide
r=0.244
Mean Transmittance
THR_long / THR_wide
r=-0.208
Rim Thickness Ratio along the major axis
THR_long / THR_wide
r=-0.011
Rim Thickness Ratio along the minor axis
THR_long / THR_wide
r=-0.025
Mean Transmittance
Thickness Ratio
r=-0.286

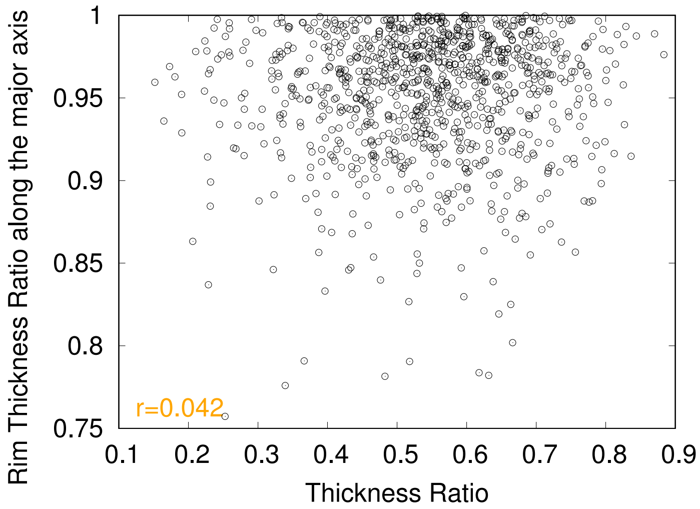

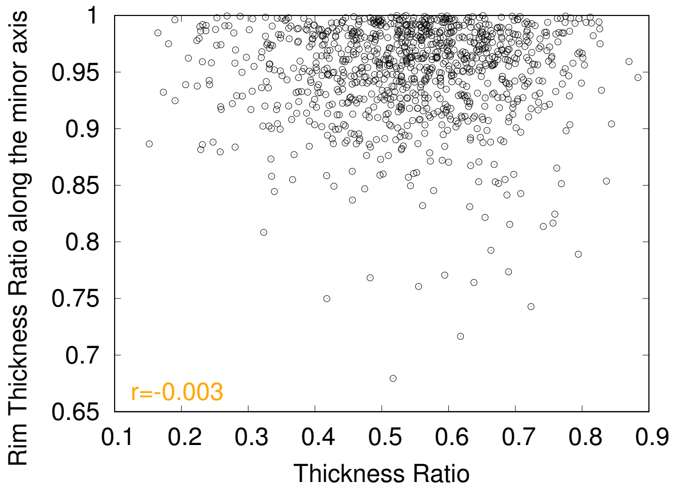

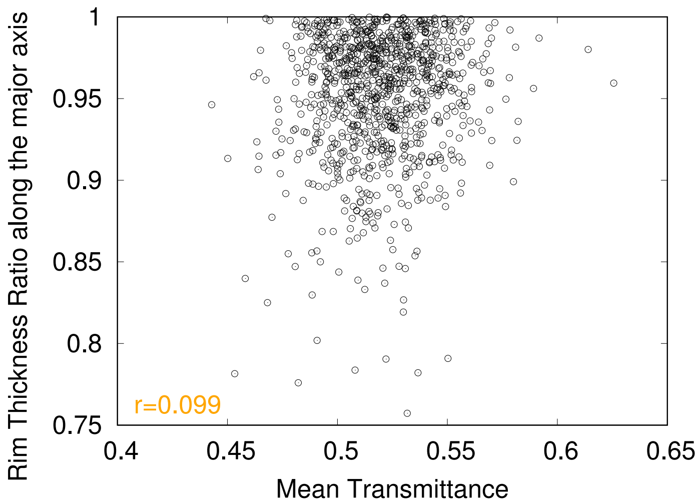

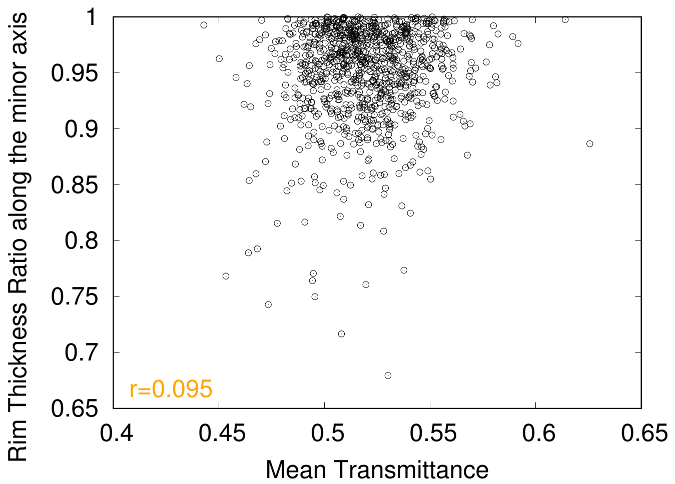

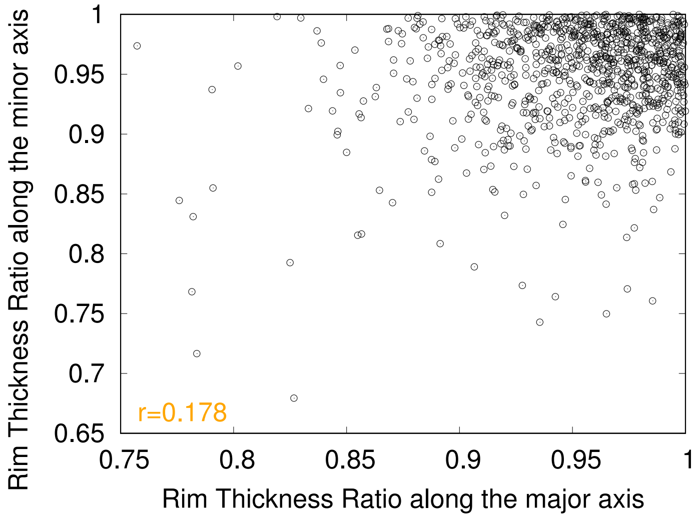

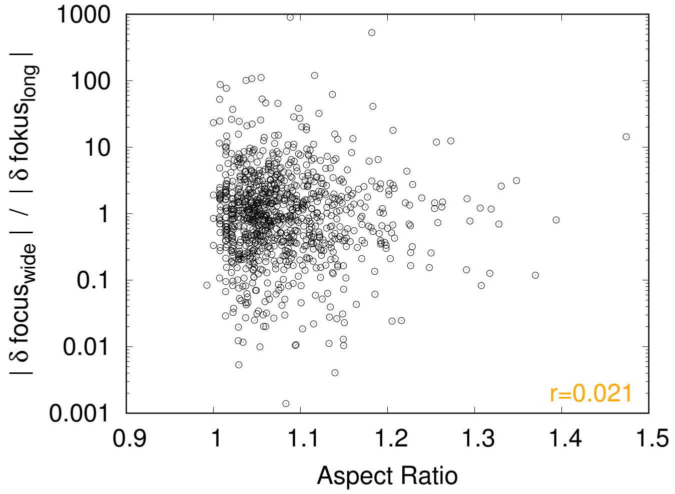

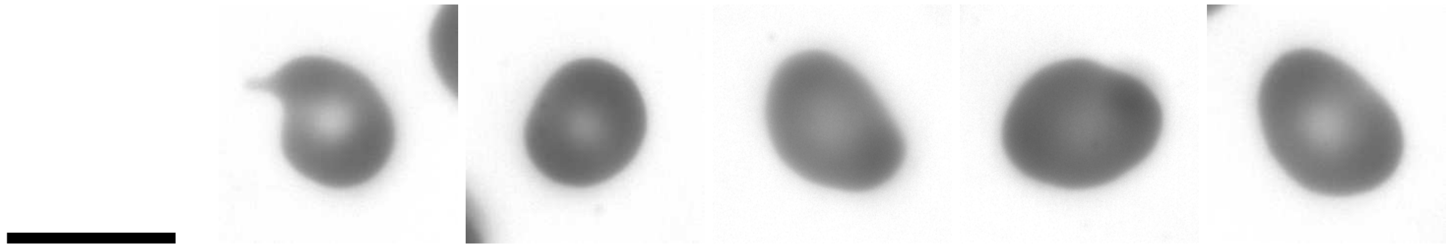

10 µm   0962 126   0692 ob   0323 044   0444 059   0735 098

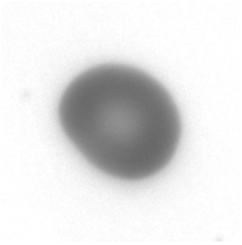

0841 110

Donor 4, serum washed

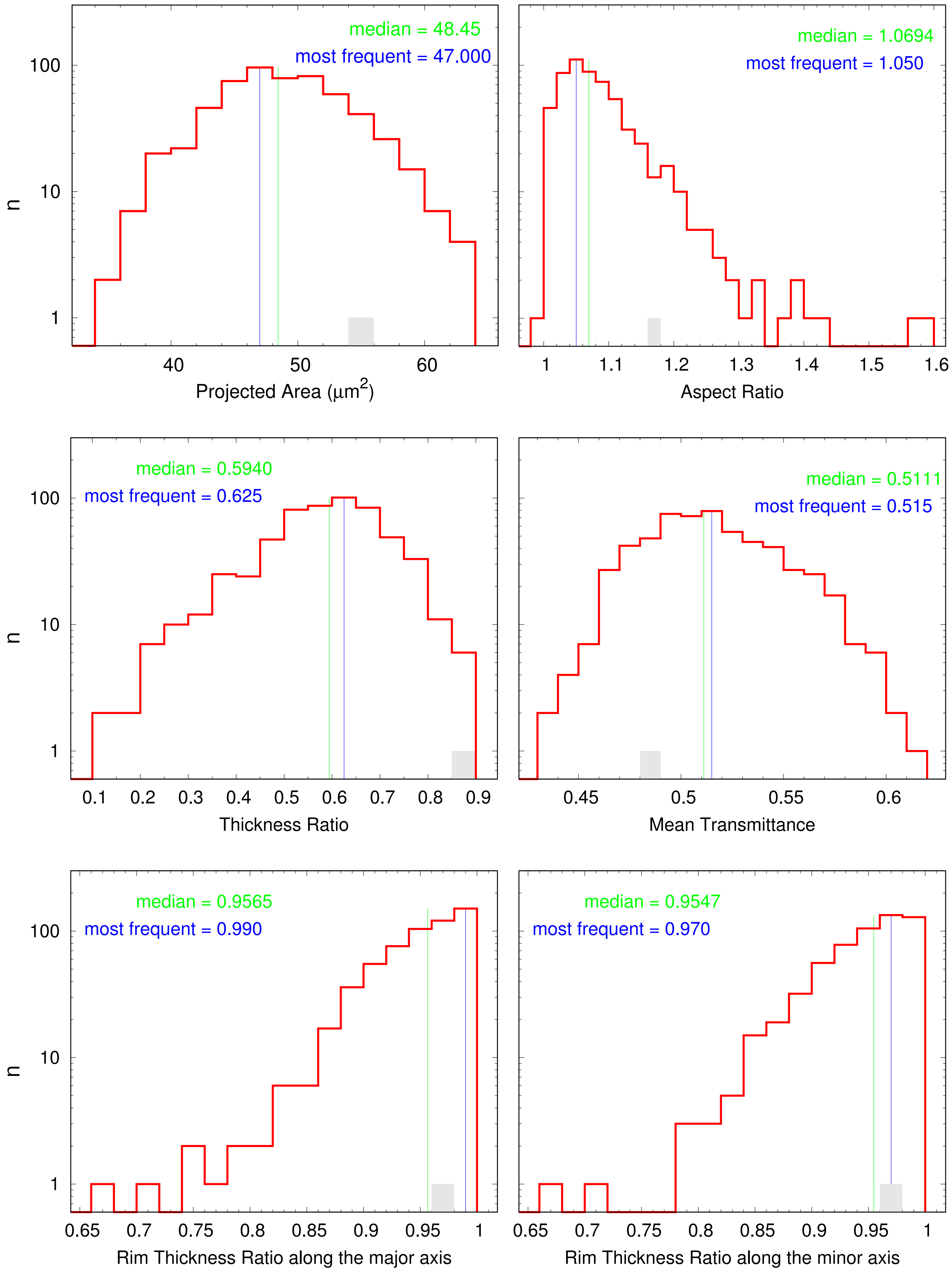

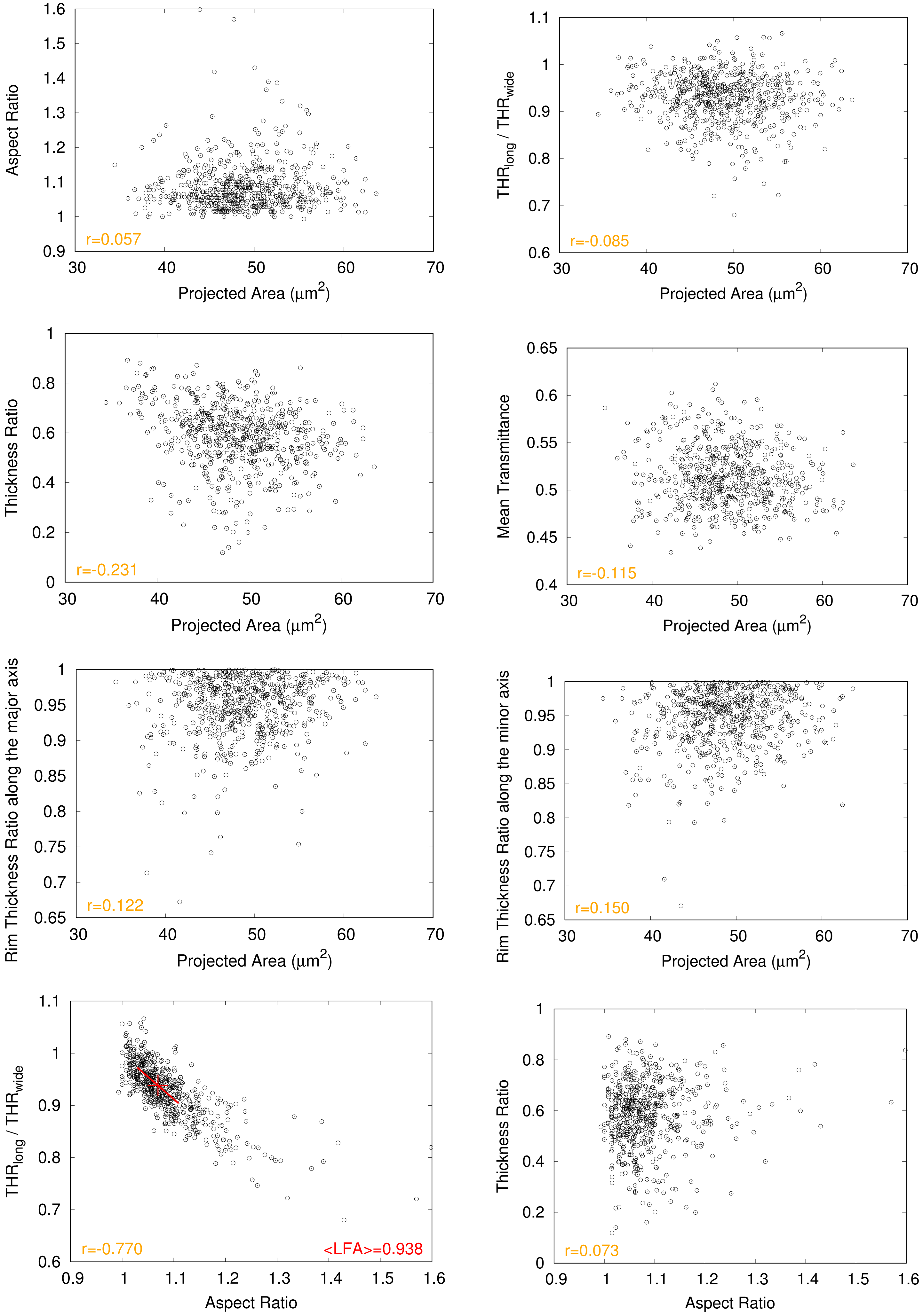
Aspect Ratio
Projected Area (μm2)
r=0.057
THRlong / THRwide
Projected Area (μm2)
r=-0.085
Thickness Ratio
Projected Area (μm2)
r=-0.231
Mean Transmittance
Projected Area (μm2)
r=-0.115
Rim Thickness Ratio along the major axis
Projected Area (μm2)
r=0.122
Rim Thickness Ratio along the minor axis
Projected Area (μm2)
r=0.150
THRlong / THRwide
Aspect Ratio
r=-0.770
<LFA>=0.938
Thickness Ratio
Aspect Ratio
r=0.073

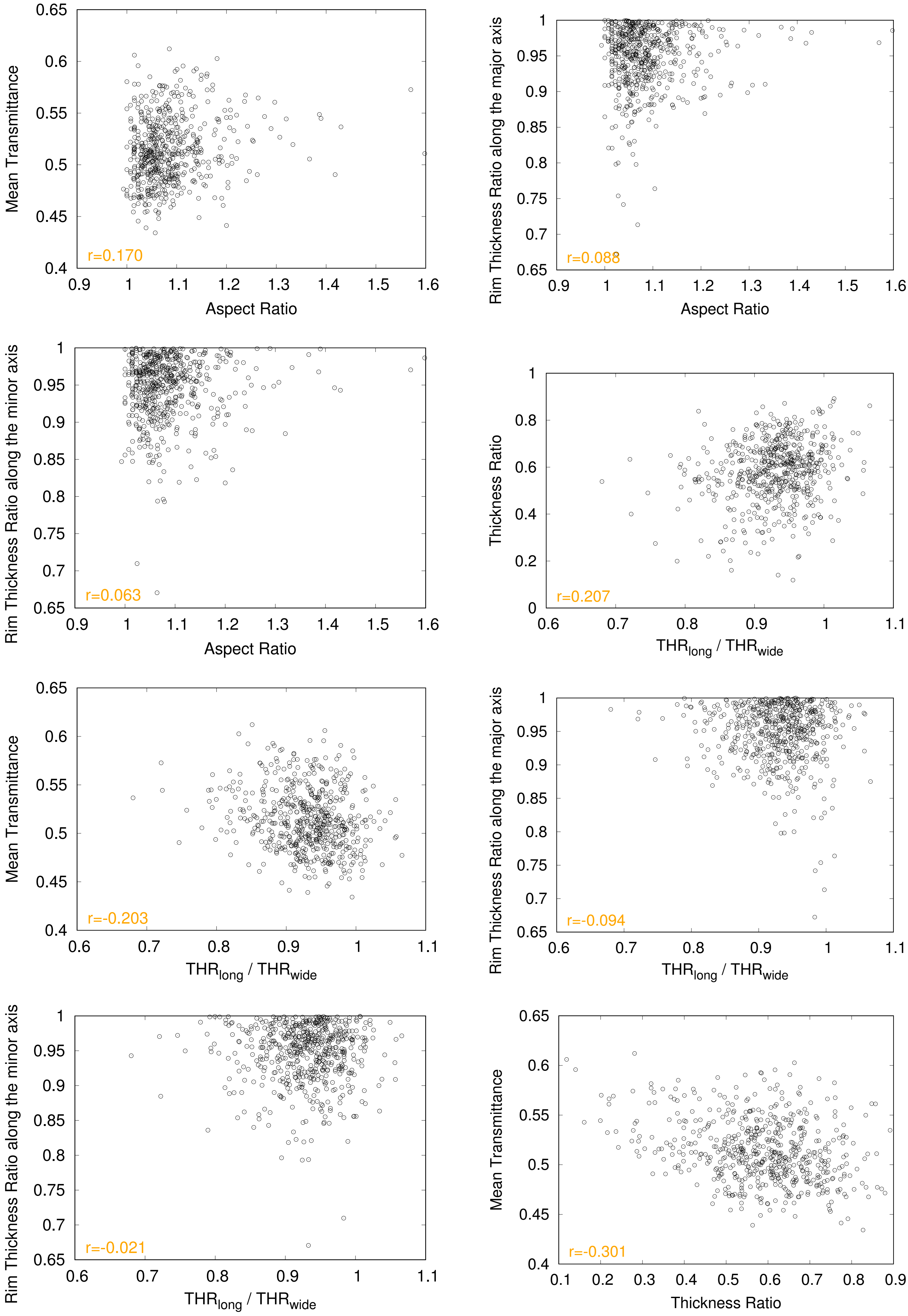
Mean Transmittance
Aspect Ratio
r=0.170
Rim Thickness Ratio along the major axis
Aspect Ratio
r=0.088
Rim Thickness Ratio along the minor axis
Aspect Ratio
r=0.063
Thickness Ratio
THR_{long} / THR_{wide}
r=0.207
Mean Transmittance
THR_{long} / THR_{wide}
r=-0.203
Rim Thickness Ratio along the major axis
THR_{long} / THR_{wide}
r=-0.094
Rim Thickness Ratio along the minor axis
THR_{long} / THR_{wide}
r=-0.021
Mean Transmittance
Thickness Ratio
r=-0.301

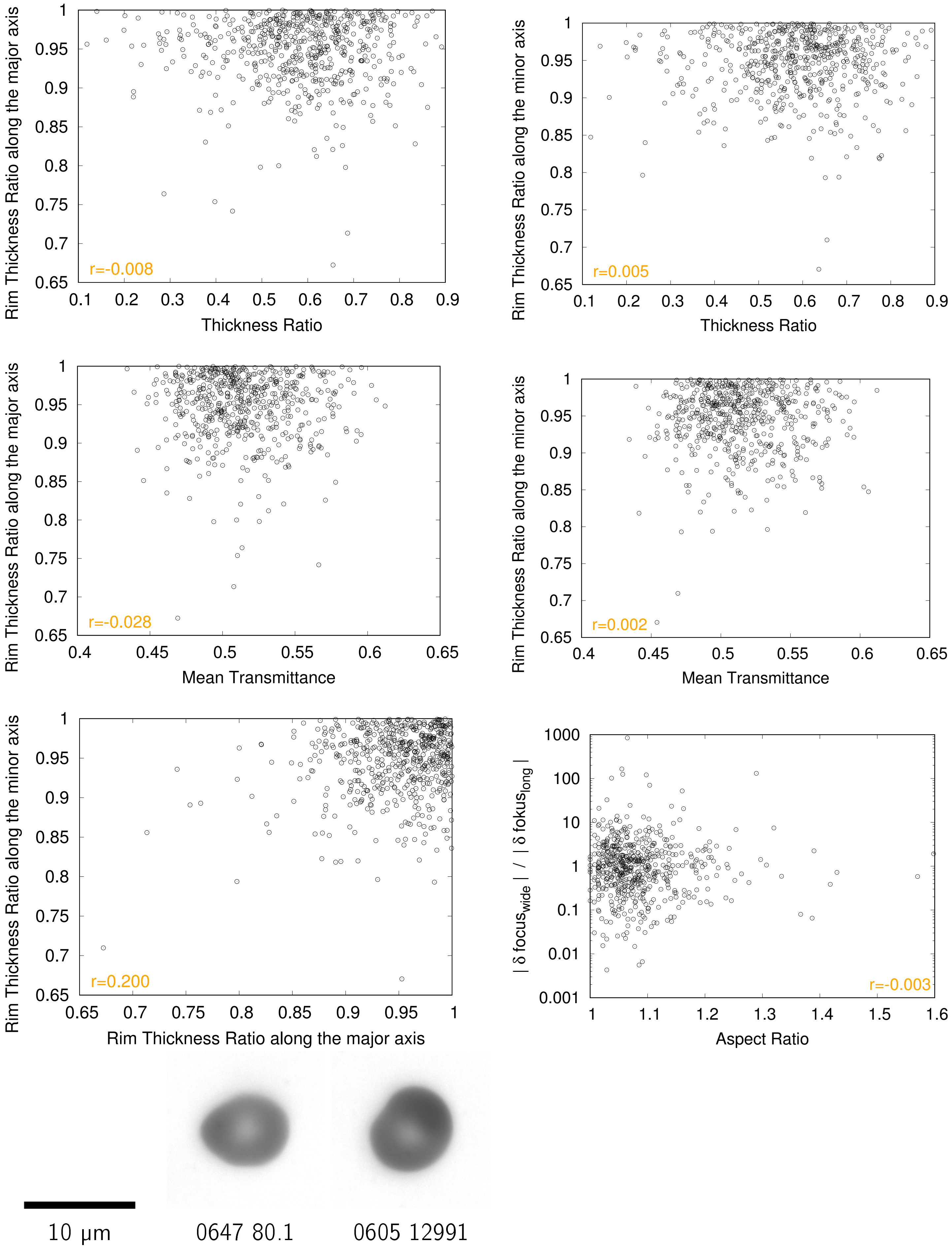
Rim Thickness Ratio along the major axis
Thickness Ratio
r=-0.008
Rim Thickness Ratio along the minor axis
Thickness Ratio
r=0.005
Rim Thickness Ratio along the major axis
Mean Transmittance
r=-0.028
Rim Thickness Ratio along the minor axis
Mean Transmittance
r=0.002
Rim Thickness Ratio along the minor axis
Rim Thickness Ratio along the major axis
r=0.200
| δ focus_wide | / | δ fokus_long |
Aspect Ratio
r=-0.003
10 µm
0647 80.1
0605 12991

Donor 5, plasma

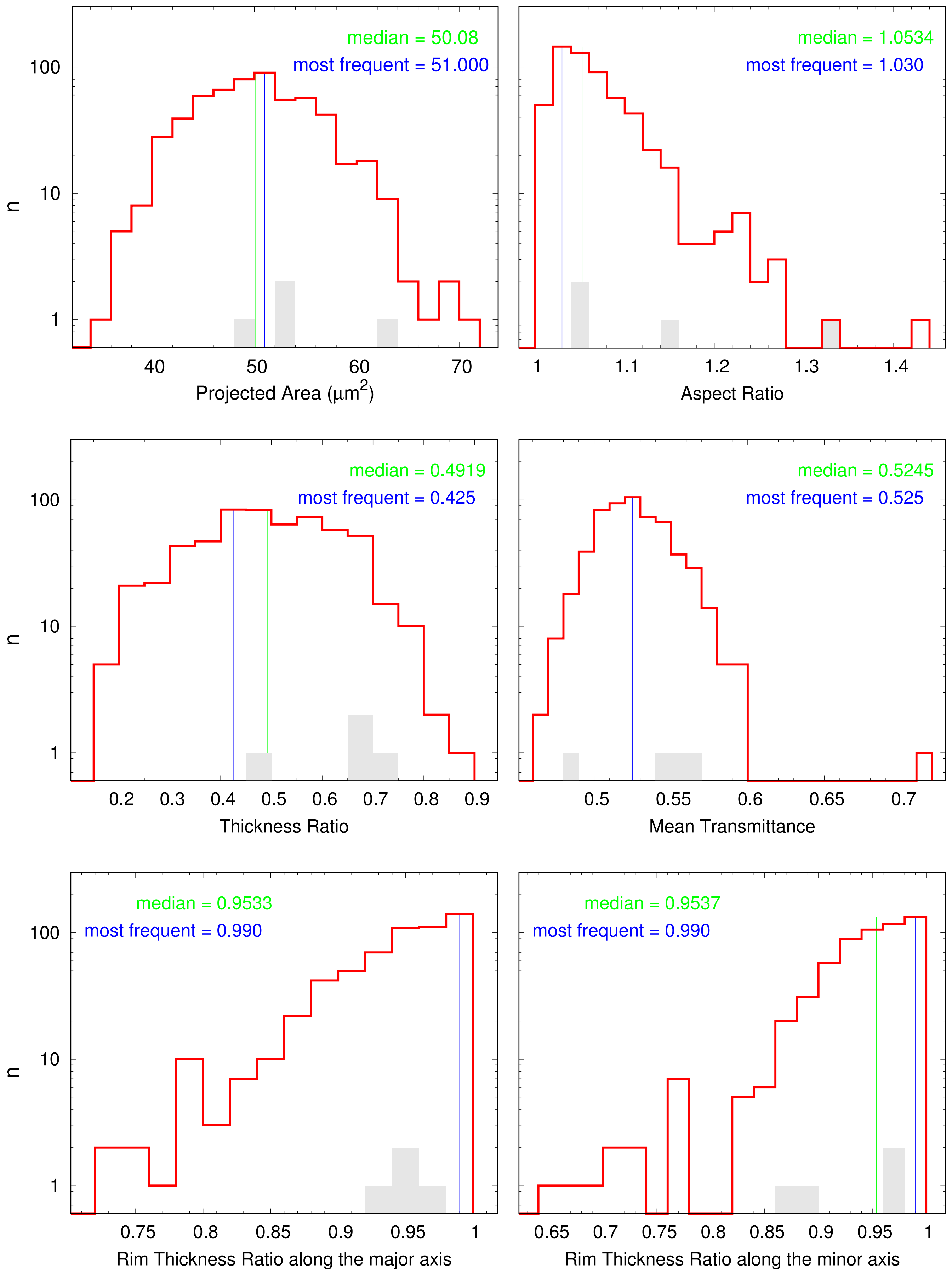

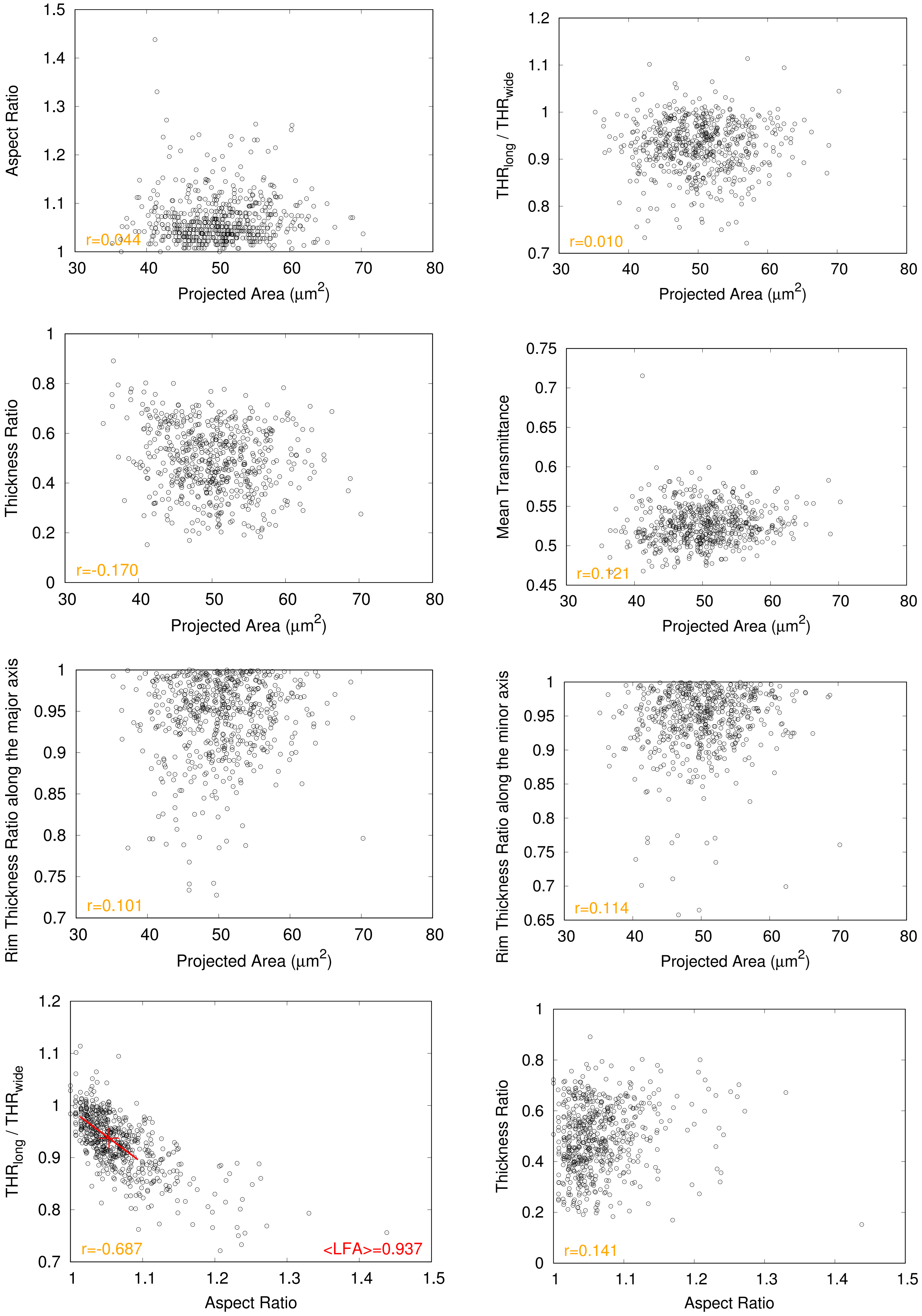
Aspect Ratio
Projected Area (μm2)
r=0.044
THRlong / THRwide
r=0.010
Thickness Ratio
r=-0.170
Mean Transmittance
r=0.121
Rim Thickness Ratio along the major axis
r=0.101
Rim Thickness Ratio along the minor axis
r=0.114
r=-0.687
<LFA>=0.937
r=0.141

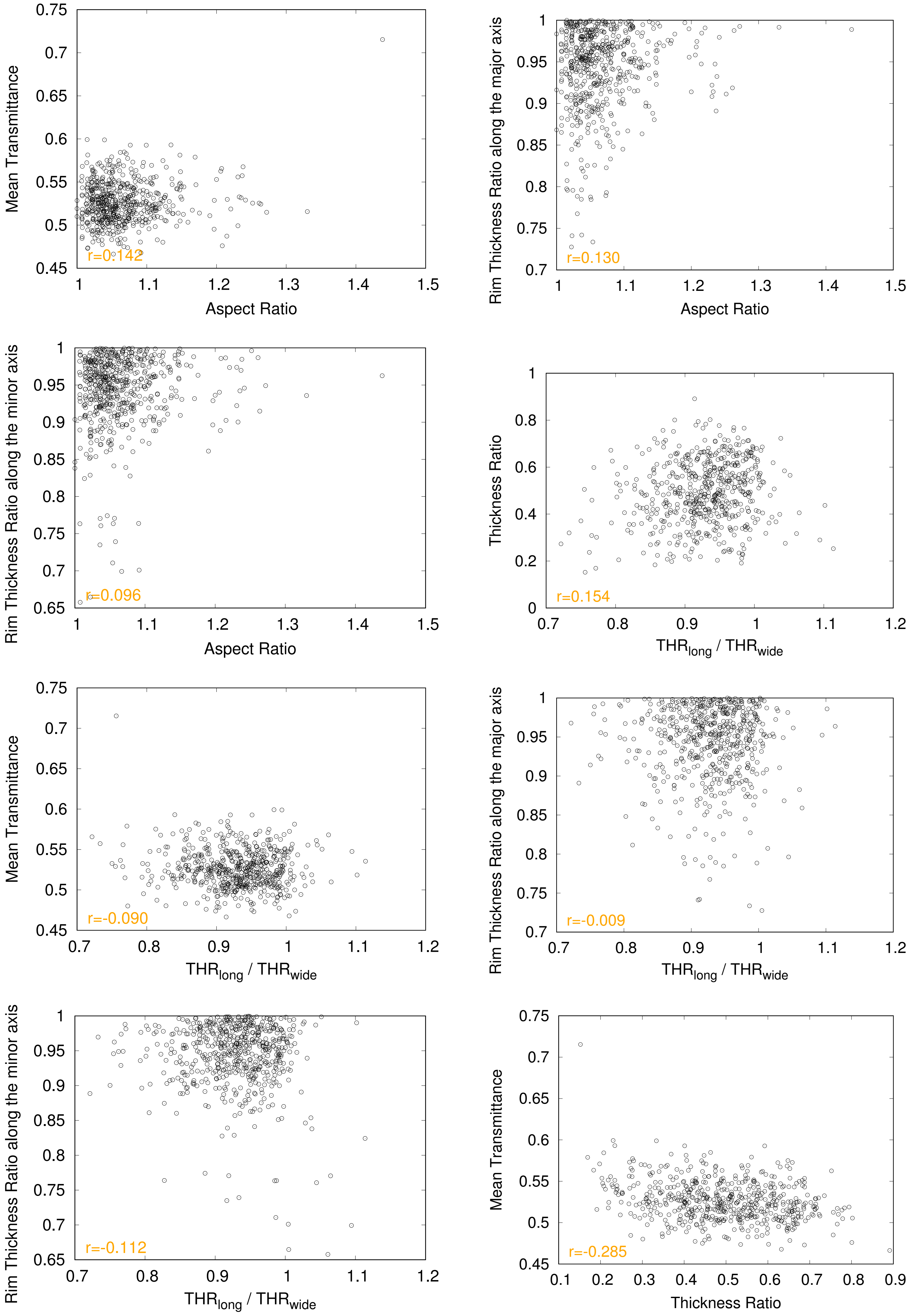
Mean Transmittance
Aspect Ratio
r=0.142
Rim Thickness Ratio along the major axis
Aspect Ratio
r=0.130
Rim Thickness Ratio along the minor axis
Aspect Ratio
r=0.096
Thickness Ratio
THR_{long} / THR_{wide}
r=0.154
Mean Transmittance
THR_{long} / THR_{wide}
r=-0.090
Rim Thickness Ratio along the major axis
THR_{long} / THR_{wide}
r=-0.009
Rim Thickness Ratio along the minor axis
THR_{long} / THR_{wide}
r=-0.112
Mean Transmittance
Thickness Ratio
r=-0.285

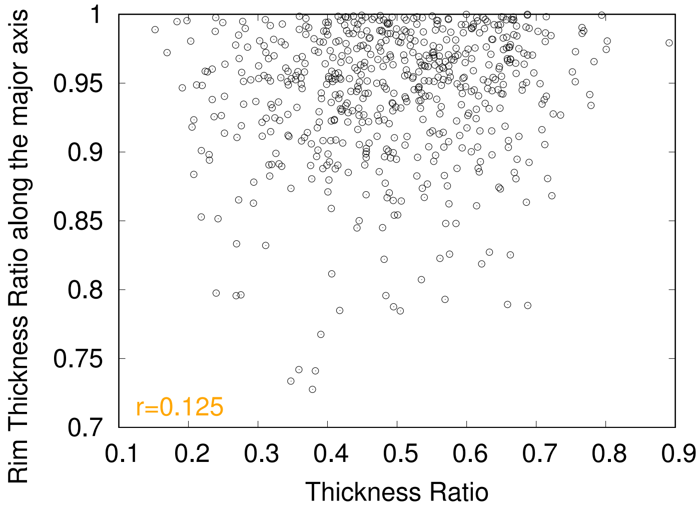
Rim Thickness Ratio along the major axis
Thickness Ratio
r=0.125

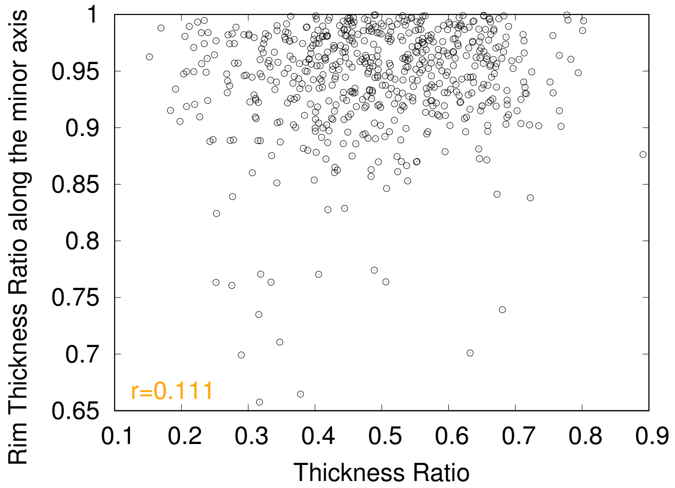
Rim Thickness Ratio along the minor axis
Thickness Ratio
r=0.111

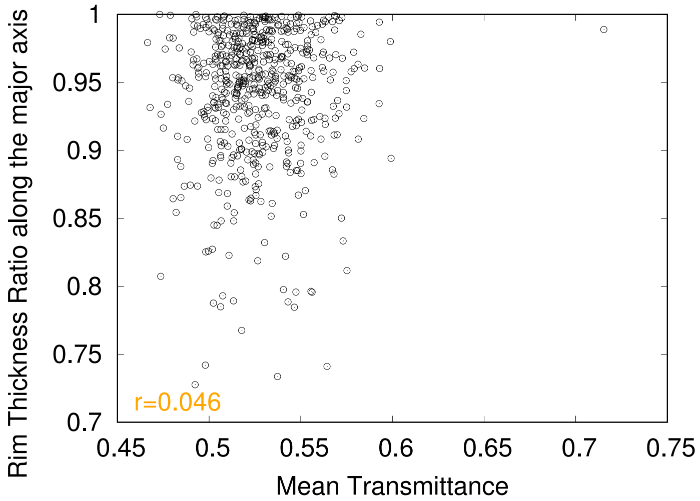
Rim Thickness Ratio along the major axis
Mean Transmittance
r=0.046

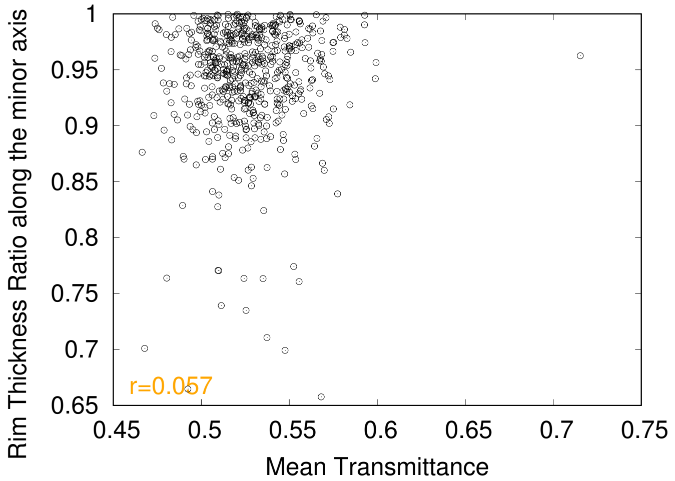
Rim Thickness Ratio along the minor axis
Mean Transmittance
r=0.057

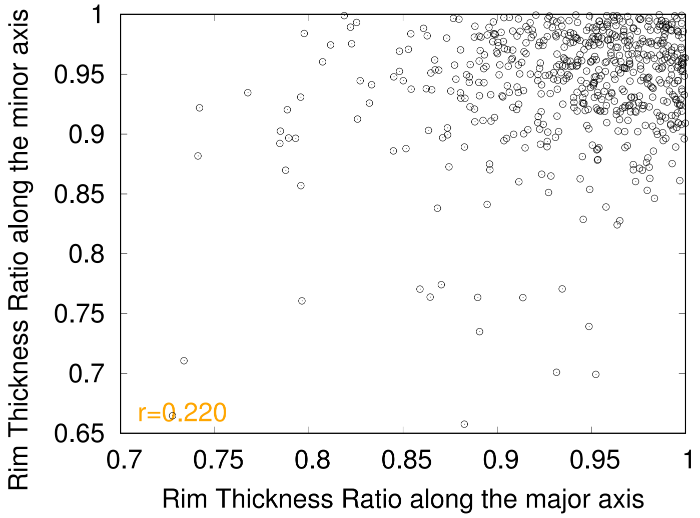
Rim Thickness Ratio along the minor axis
Rim Thickness Ratio along the major axis
r=0.220

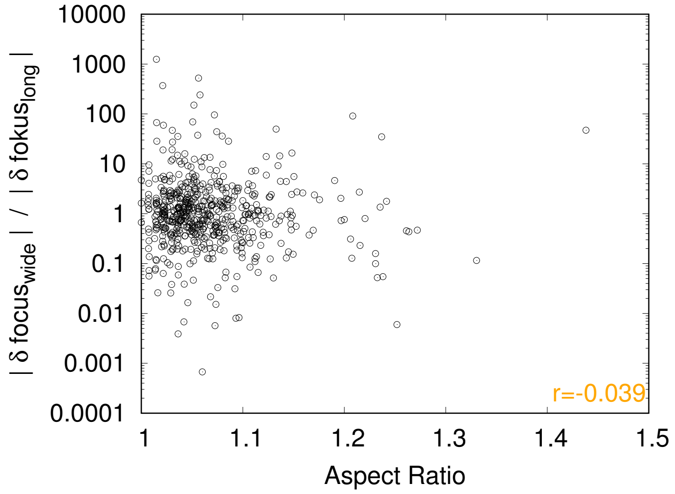
| δ focus_wide | / | δ fokus_long |
Aspect Ratio
r=-0.039

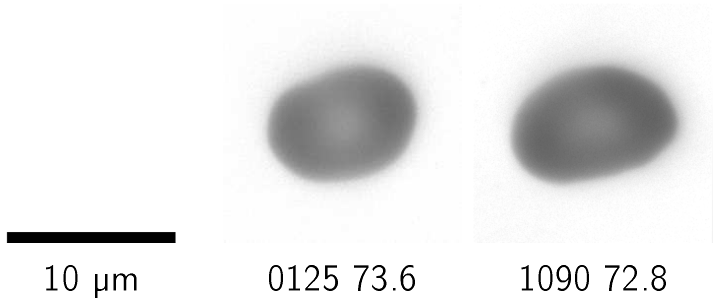
10 μm
0125 73.6
1090 72.8

Donor 5, serum washed

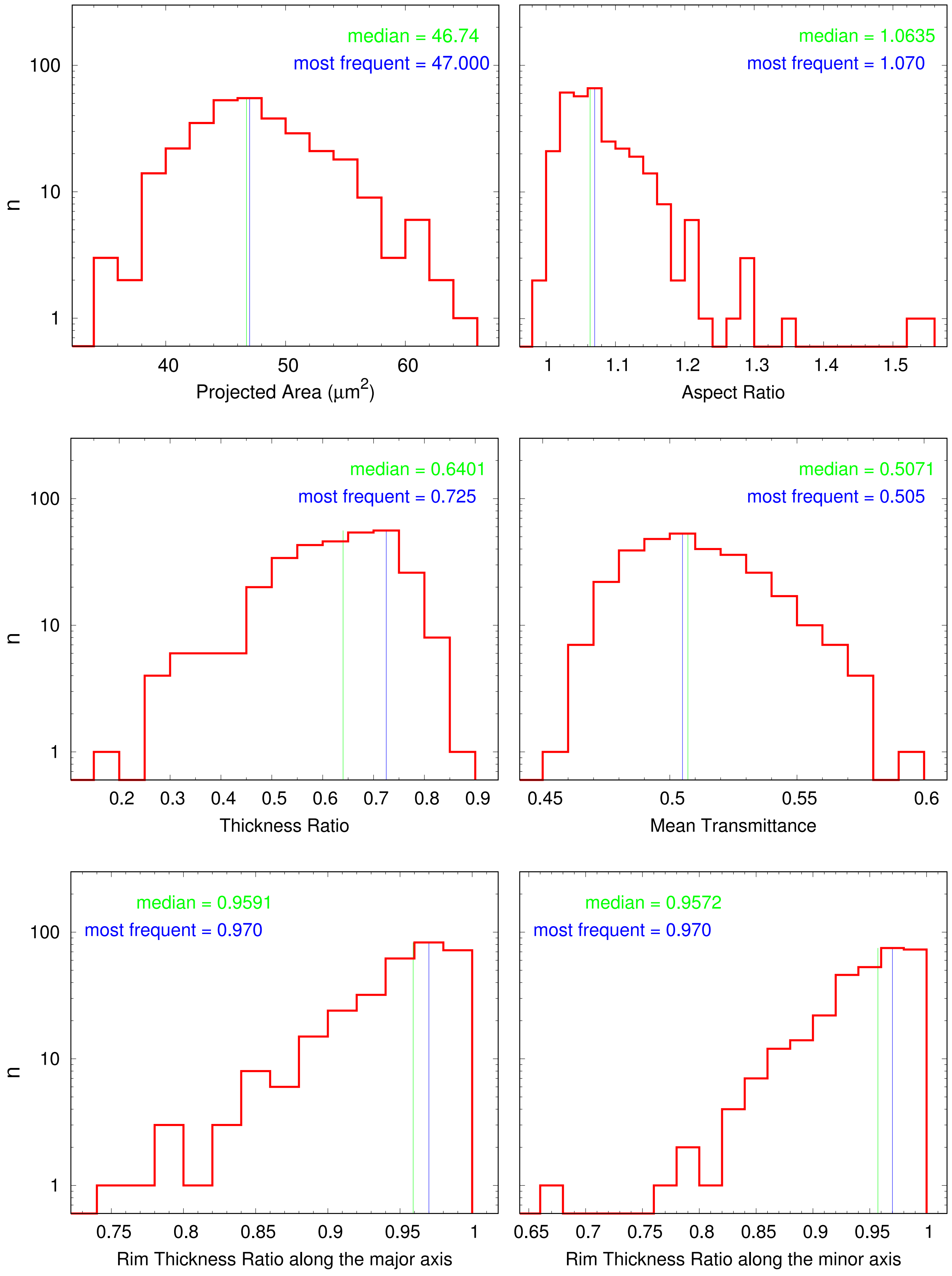

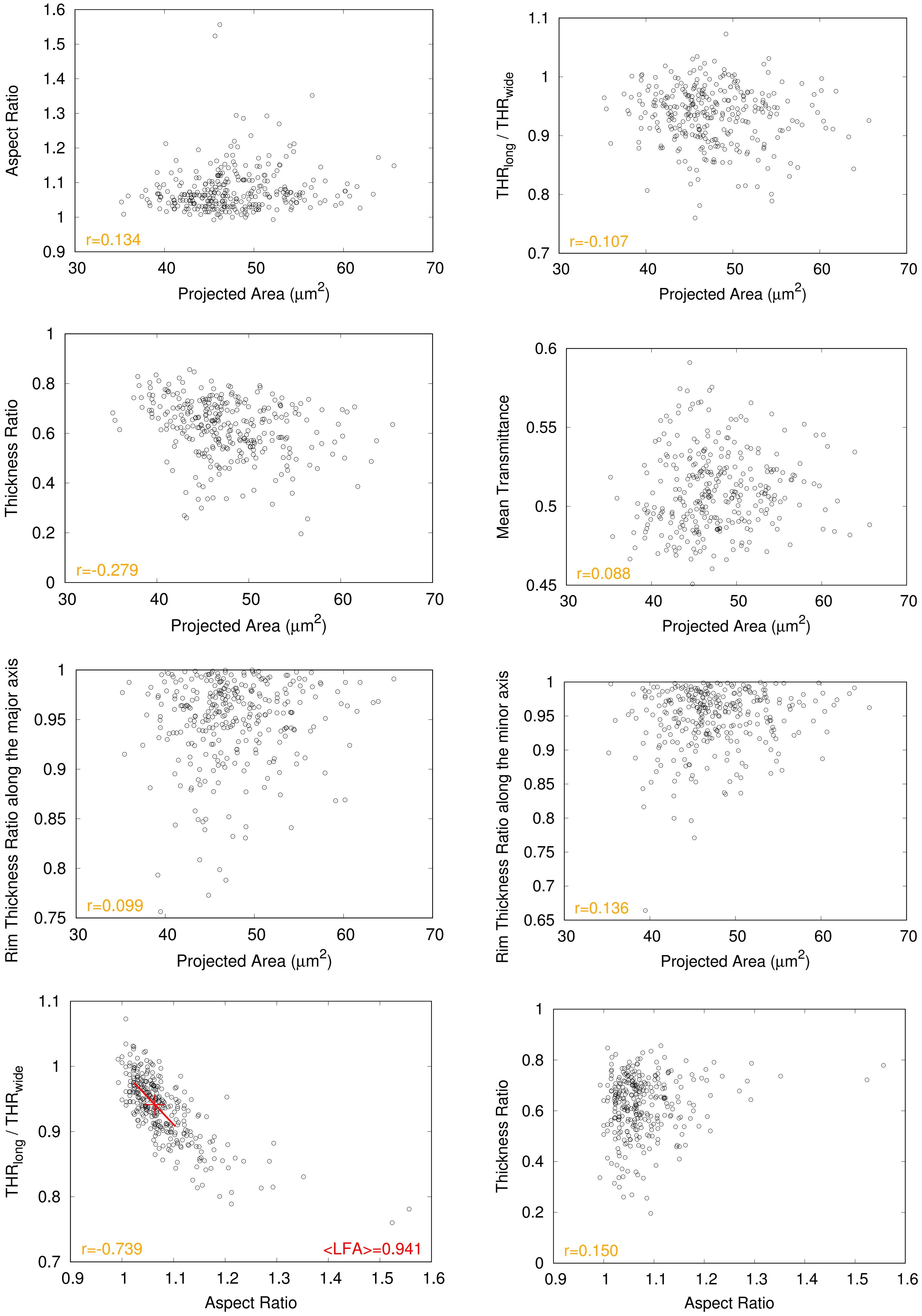

Aspect Ratio
Projected Area (μm2)
r=0.134
THRlong / THRwide
Projected Area (μm2)
r=-0.107
Thickness Ratio
Projected Area (μm2)
r=-0.279
Mean Transmittance
Projected Area (μm2)
r=0.088
Rim Thickness Ratio along the major axis
Projected Area (μm2)
r=0.099
Rim Thickness Ratio along the minor axis
Projected Area (μm2)
r=0.136
THRlong / THRwide
Aspect Ratio
r=-0.739
<LFA>=0.941
Thickness Ratio
Aspect Ratio
r=0.150

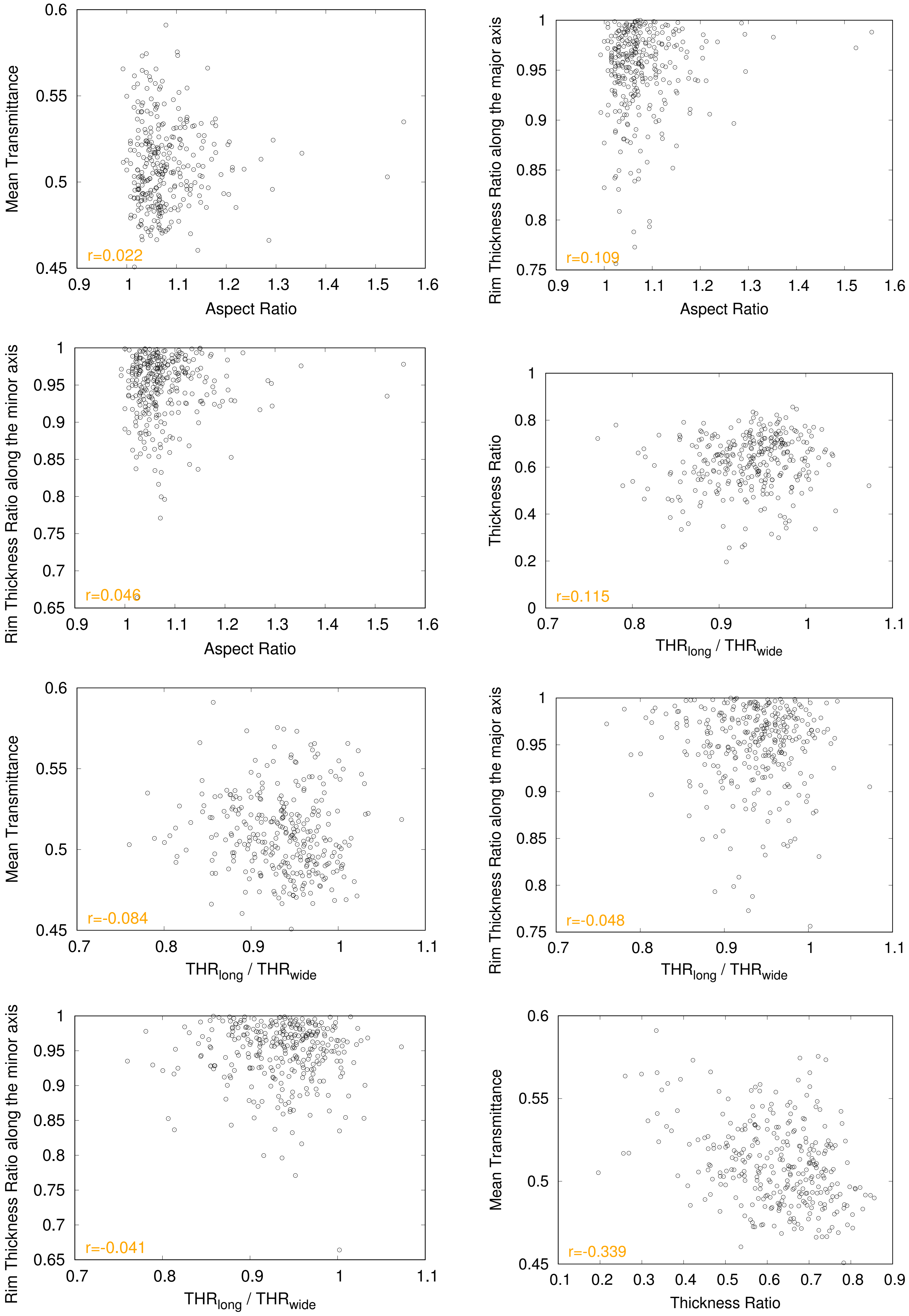
Mean Transmittance
Aspect Ratio
r=0.022
Rim Thickness Ratio along the major axis
Aspect Ratio
r=0.109
Rim Thickness Ratio along the minor axis
Aspect Ratio
r=0.046
Thickness Ratio
THR_{long} / THR_{wide}
r=0.115
Mean Transmittance
THR_{long} / THR_{wide}
r=-0.084
Rim Thickness Ratio along the major axis
THR_{long} / THR_{wide}
r=-0.048
Rim Thickness Ratio along the minor axis
THR_{long} / THR_{wide}
r=-0.041
Mean Transmittance
Thickness Ratio
r=-0.339

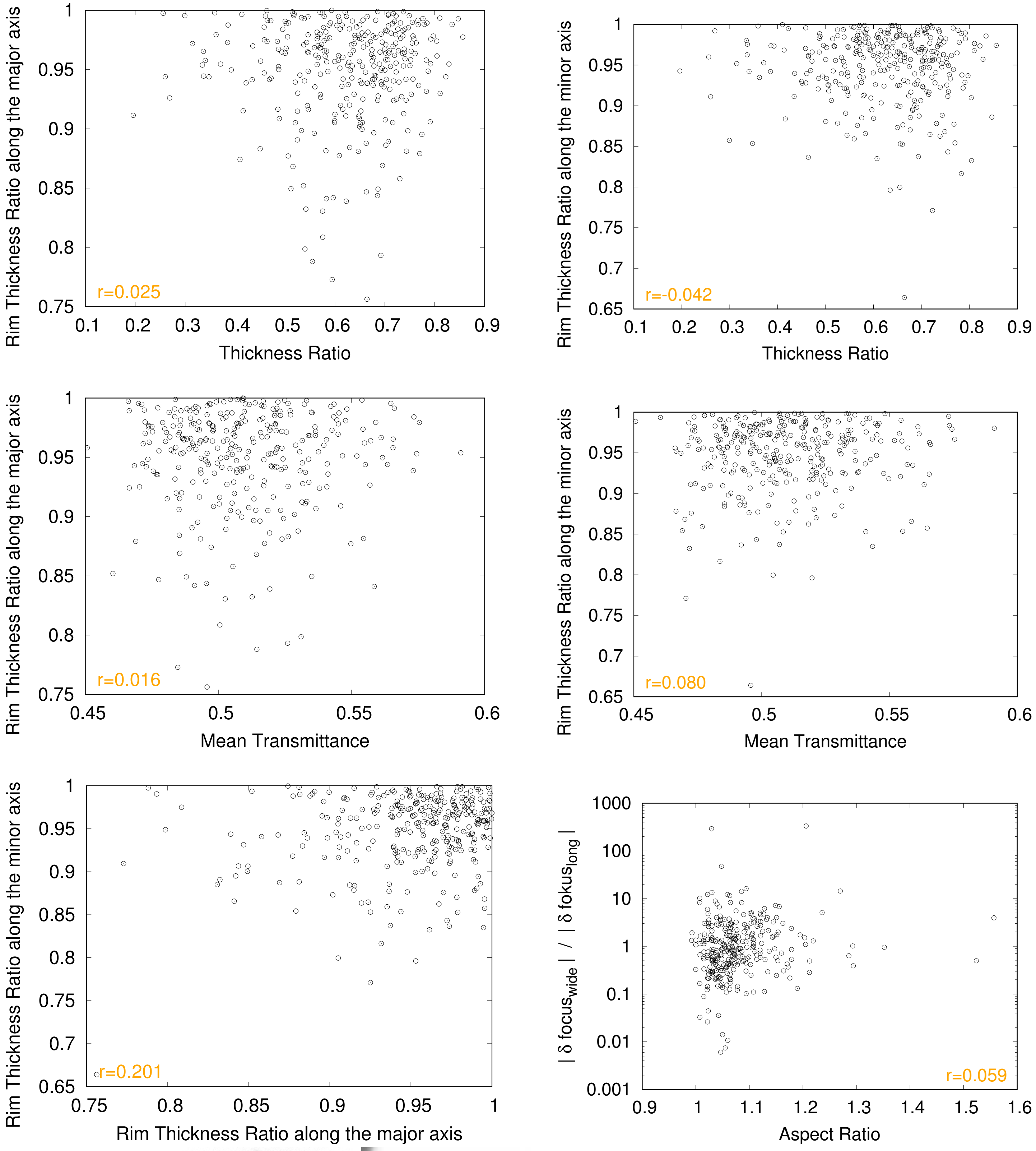

Rim Thickness Ratio along the major axis
Thickness Ratio
r=0.025
Rim Thickness Ratio along the minor axis
Thickness Ratio
r=-0.042
Rim Thickness Ratio along the major axis
Mean Transmittance
r=0.016
Rim Thickness Ratio along the minor axis
Mean Transmittance
r=0.080
Rim Thickness Ratio along the minor axis
Rim Thickness Ratio along the major axis
r=0.201
| δ focus$_{wide}$ | / | δ fokus$_{long}$ |
Aspect Ratio
r=0.059

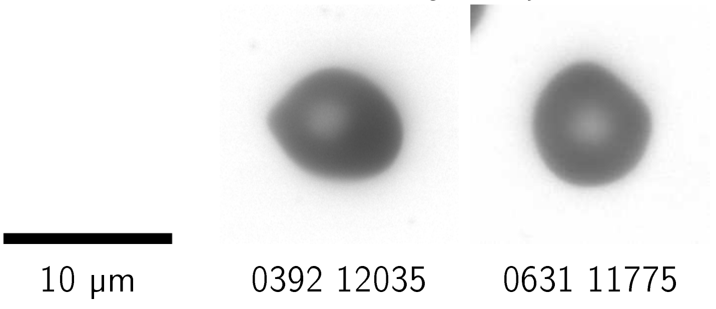

10 μm
0392 12035
0631 11775

Donor 6, plasma

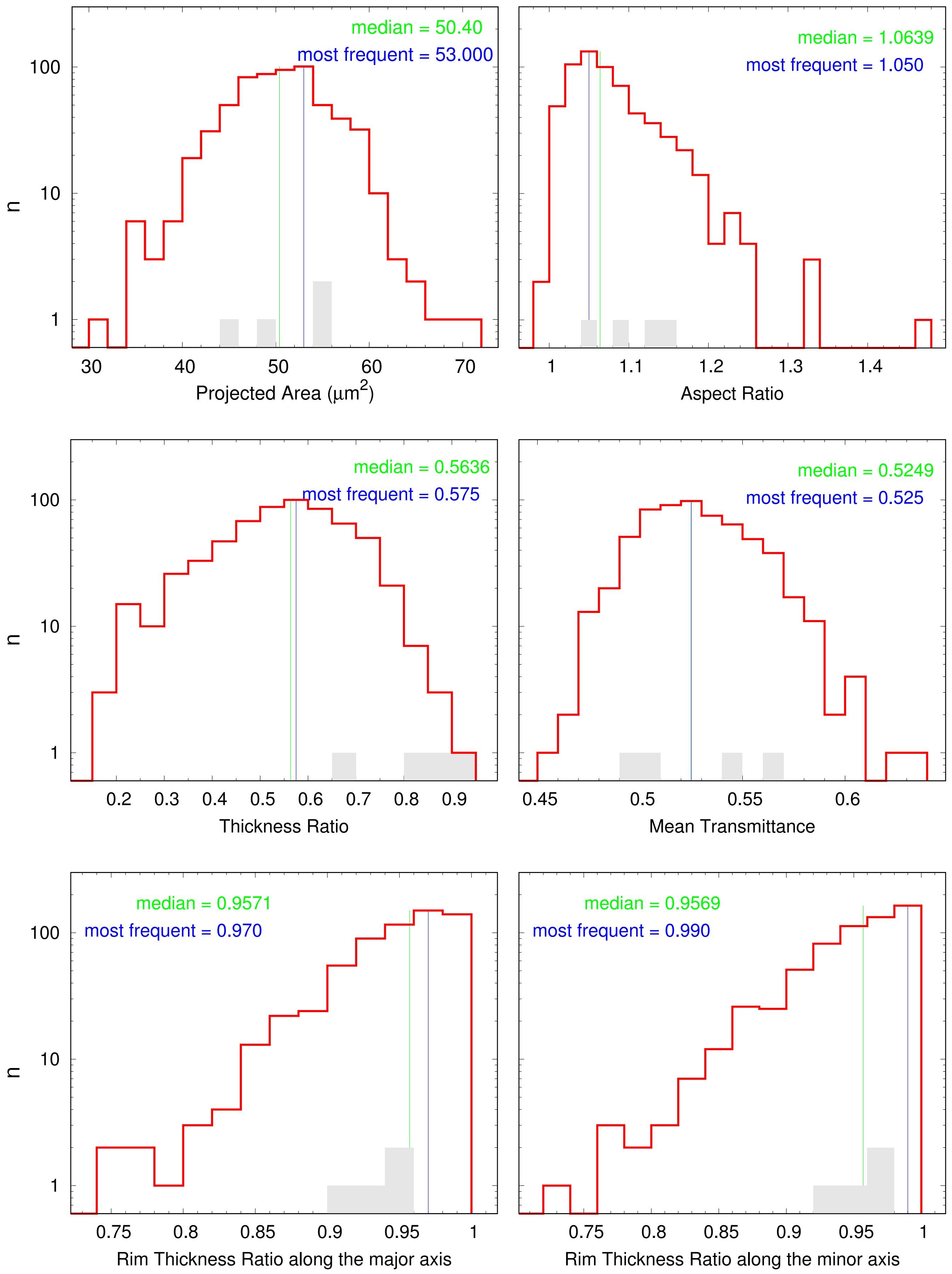

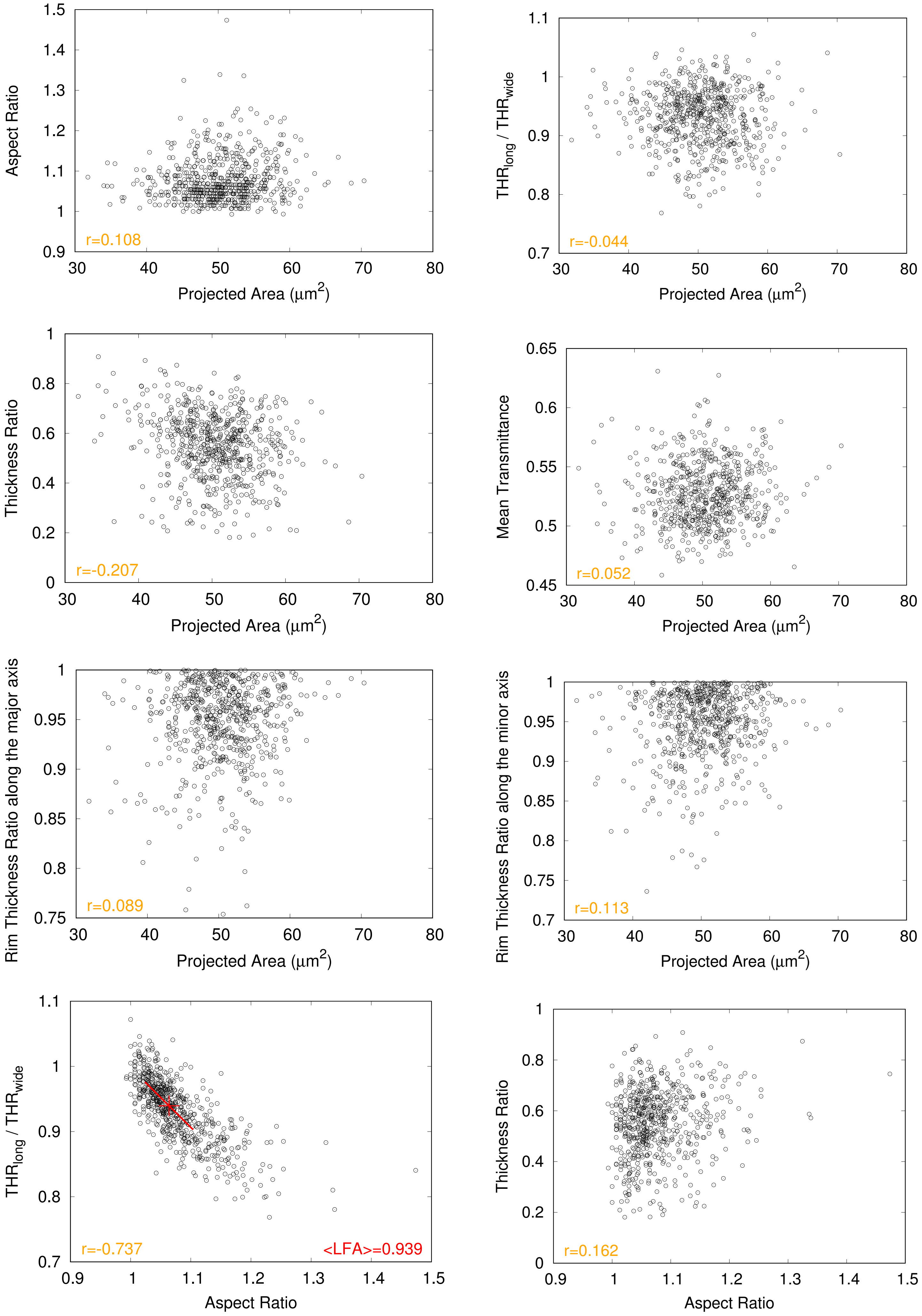
Aspect Ratio
Projected Area (μm2)
r=0.108
THRlong / THRwide
Projected Area (μm2)
r=-0.044
Thickness Ratio
Projected Area (μm2)
r=-0.207
Mean Transmittance
Projected Area (μm2)
r=0.052
Rim Thickness Ratio along the major axis
Projected Area (μm2)
r=0.089
Rim Thickness Ratio along the minor axis
Projected Area (μm2)
r=0.113
THRlong / THRwide
Aspect Ratio
r=-0.737
<LFA>=0.939
Thickness Ratio
Aspect Ratio
r=0.162

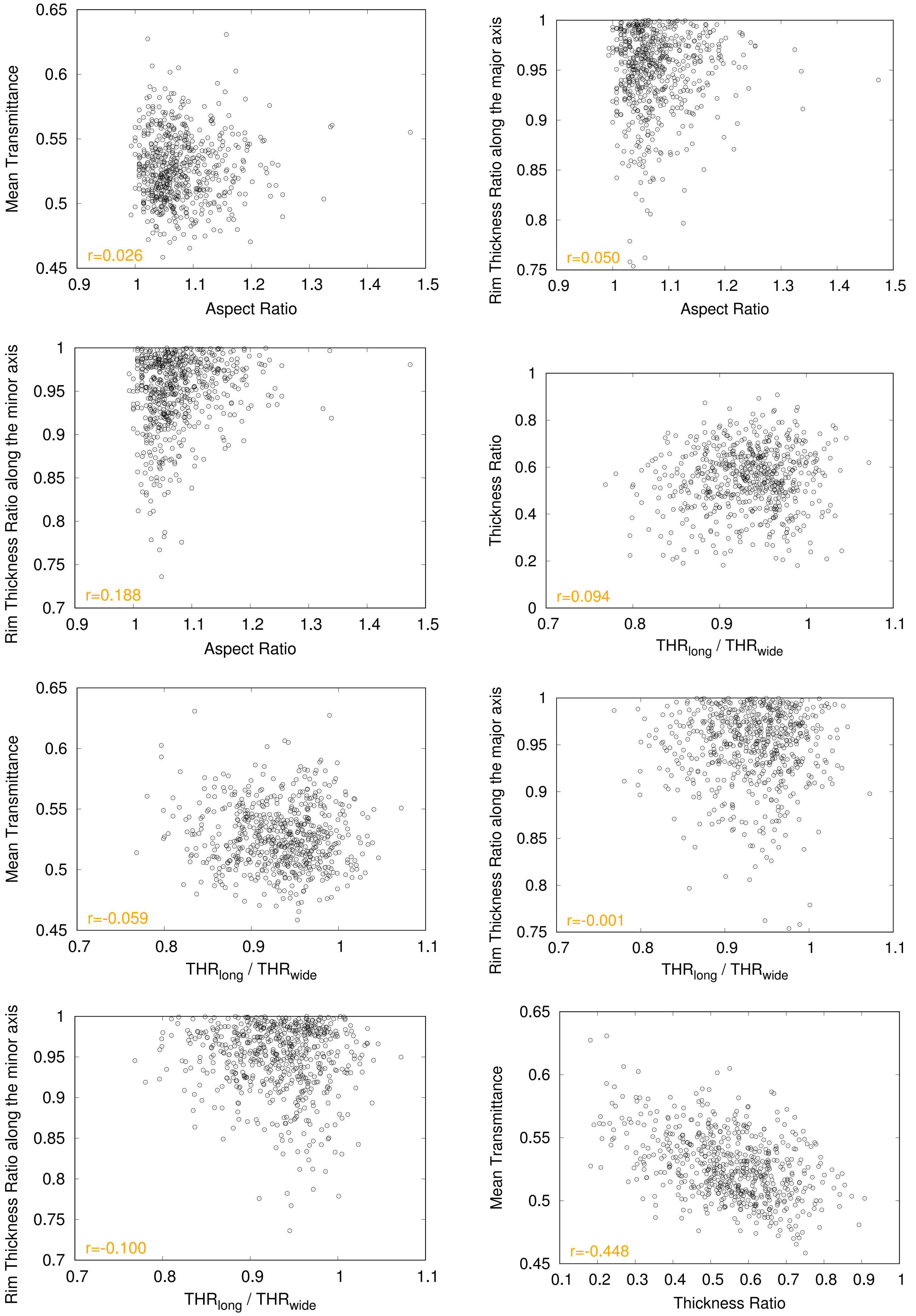

Mean Transmittance
Aspect Ratio
r=0.026
Rim Thickness Ratio along the major axis
Aspect Ratio
r=0.050
Rim Thickness Ratio along the minor axis
Aspect Ratio
r=0.188
Thickness Ratio
THR_{long} / THR_{wide}
r=0.094
Mean Transmittance
THR_{long} / THR_{wide}
r=-0.059
Rim Thickness Ratio along the major axis
THR_{long} / THR_{wide}
r=-0.001
Rim Thickness Ratio along the minor axis
THR_{long} / THR_{wide}
r=-0.100
Mean Transmittance
Thickness Ratio
r=-0.448

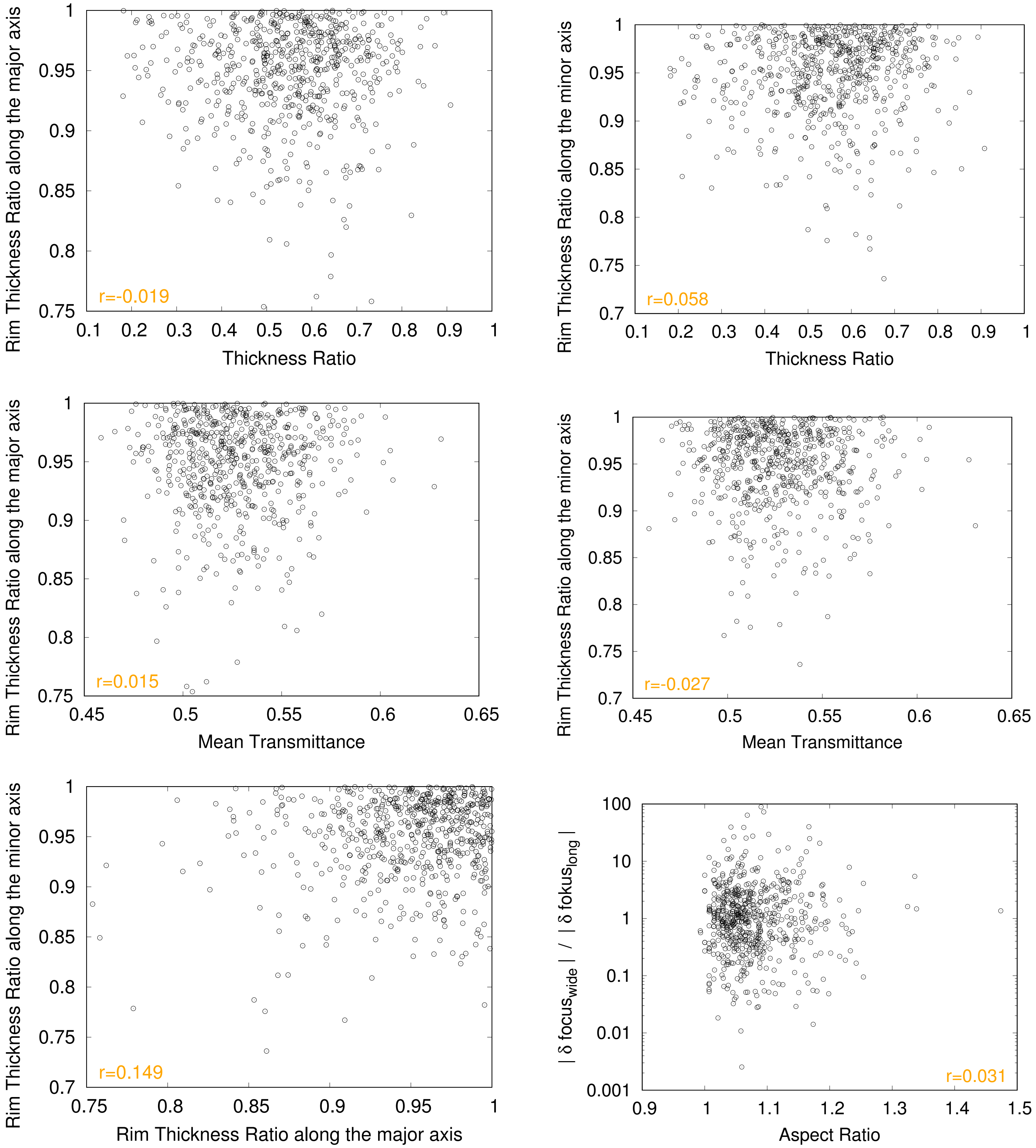

Rim Thickness Ratio along the major axis
Thickness Ratio
r=-0.019
Rim Thickness Ratio along the minor axis
Thickness Ratio
r=0.058
Rim Thickness Ratio along the major axis
Mean Transmittance
r=0.015
Rim Thickness Ratio along the minor axis
Mean Transmittance
r=-0.027
Rim Thickness Ratio along the minor axis
Rim Thickness Ratio along the major axis
r=0.149
| δ focus$_{wide}$ | / | δ fokus$_{long}$ |
Aspect Ratio
r=0.031

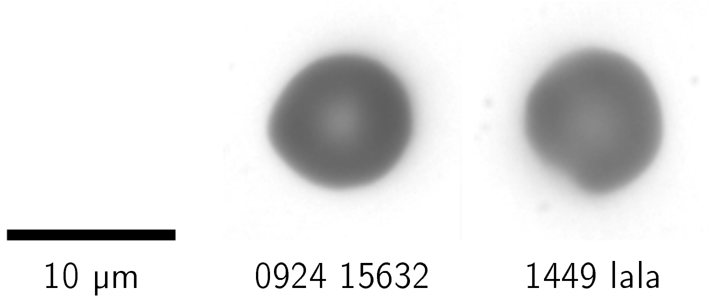

10 μm
0924 15632
1449 lala

Donor 6, serum washed

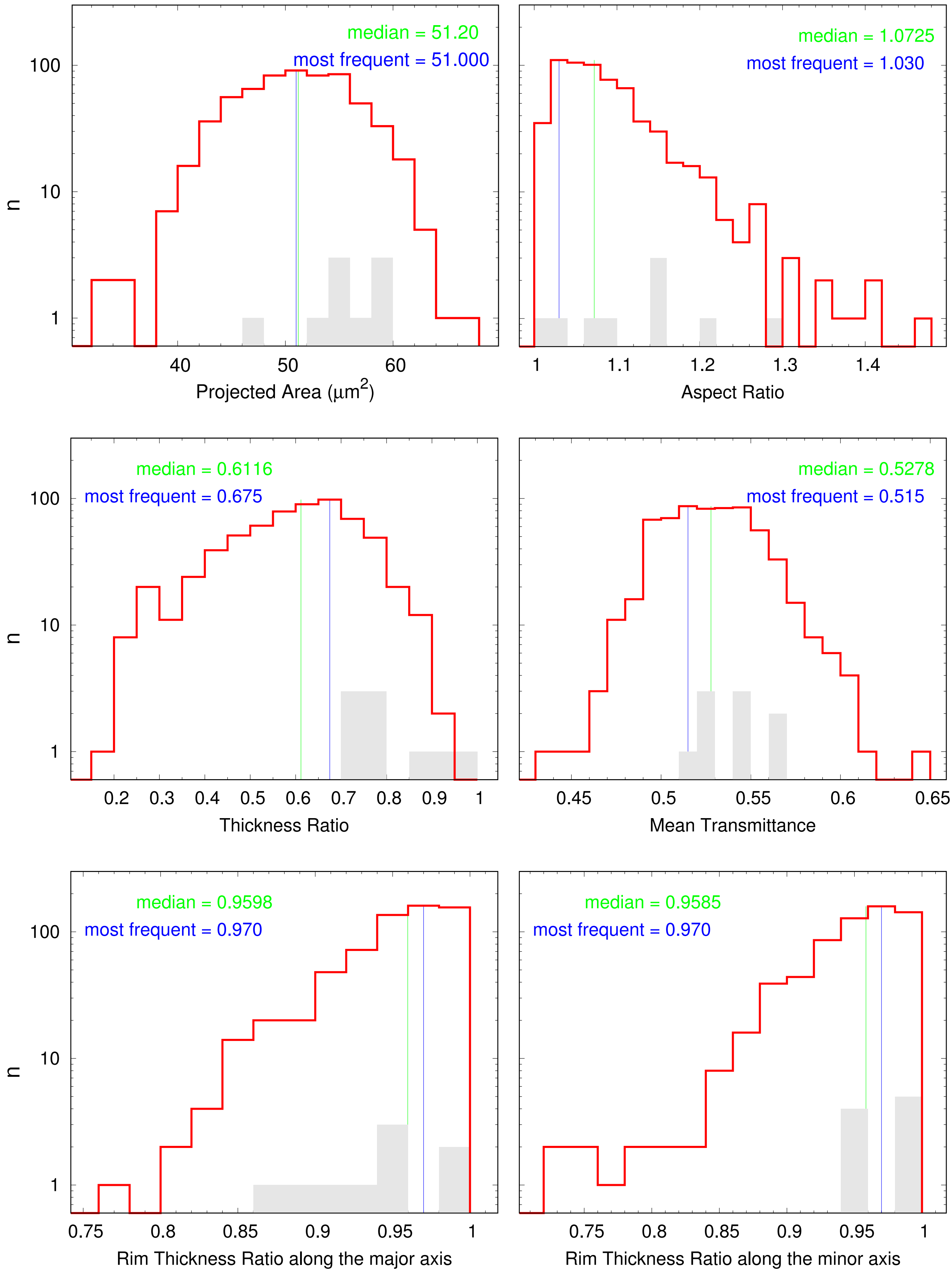

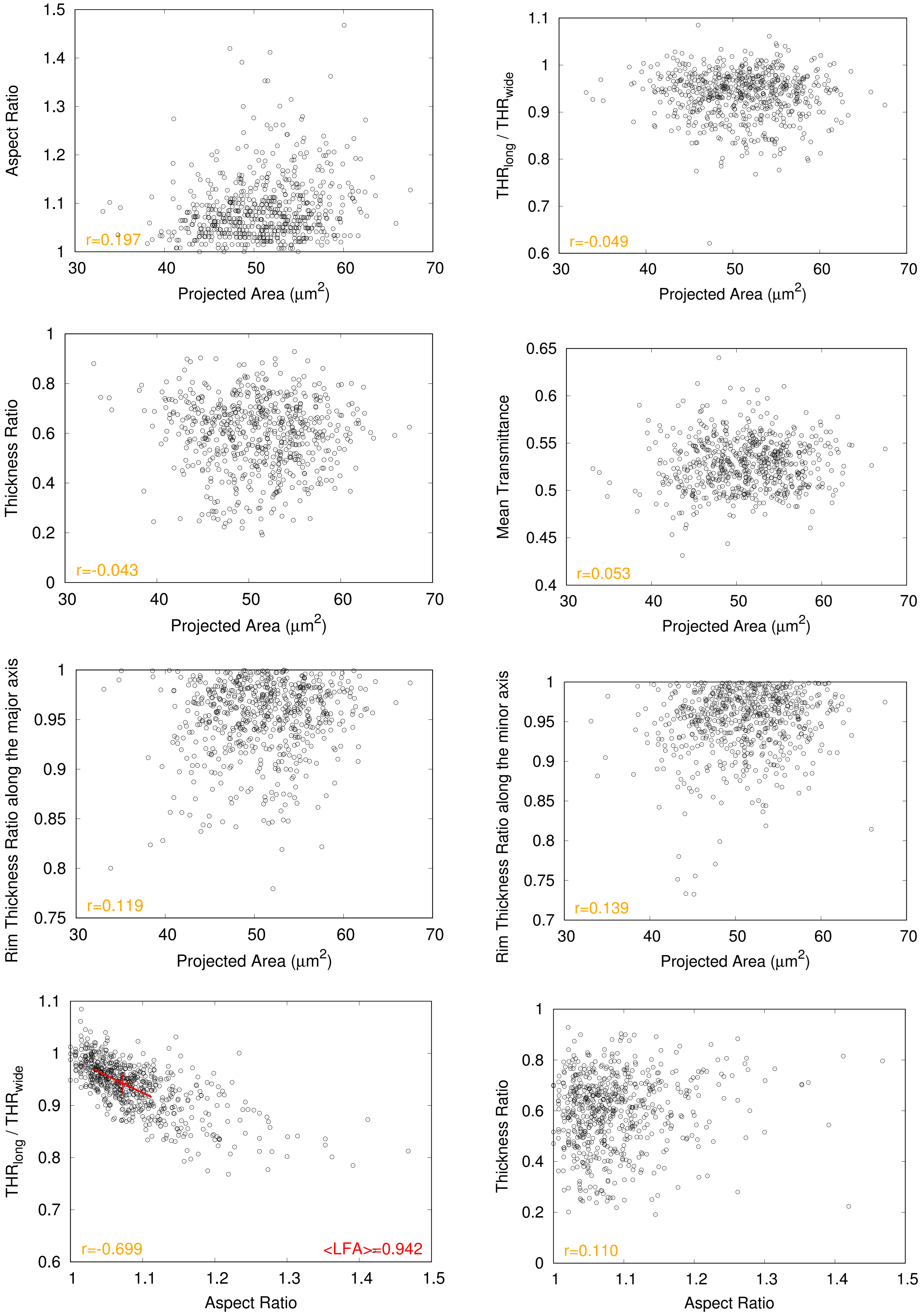

Aspect Ratio
Projected Area (μm2)
r=0.197
THRlong / THRwide
Projected Area (μm2)
r=-0.049
Thickness Ratio
Projected Area (μm2)
r=-0.043
Mean Transmittance
Projected Area (μm2)
r=0.053
Rim Thickness Ratio along the major axis
Projected Area (μm2)
r=0.119
Rim Thickness Ratio along the minor axis
Projected Area (μm2)
r=0.139
THRlong / THRwide
Aspect Ratio
r=-0.699
<LFA>=0.942
Thickness Ratio
Aspect Ratio
r=0.110

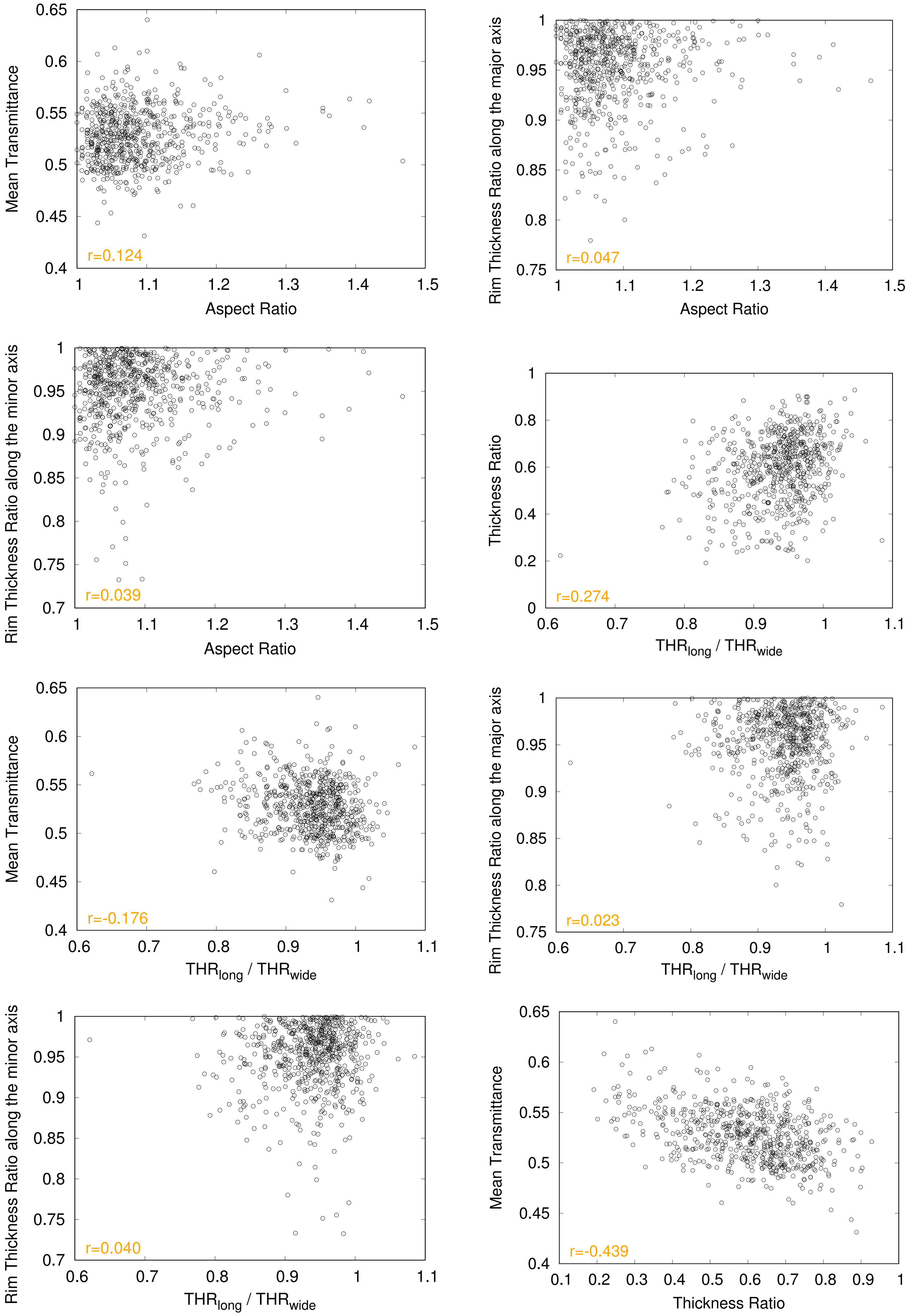

Mean Transmittance
Aspect Ratio
r=0.124
Rim Thickness Ratio along the major axis
Aspect Ratio
r=0.047
Rim Thickness Ratio along the minor axis
Aspect Ratio
r=0.039
Thickness Ratio
THR_long / THR_wide
r=0.274
Mean Transmittance
THR_long / THR_wide
r=-0.176
Rim Thickness Ratio along the major axis
THR_long / THR_wide
r=0.023
Rim Thickness Ratio along the minor axis
THR_long / THR_wide
r=0.040
Mean Transmittance
Thickness Ratio
r=-0.439

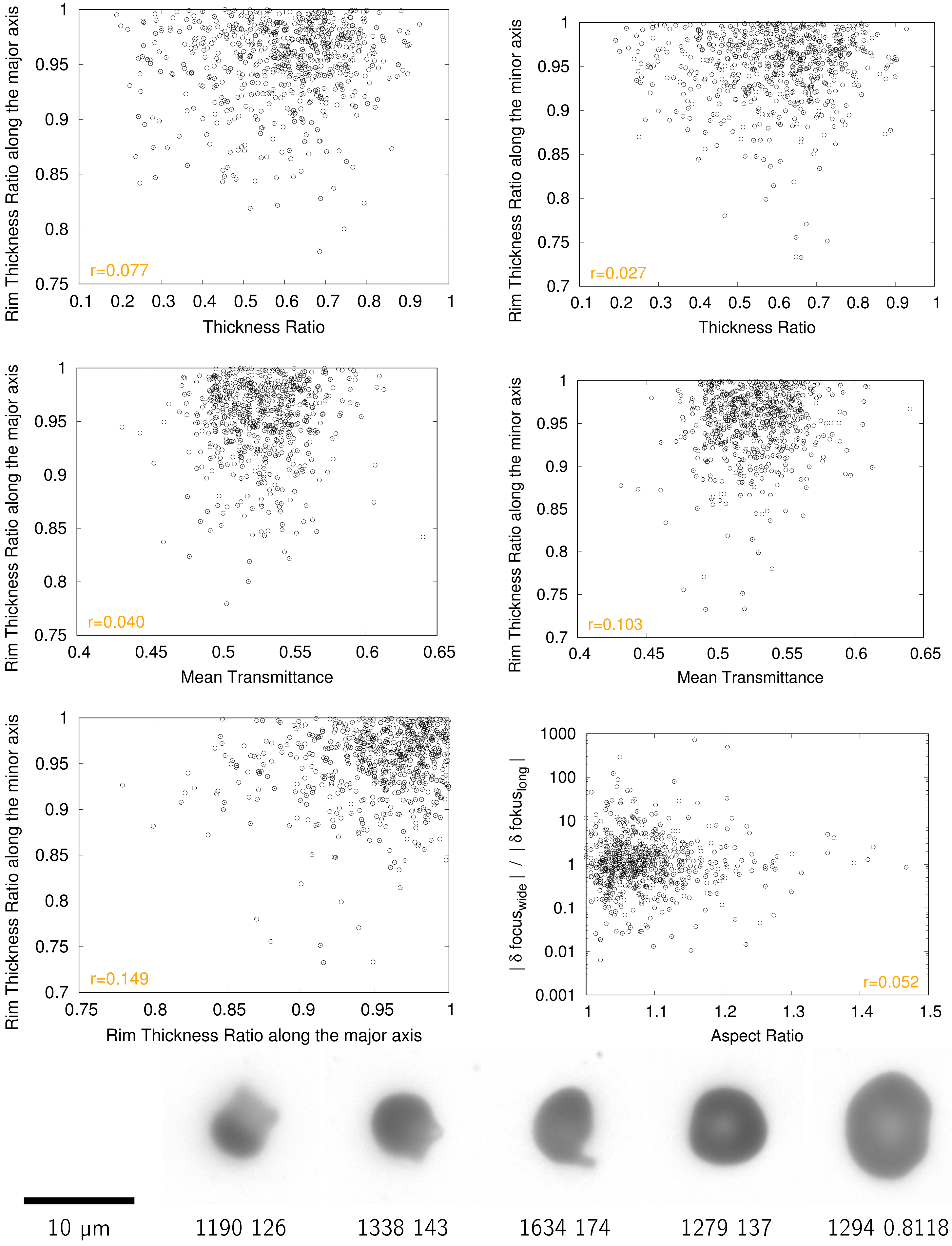

Rim Thickness Ratio along the major axis
Thickness Ratio
r=0.077
Rim Thickness Ratio along the minor axis
Thickness Ratio
r=0.027
Rim Thickness Ratio along the major axis
Mean Transmittance
r=0.040
Rim Thickness Ratio along the minor axis
Mean Transmittance
r=0.103
Rim Thickness Ratio along the minor axis
Rim Thickness Ratio along the major axis
r=0.149
| δ focus$_{wide}$ | / | δ fokus$_{long}$ |
Aspect Ratio
r=0.052
10 µm
1190 126
1338 143
1634 174
1279 137
1294 0.8118

Donor 7, plasma

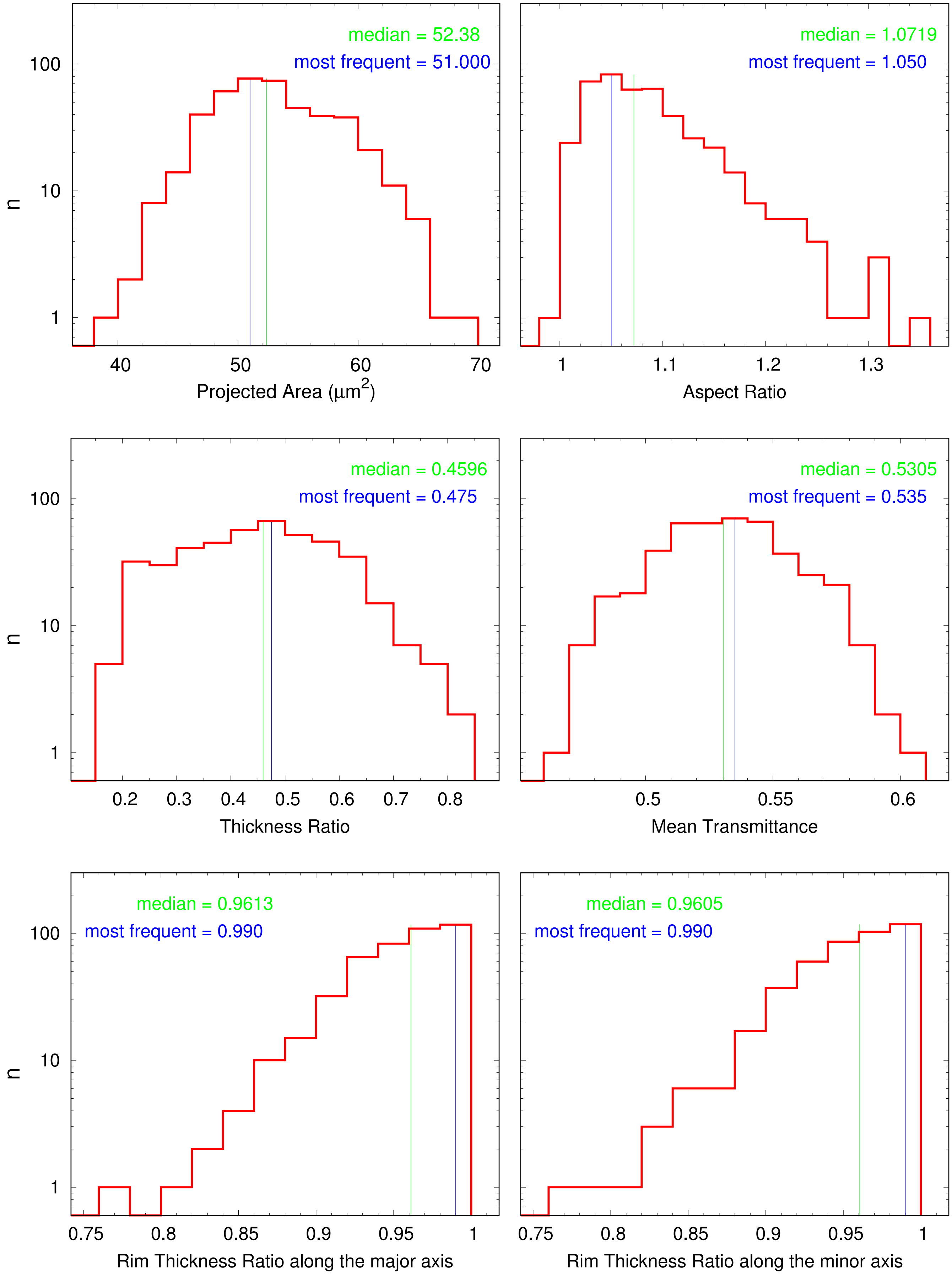

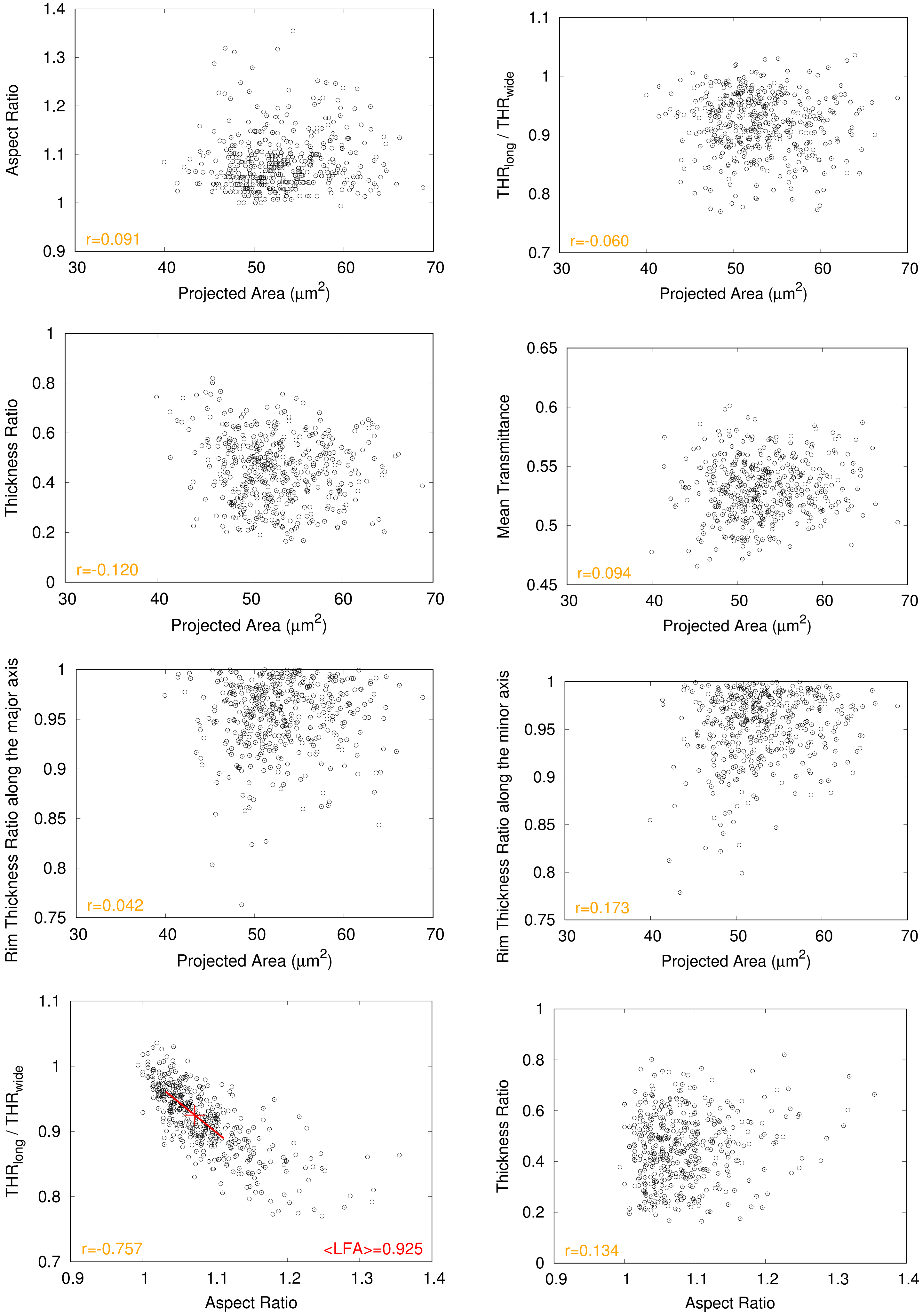

Aspect Ratio
Projected Area (μm²)
r=0.091
THR_long / THR_wide
Projected Area (μm²)
r=-0.060
Thickness Ratio
Projected Area (μm²)
r=-0.120
Mean Transmittance
Projected Area (μm²)
r=0.094
Rim Thickness Ratio along the major axis
Projected Area (μm²)
r=0.042
Rim Thickness Ratio along the minor axis
Projected Area (μm²)
r=0.173
THR_long / THR_wide
Aspect Ratio
r=-0.757
<LFA>=0.925
Thickness Ratio
Aspect Ratio
r=0.134

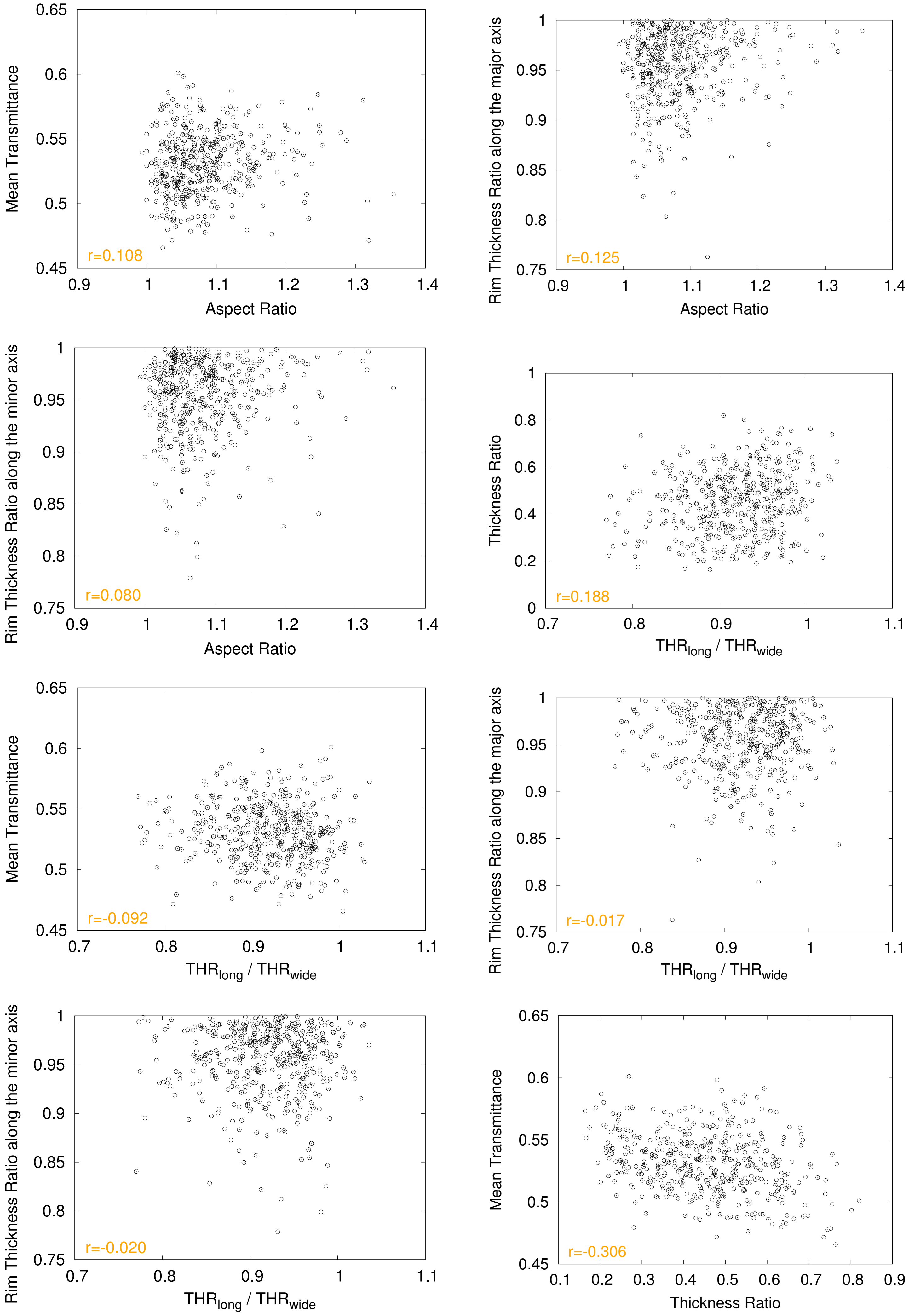
Mean Transmittance
Aspect Ratio
r=0.108
Rim Thickness Ratio along the major axis
Aspect Ratio
r=0.125
Rim Thickness Ratio along the minor axis
Aspect Ratio
r=0.080
Thickness Ratio
THR_{long} / THR_{wide}
r=0.188
Mean Transmittance
THR_{long} / THR_{wide}
r=-0.092
Rim Thickness Ratio along the major axis
THR_{long} / THR_{wide}
r=-0.017
Rim Thickness Ratio along the minor axis
THR_{long} / THR_{wide}
r=-0.020
Mean Transmittance
Thickness Ratio
r=-0.306

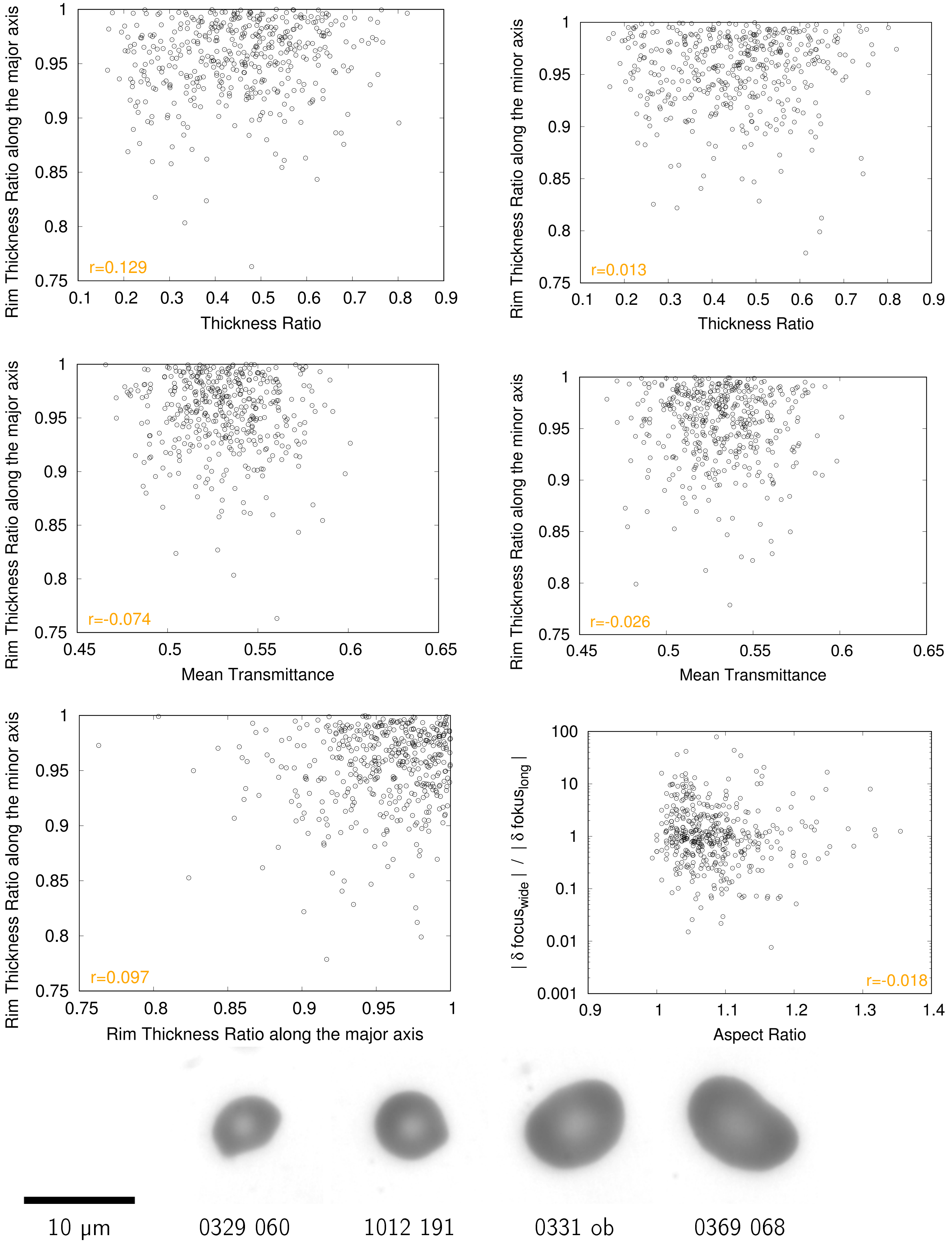

Rim Thickness Ratio along the major axis
Thickness Ratio
r=0.129
Rim Thickness Ratio along the minor axis
Thickness Ratio
r=0.013
Rim Thickness Ratio along the major axis
Mean Transmittance
r=-0.074
Rim Thickness Ratio along the minor axis
Mean Transmittance
r=-0.026
Rim Thickness Ratio along the minor axis
Rim Thickness Ratio along the major axis
r=0.097
| δ focus$_{wide}$ | / | δ fokus$_{long}$ |
Aspect Ratio
r=-0.018
10 μm
0329 060
1012 191
0331 ob
0369 068

Donor 7, serum washed

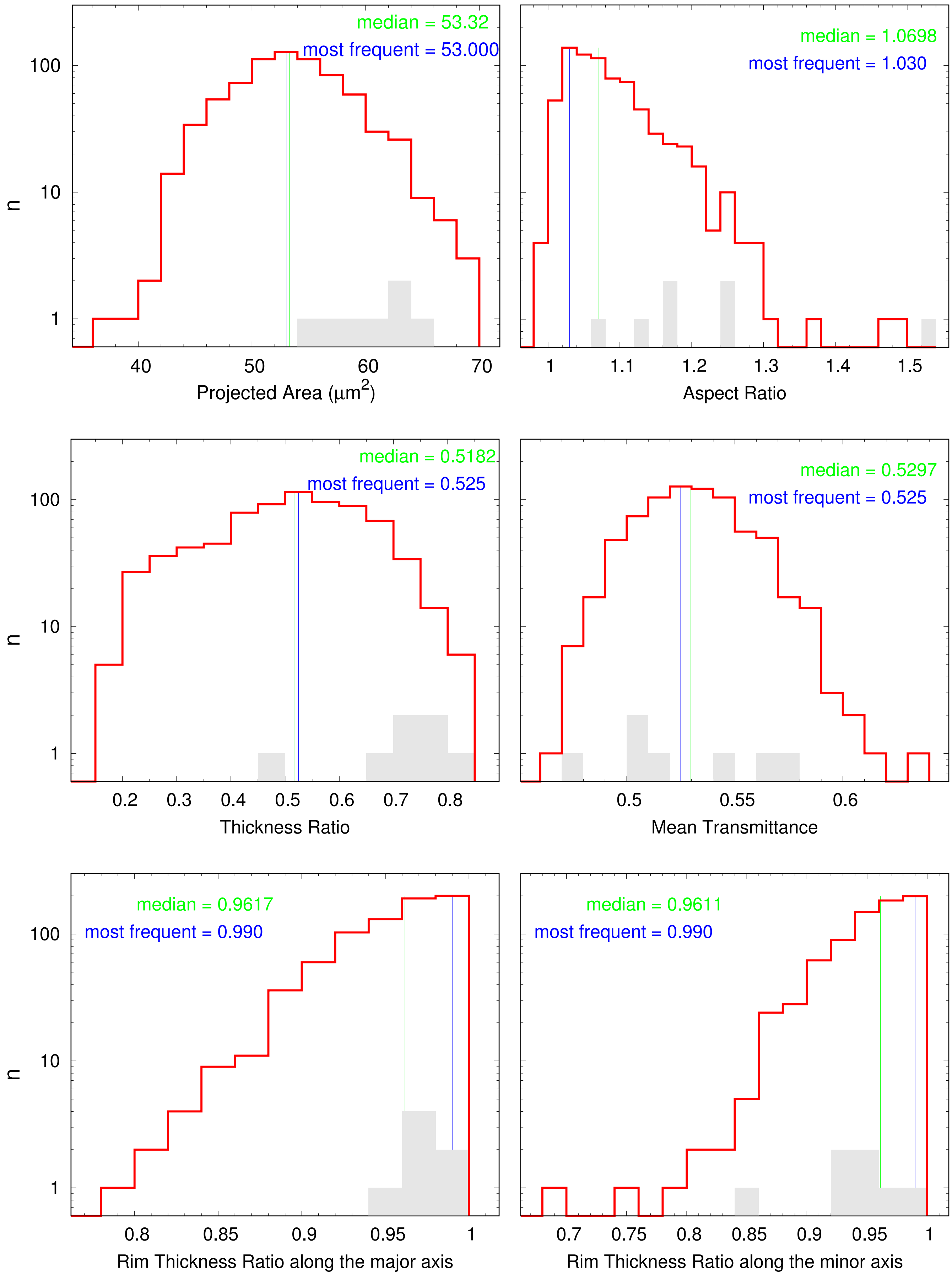

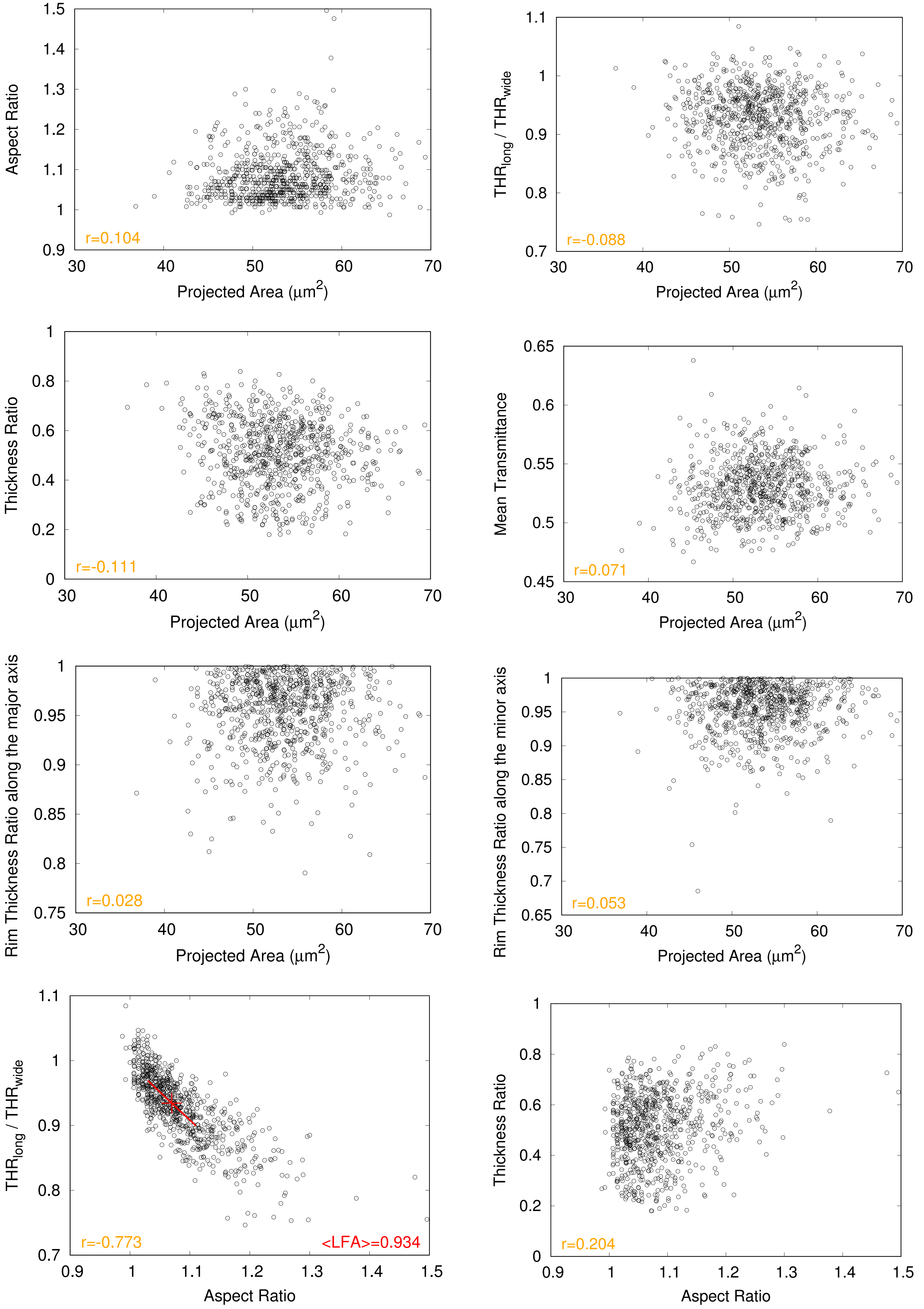
Aspect Ratio
Projected Area (μm^2)
r=0.104
THR_{long} / THR_{wide}
Projected Area (μm^2)
r=-0.088
Thickness Ratio
Projected Area (μm^2)
r=-0.111
Mean Transmittance
Projected Area (μm^2)
r=0.071
Rim Thickness Ratio along the major axis
Projected Area (μm^2)
r=0.028
Rim Thickness Ratio along the minor axis
Projected Area (μm^2)
r=0.053
THR_{long} / THR_{wide}
Aspect Ratio
r=-0.773
<LFA>=0.934
Thickness Ratio
Aspect Ratio
r=0.204

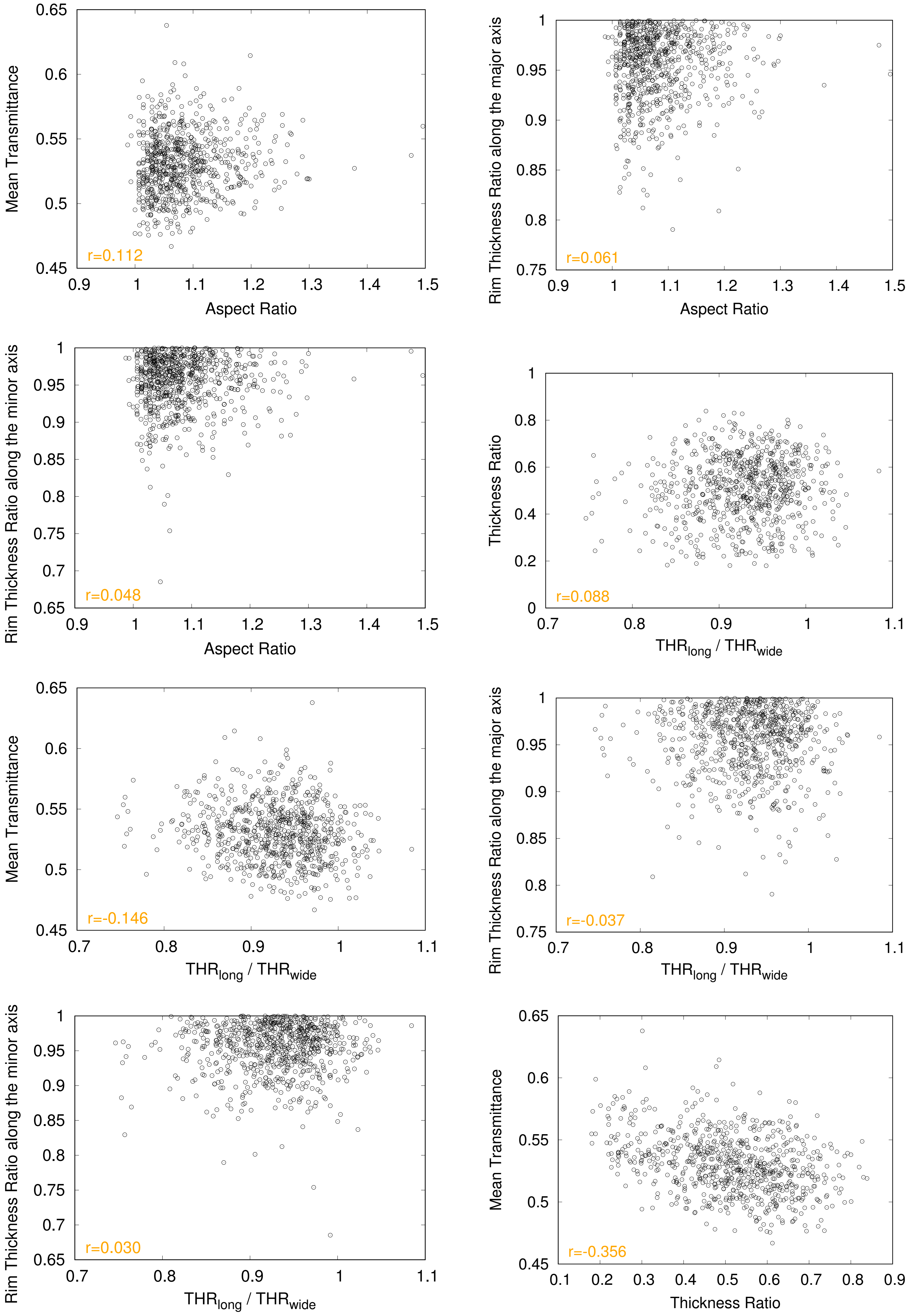
Mean Transmittance
Aspect Ratio
r=0.112
Rim Thickness Ratio along the major axis
Aspect Ratio
r=0.061
Rim Thickness Ratio along the minor axis
Aspect Ratio
r=0.048
Thickness Ratio
THR_{long} / THR_{wide}
r=0.088
Mean Transmittance
THR_{long} / THR_{wide}
r=-0.146
Rim Thickness Ratio along the major axis
THR_{long} / THR_{wide}
r=-0.037
Rim Thickness Ratio along the minor axis
THR_{long} / THR_{wide}
r=0.030
Mean Transmittance
Thickness Ratio
r=-0.356

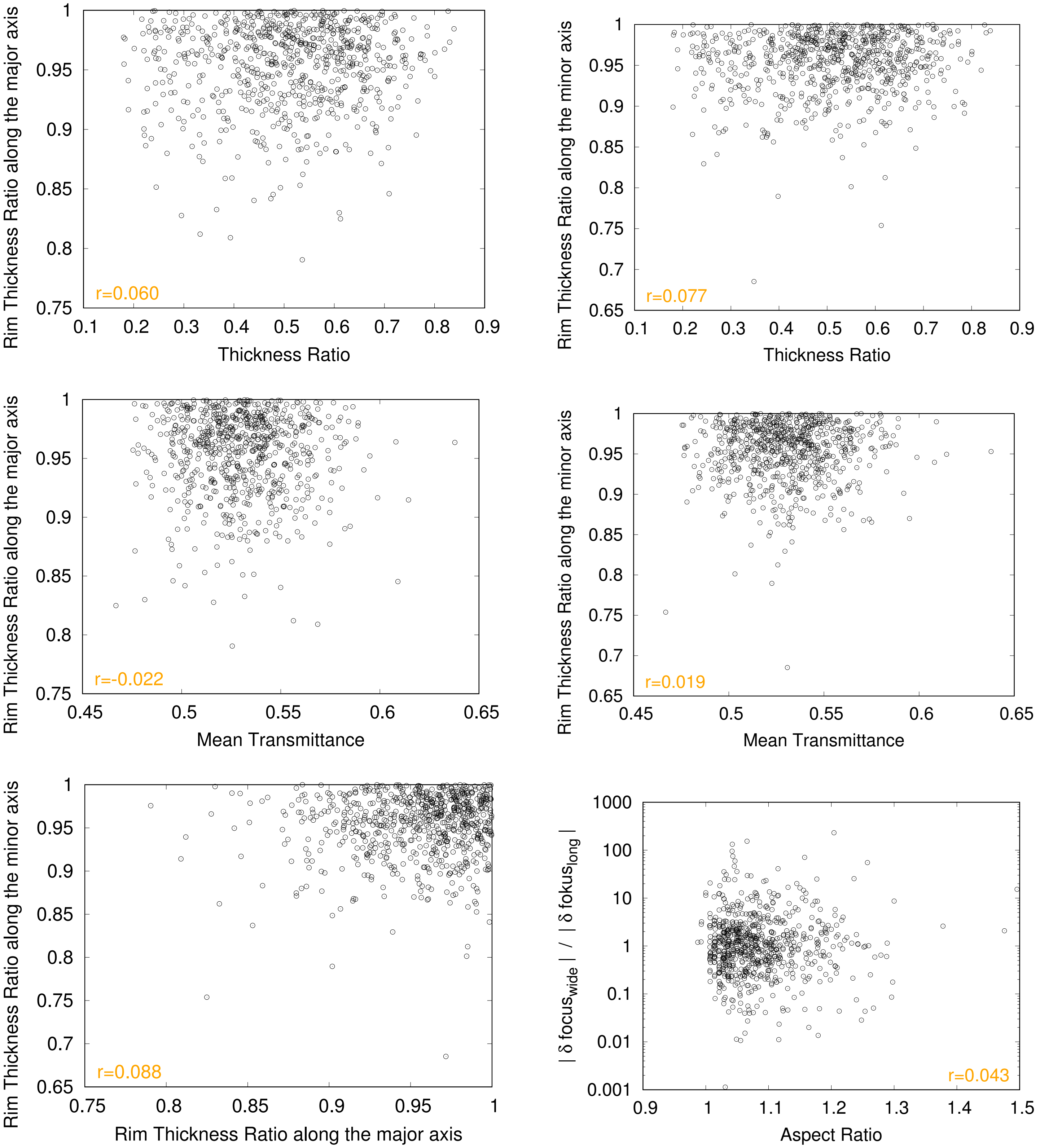
Rim Thickness Ratio along the major axis
Thickness Ratio
r=0.060
Rim Thickness Ratio along the minor axis
Thickness Ratio
r=0.077
Rim Thickness Ratio along the major axis
Mean Transmittance
r=-0.022
Rim Thickness Ratio along the minor axis
Mean Transmittance
r=0.019
Rim Thickness Ratio along the minor axis
Rim Thickness Ratio along the major axis
r=0.088
| δ focus$_{wide}$ | / | δ fokus$_{long}$ |
Aspect Ratio
r=0.043

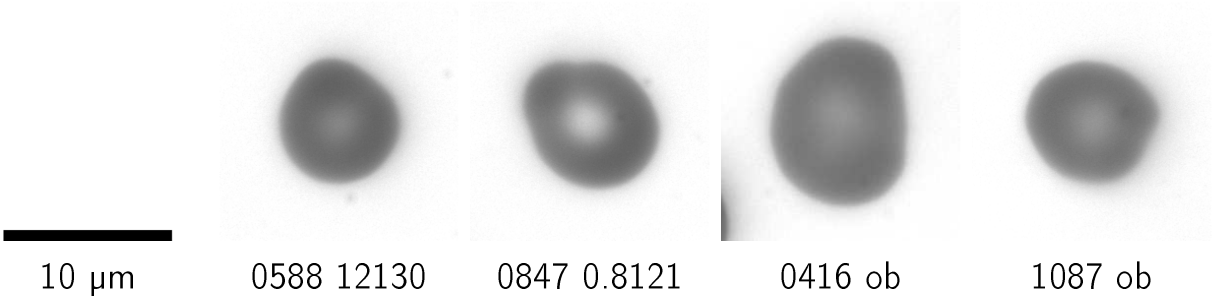
10 µm
0588 12130
0847 0.8121
0416 ob
1087 ob

Donor 8, plasma

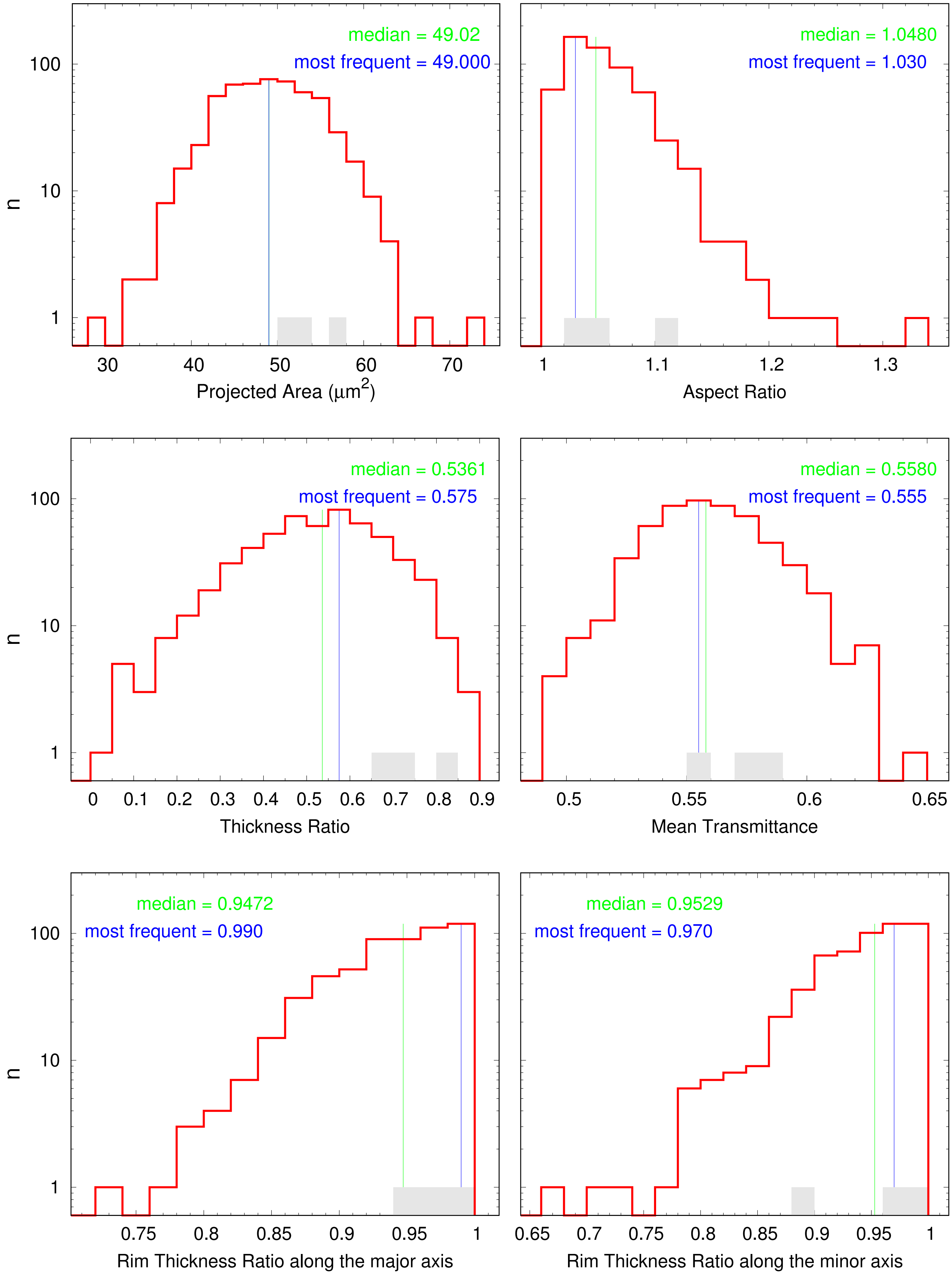

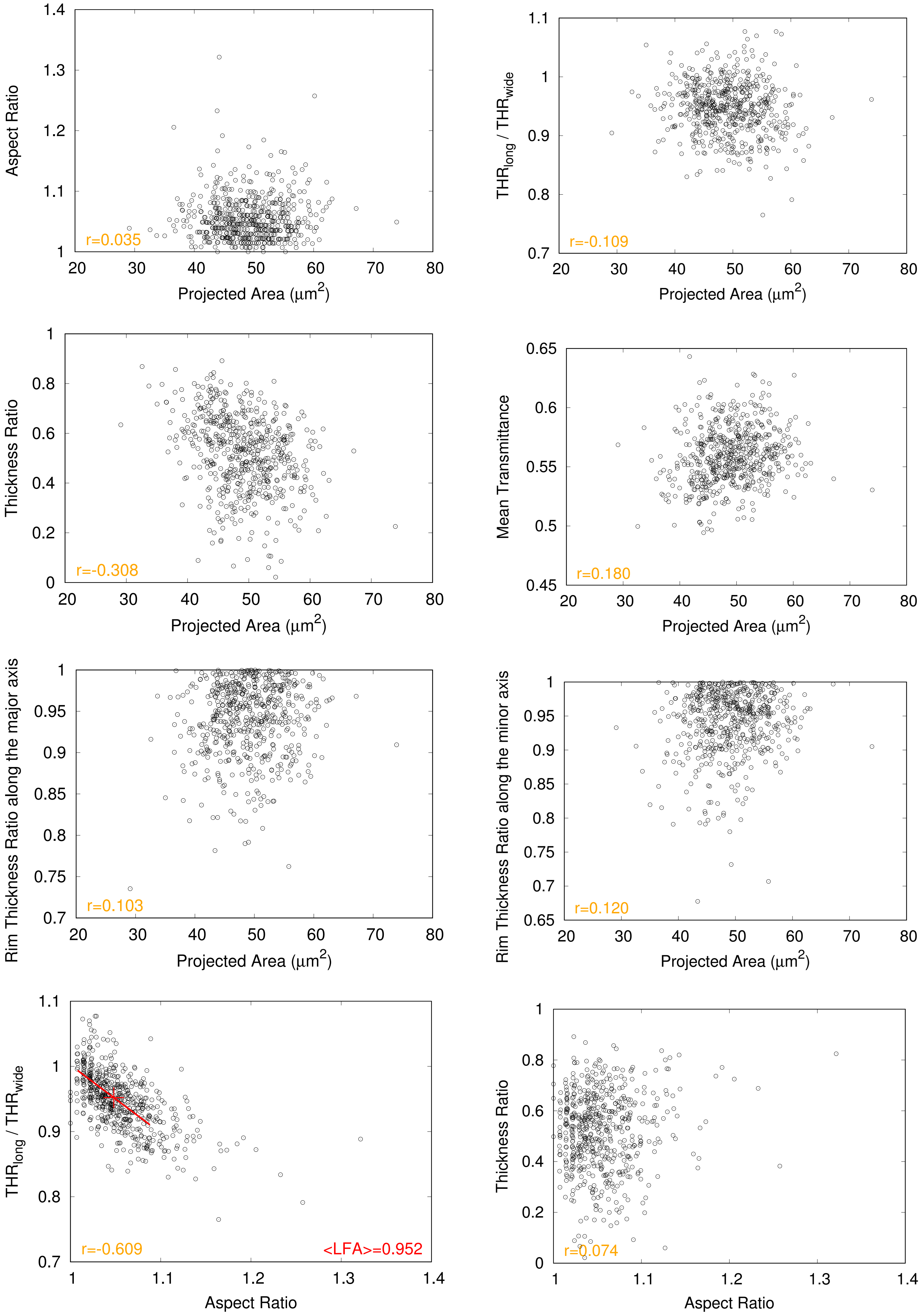
Aspect Ratio
Projected Area (μm2)
r=0.035
THRlong / THRwide
Projected Area (μm2)
r=-0.109
Thickness Ratio
Projected Area (μm2)
r=-0.308
Mean Transmittance
Projected Area (μm2)
r=0.180
Rim Thickness Ratio along the major axis
Projected Area (μm2)
r=0.103
Rim Thickness Ratio along the minor axis
Projected Area (μm2)
r=0.120
THRlong / THRwide
Aspect Ratio
r=-0.609
<LFA>=0.952
Thickness Ratio
Aspect Ratio
r=0.074

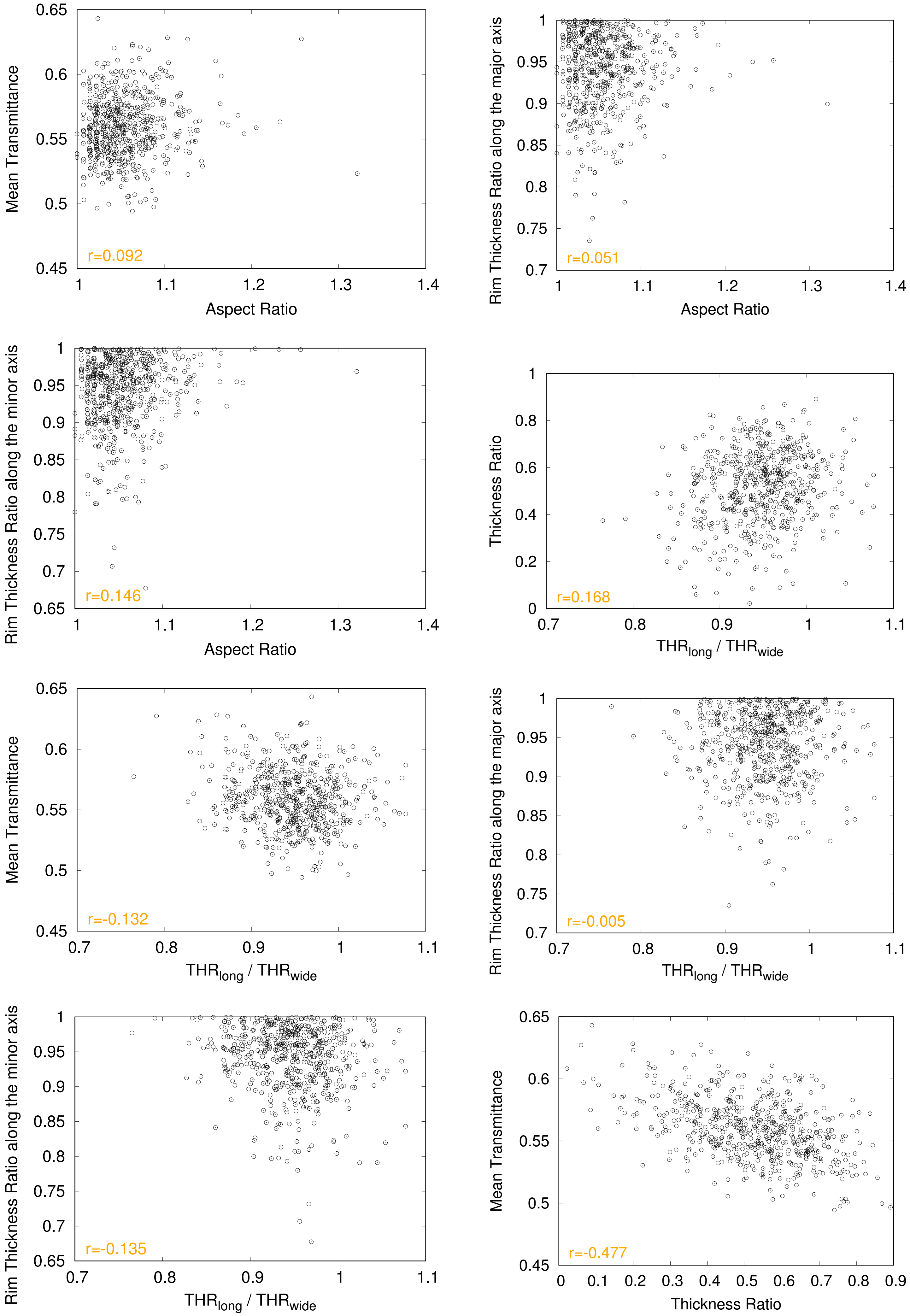

Mean Transmittance
Aspect Ratio
r=0.092
Rim Thickness Ratio along the major axis
Aspect Ratio
r=0.051
Rim Thickness Ratio along the minor axis
Aspect Ratio
r=0.146
Thickness Ratio
THR_{long} / THR_{wide}
r=0.168
Mean Transmittance
THR_{long} / THR_{wide}
r=-0.132
Rim Thickness Ratio along the major axis
THR_{long} / THR_{wide}
r=-0.005
Rim Thickness Ratio along the minor axis
THR_{long} / THR_{wide}
r=-0.135
Mean Transmittance
Thickness Ratio
r=-0.477

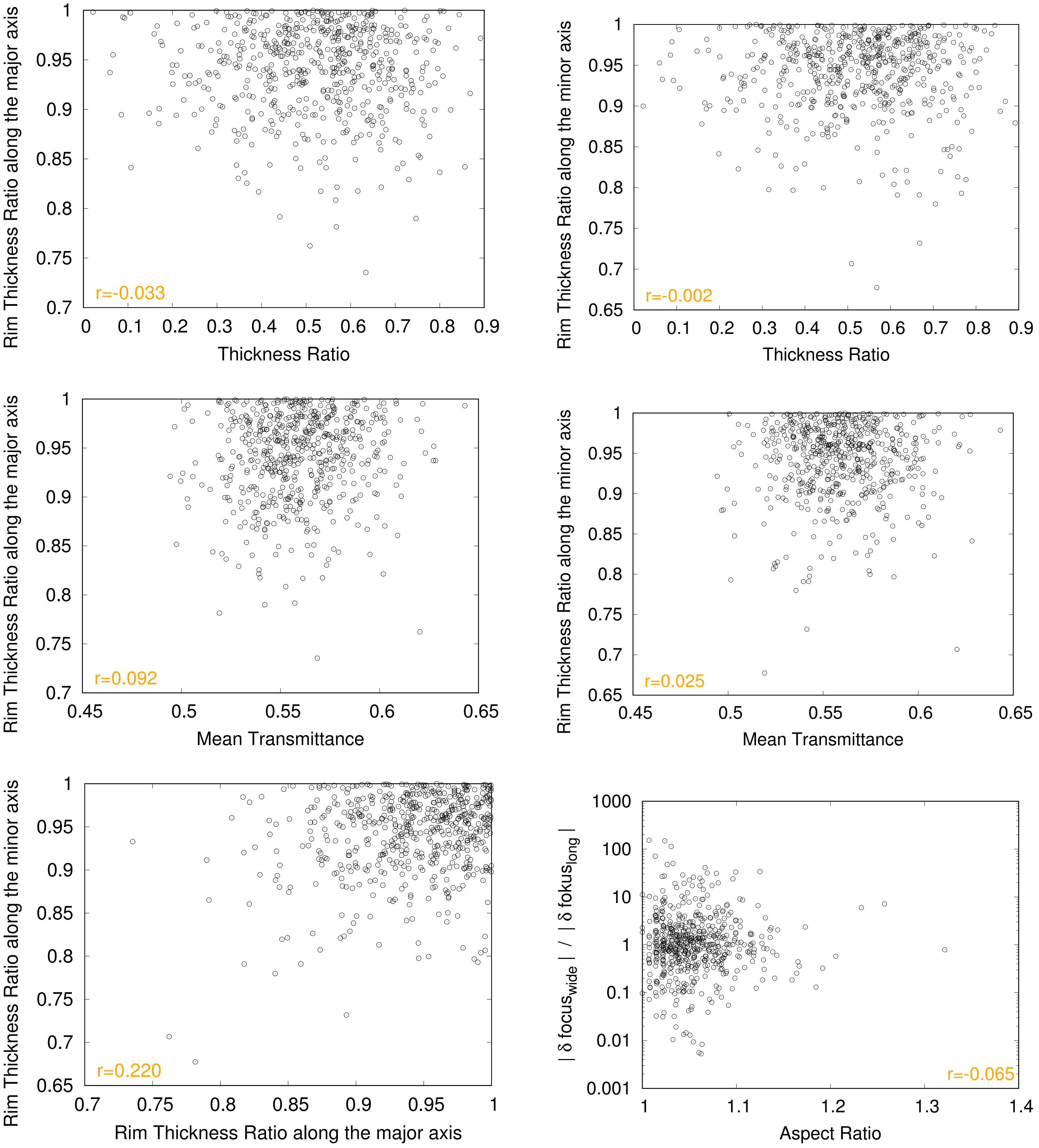

Rim Thickness Ratio along the major axis
Thickness Ratio
r=-0.033
Rim Thickness Ratio along the minor axis
Thickness Ratio
r=-0.002
Rim Thickness Ratio along the major axis
Mean Transmittance
r=0.092
Rim Thickness Ratio along the minor axis
Mean Transmittance
r=0.025
Rim Thickness Ratio along the minor axis
Rim Thickness Ratio along the major axis
r=0.220
| δ focus_wide | / | δ fokus_long |
Aspect Ratio
r=-0.065

Donor 8, serum washed

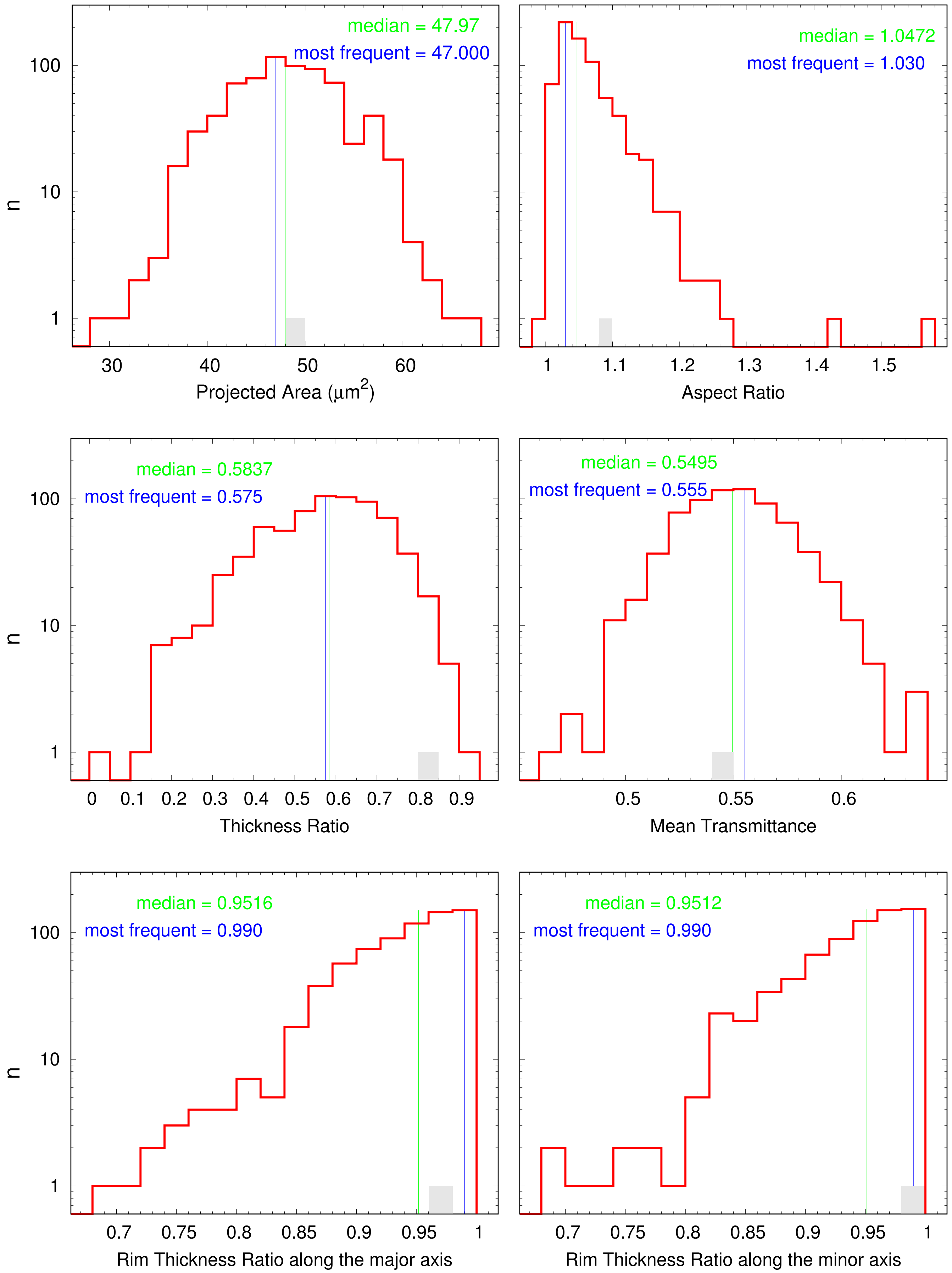

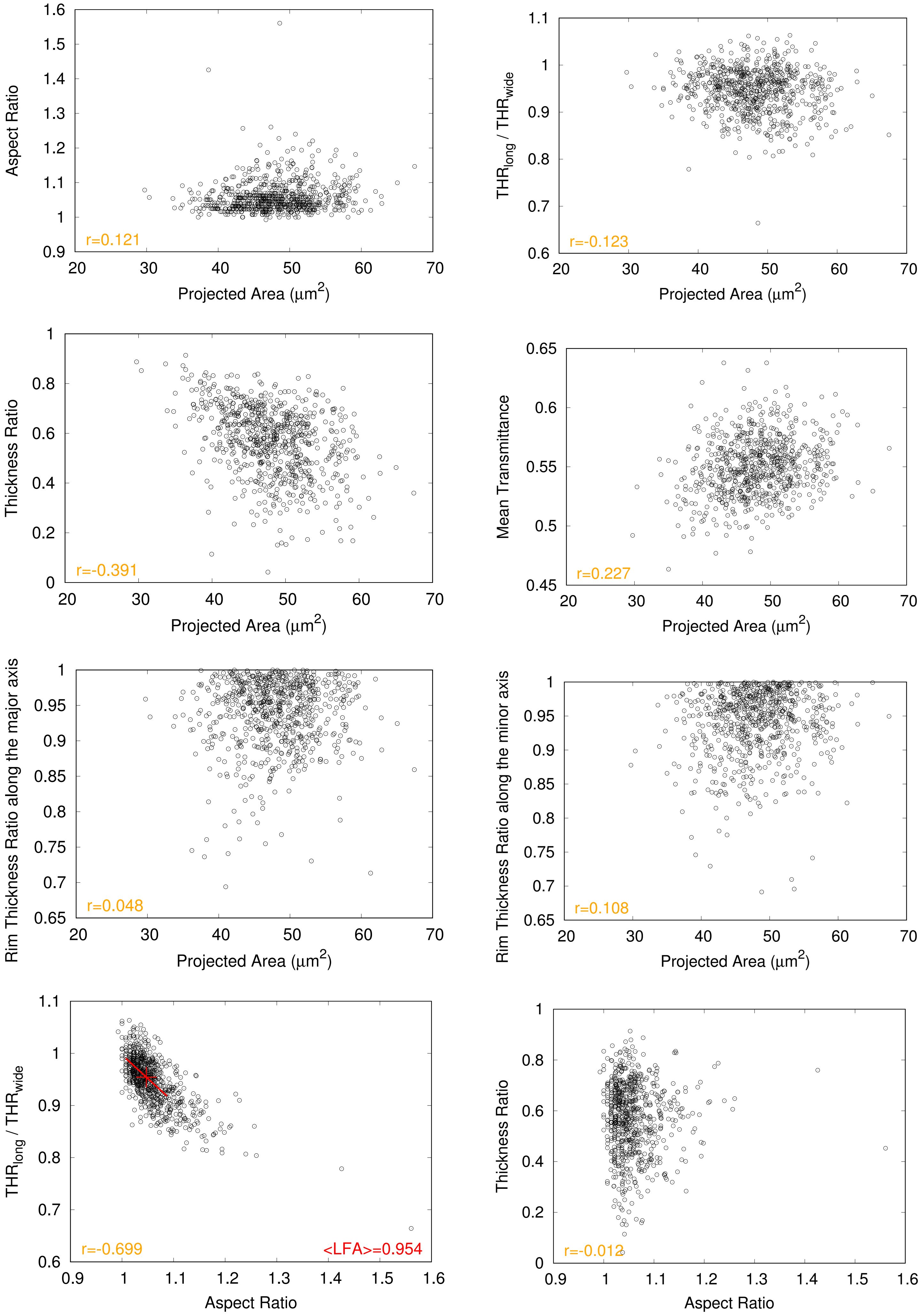

Aspect Ratio
Projected Area (μm2)
r=0.121
THRlong / THRwide
Projected Area (μm2)
r=-0.123
Thickness Ratio
Projected Area (μm2)
r=-0.391
Mean Transmittance
Projected Area (μm2)
r=0.227
Rim Thickness Ratio along the major axis
Projected Area (μm2)
r=0.048
Rim Thickness Ratio along the minor axis
Projected Area (μm2)
r=0.108
THRlong / THRwide
Aspect Ratio
r=-0.699
<LFA>=0.954
Thickness Ratio
Aspect Ratio
r=-0.012

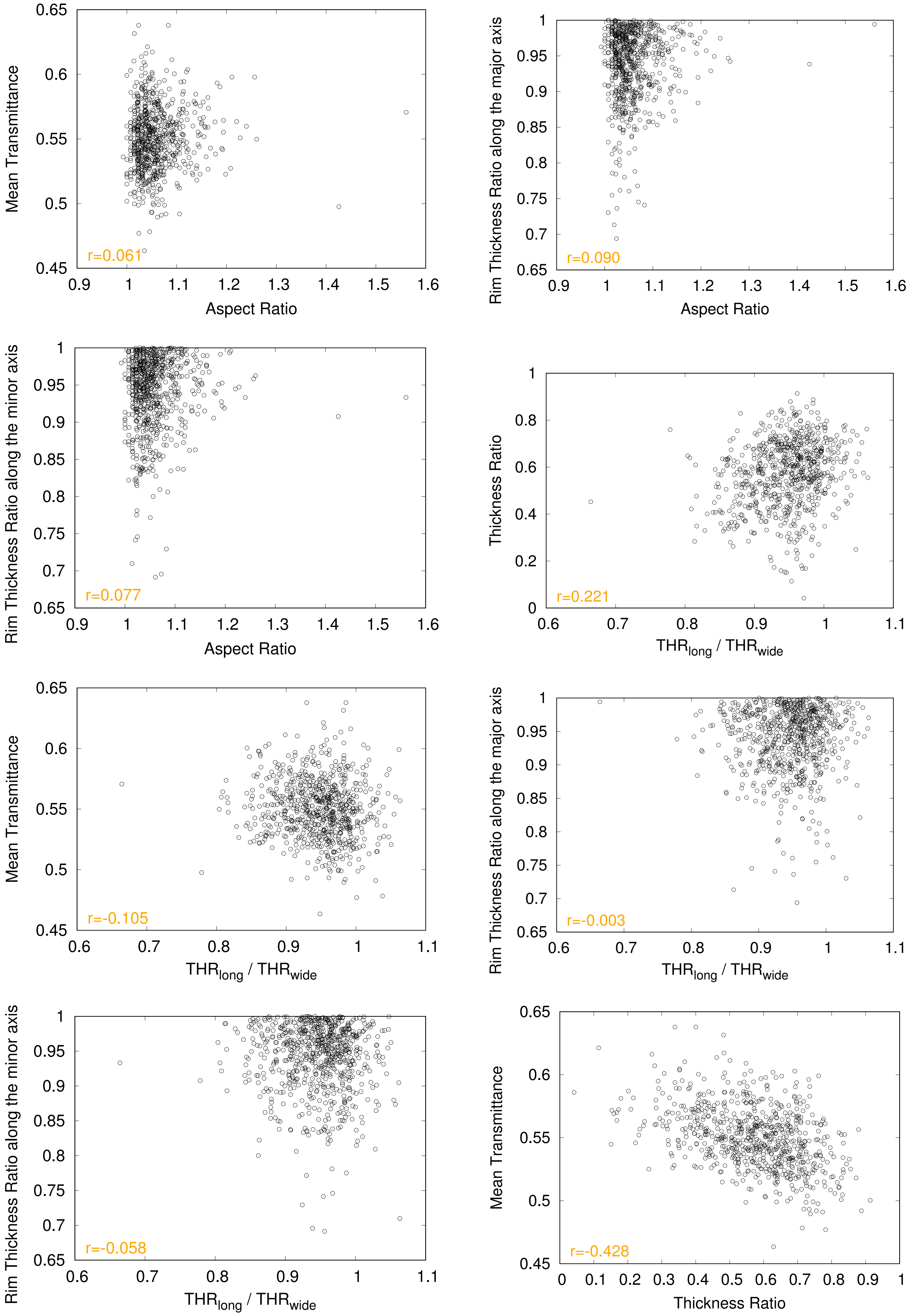
Mean Transmittance
Aspect Ratio
r=0.061
Rim Thickness Ratio along the major axis
Aspect Ratio
r=0.090
Rim Thickness Ratio along the minor axis
Aspect Ratio
r=0.077
Thickness Ratio
THR_{long} / THR_{wide}
r=0.221
Mean Transmittance
THR_{long} / THR_{wide}
r=-0.105
Rim Thickness Ratio along the major axis
THR_{long} / THR_{wide}
r=-0.003
Rim Thickness Ratio along the minor axis
THR_{long} / THR_{wide}
r=-0.058
Mean Transmittance
Thickness Ratio
r=-0.428

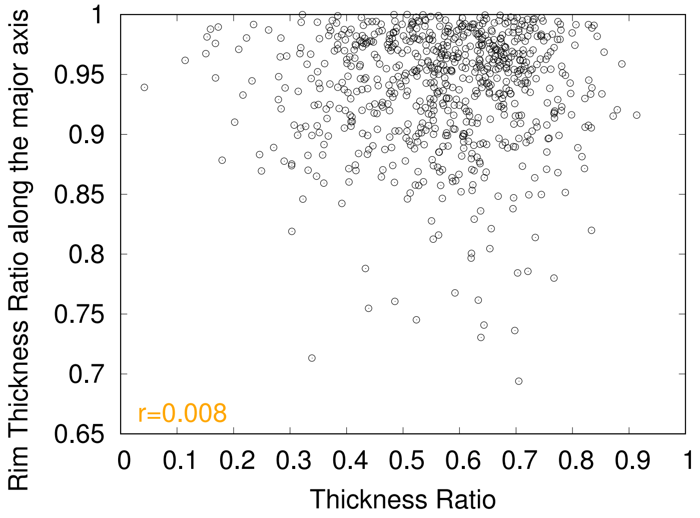
Rim Thickness Ratio along the major axis
Thickness Ratio
r=0.008

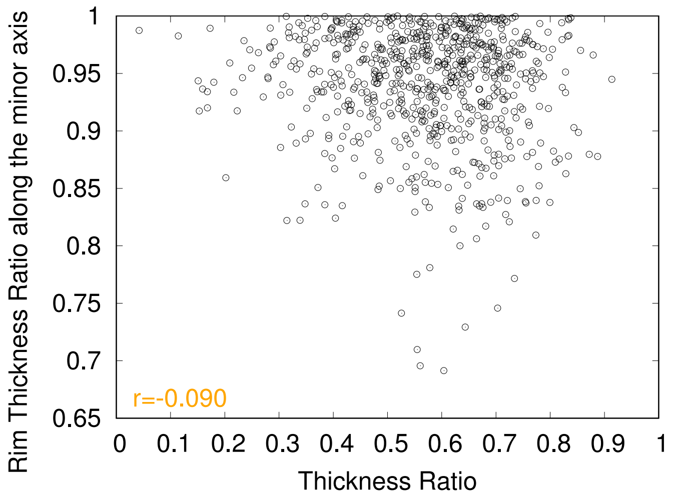
Rim Thickness Ratio along the minor axis
Thickness Ratio
r=-0.090

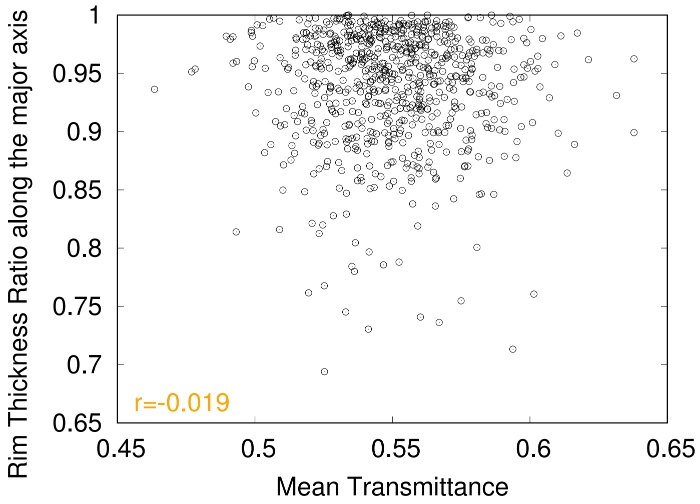
Rim Thickness Ratio along the major axis
Mean Transmittance
r=-0.019

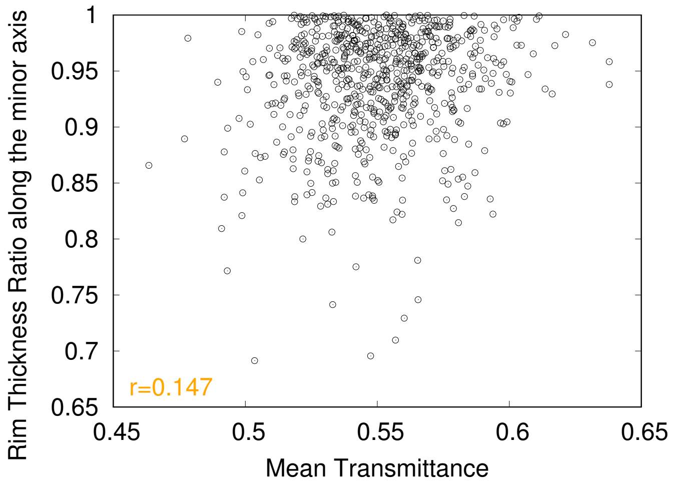
Rim Thickness Ratio along the minor axis
Mean Transmittance
r=0.147

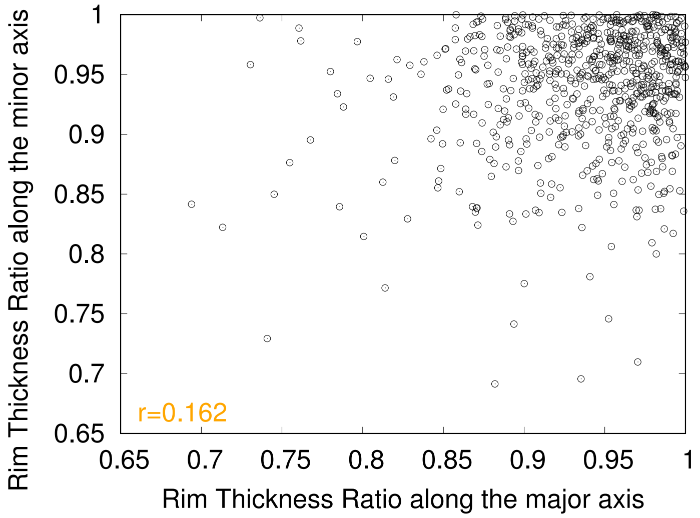
Rim Thickness Ratio along the minor axis
Rim Thickness Ratio along the major axis
r=0.162

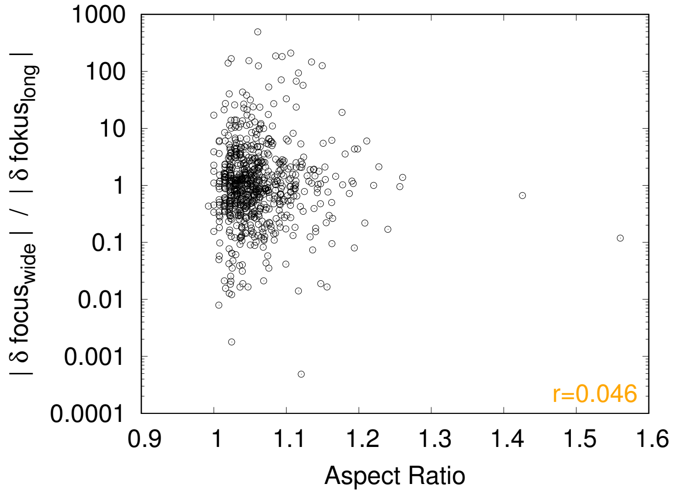
| δ focuswide | / | δ fokuslong |
Aspect Ratio
r=0.046

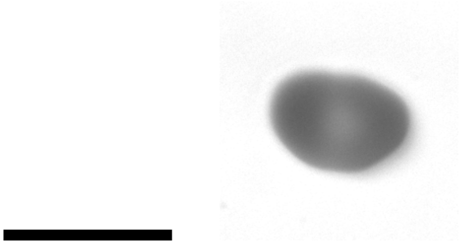

10 μm   0895 ob
