# Supplementary figures and images for: The Shape of Human Red Blood Cells Suspended in Autologous Plasma and Serum
Source: Cells. 2022 Jun 16;11(12):1941. doi: 10.3390/cells11121941 (PMC9222013; doi:10.3390/cells11121941)

donor

plasma

serum, unwashed

1

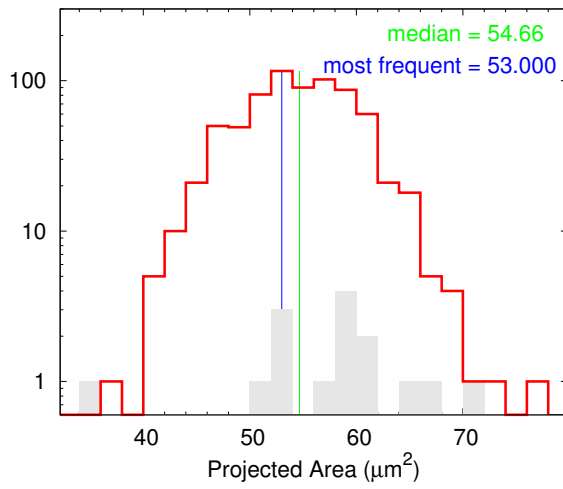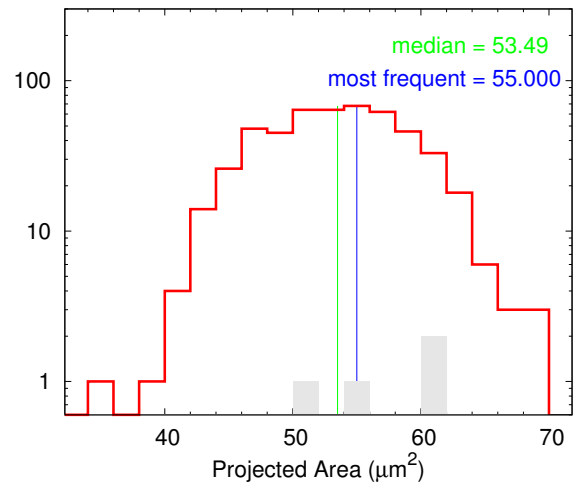

2

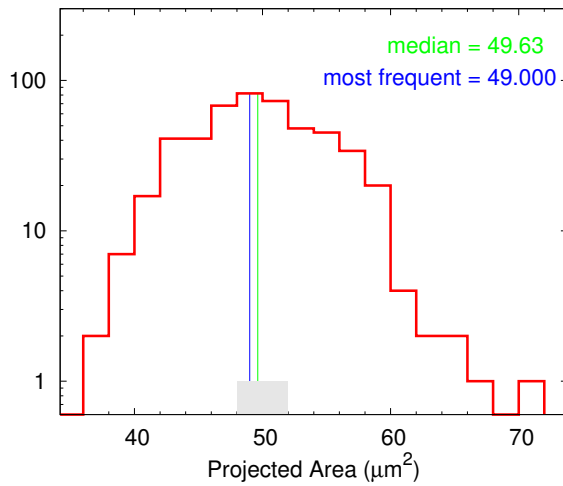

3

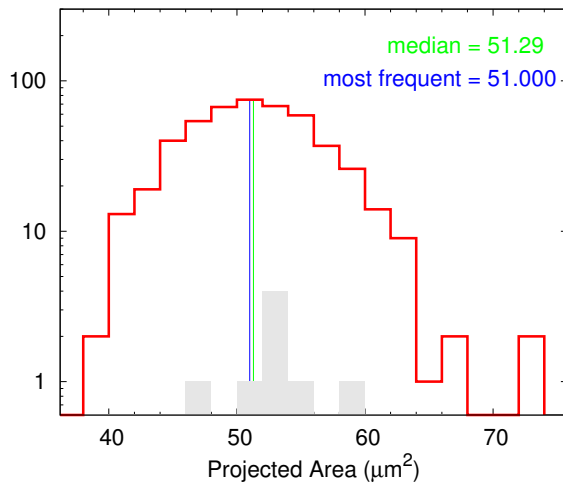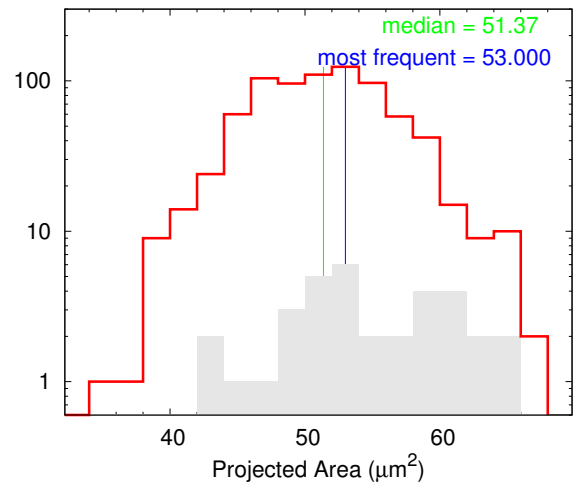

donor

plasma

serum, washed

3

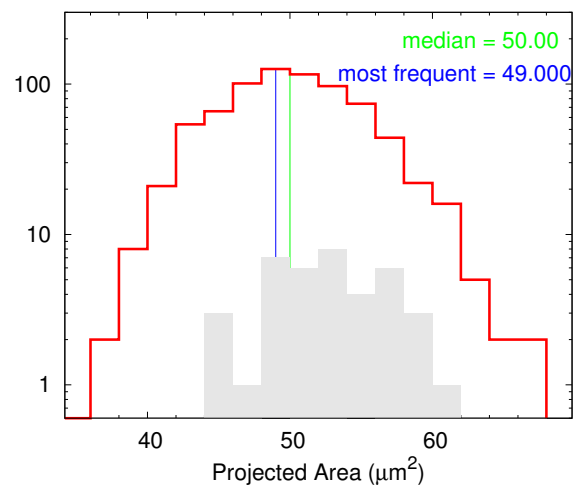

donor

plasma

serum, washed

4

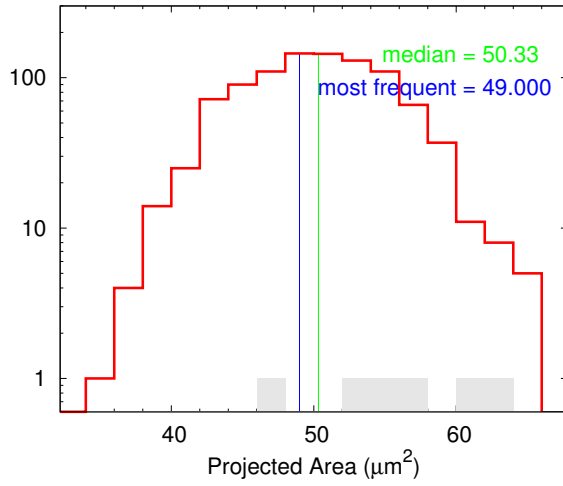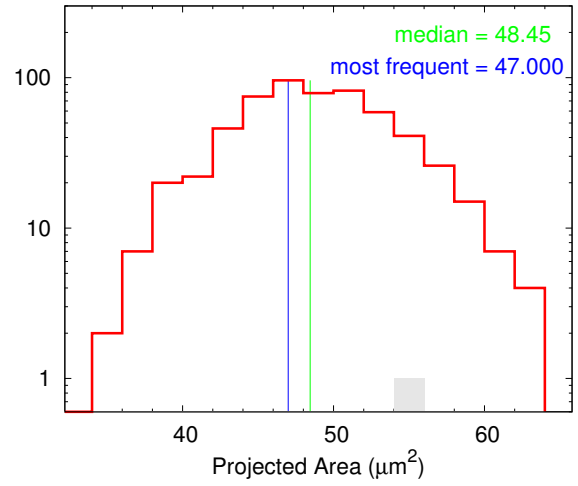

5

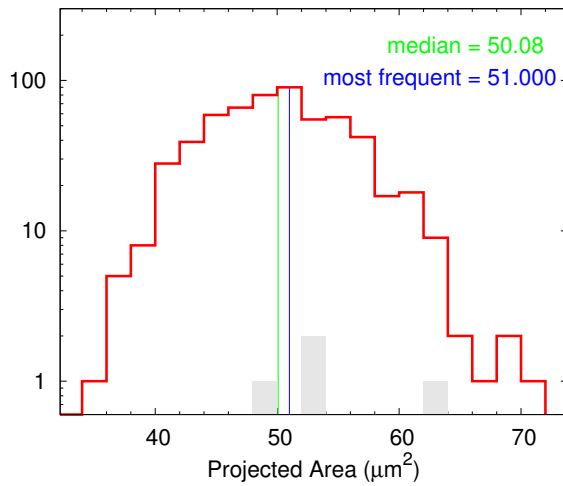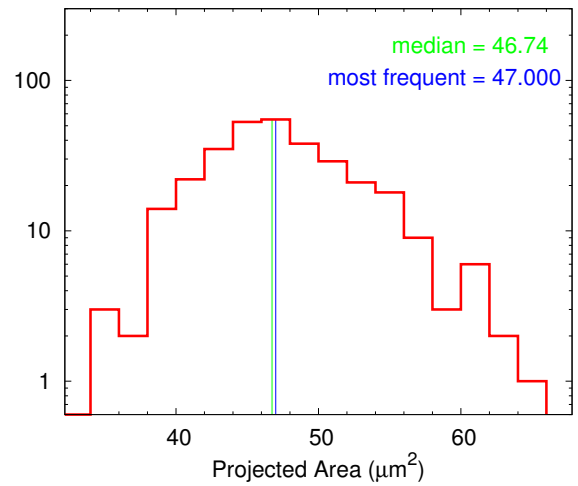

6

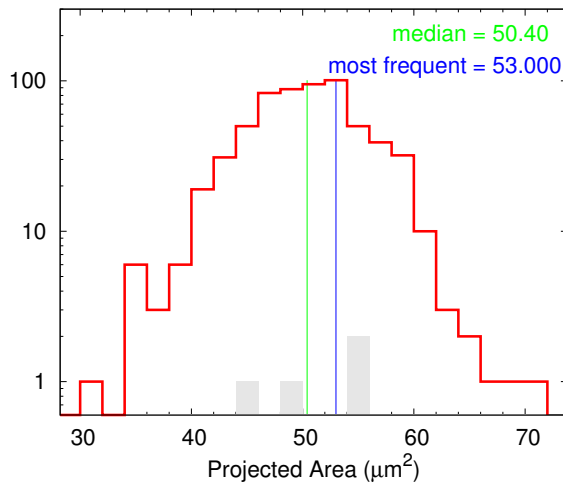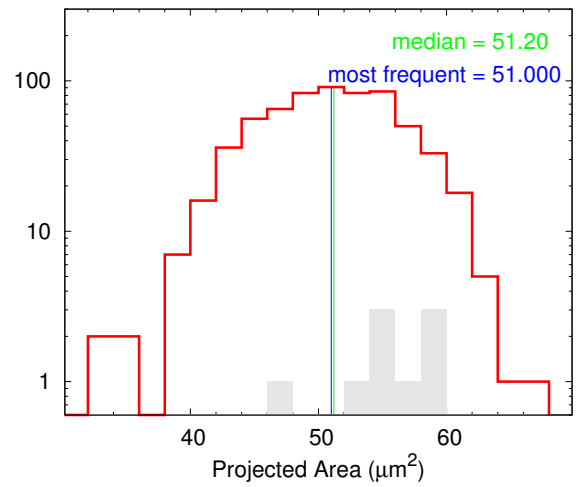

7

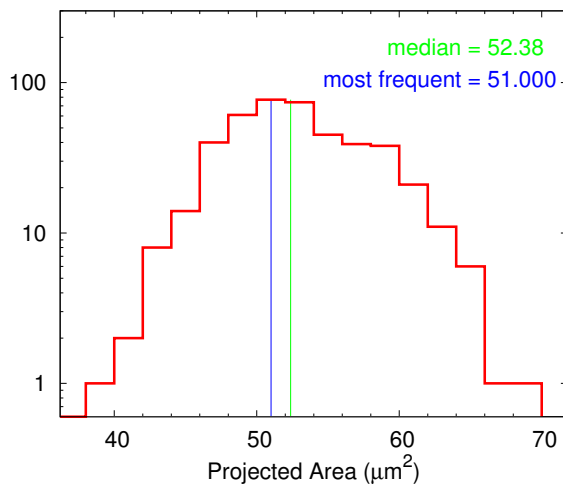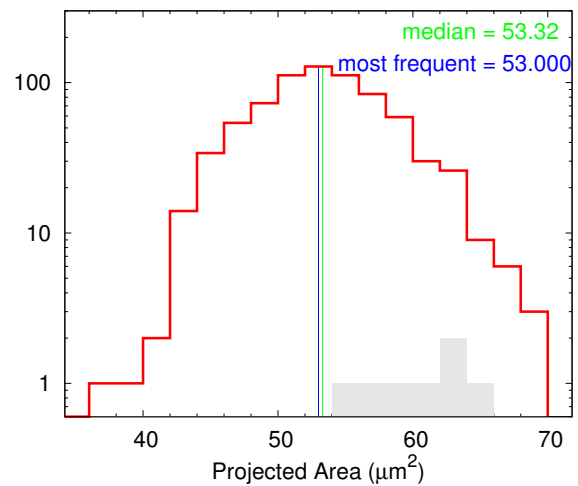

8

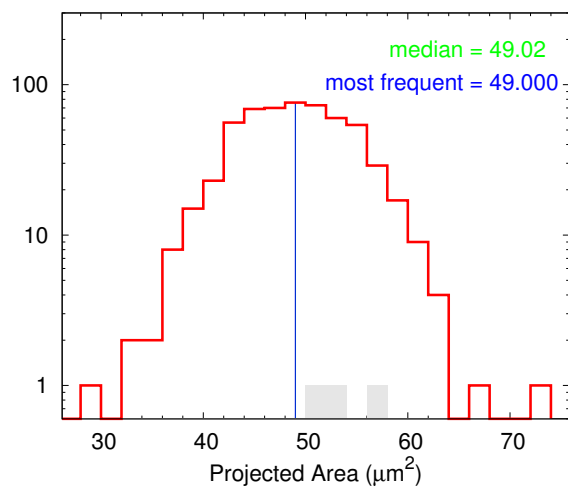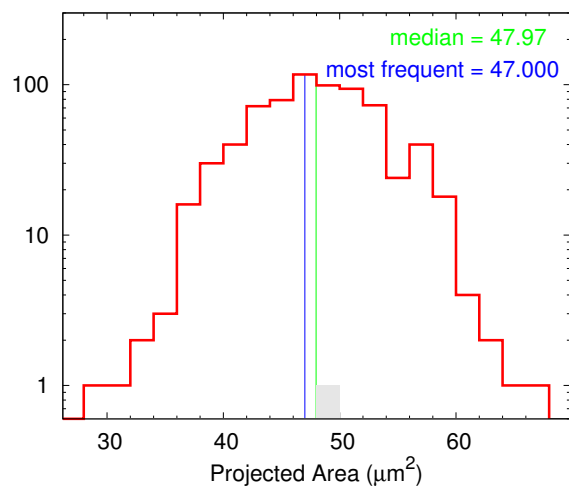

Supplement: Supplementary file 1 [file cells-11-01941-s001.zip › cells-1742284-supplementary/Documents S2-S6/cells-1742284 - Suppl. Document S3.pdf]

donor

plasma

serum, unwashed

1

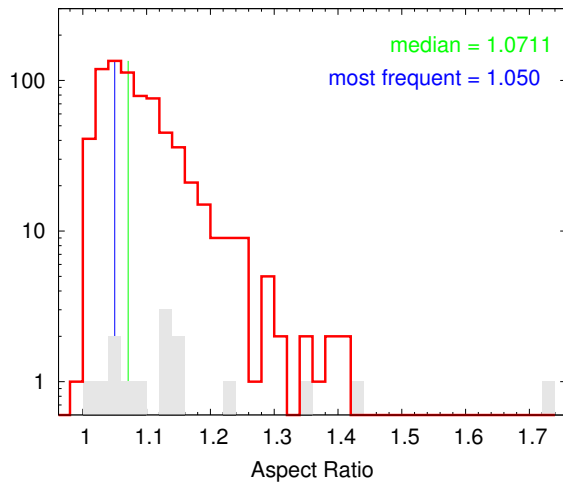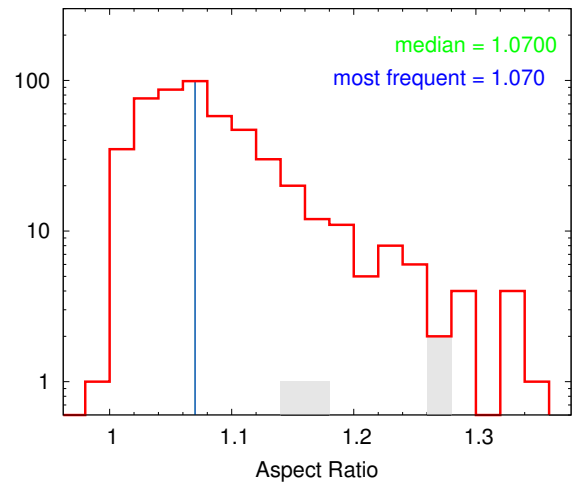

2

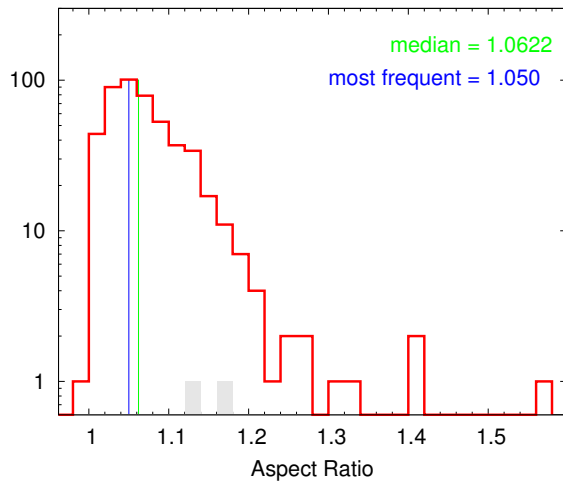

3

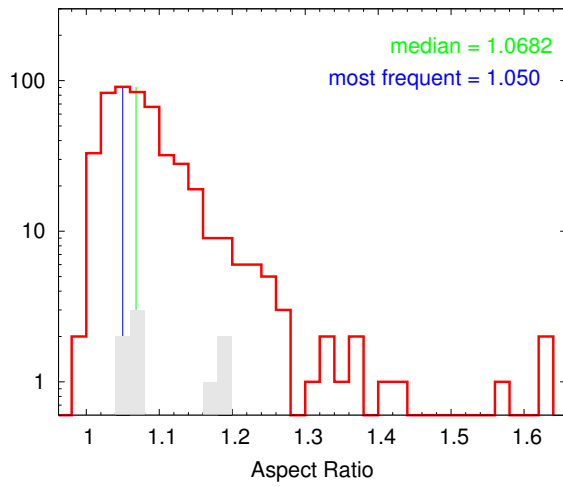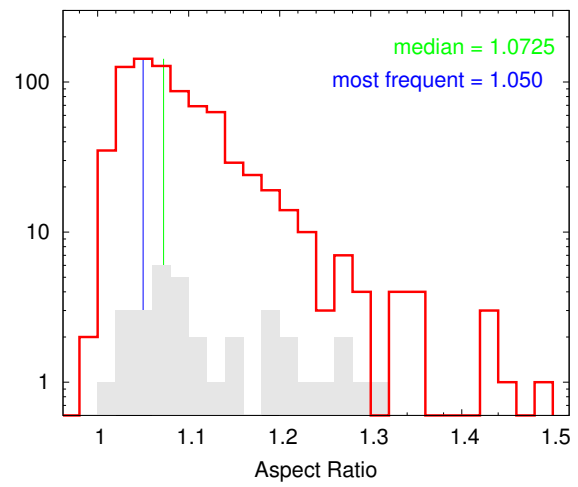

donor

plasma

serum, washed

3

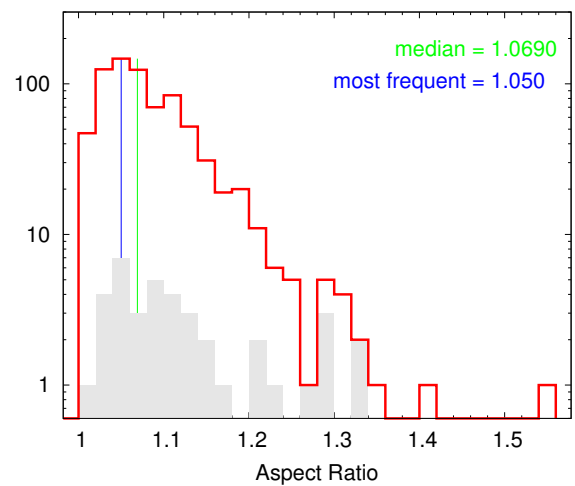

4

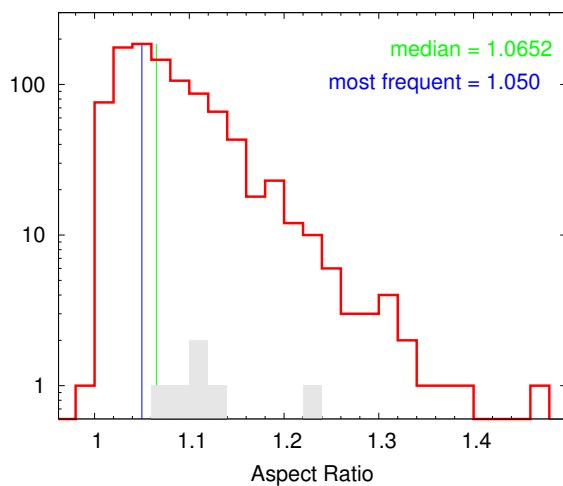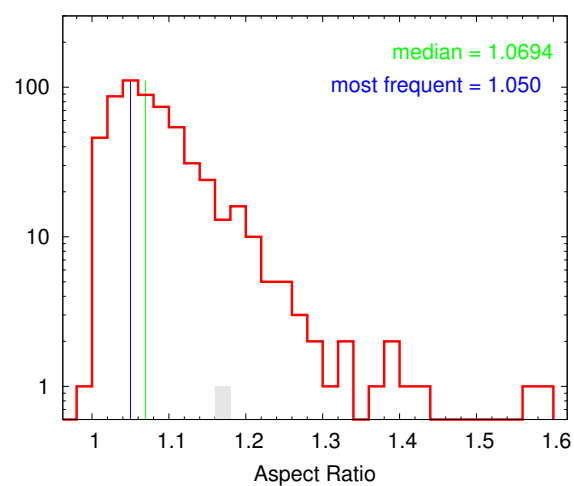

5

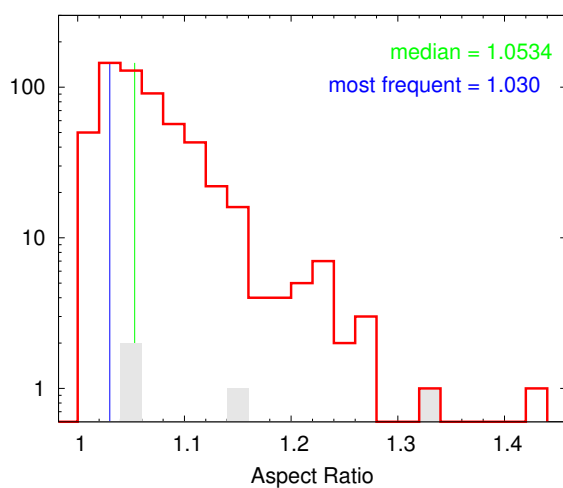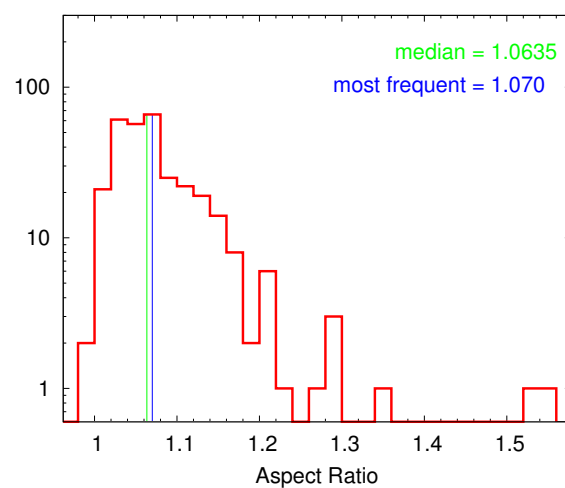

6

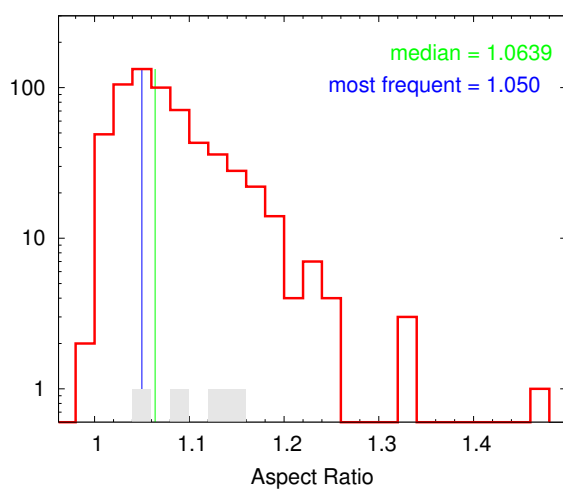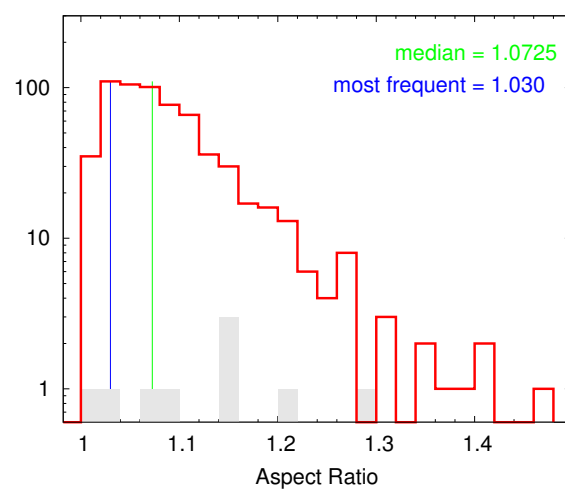

7

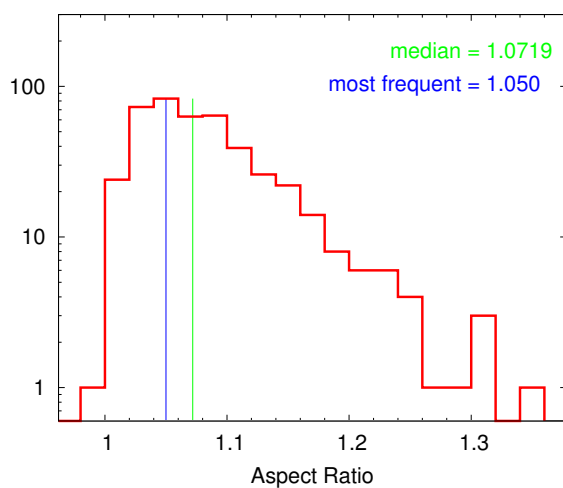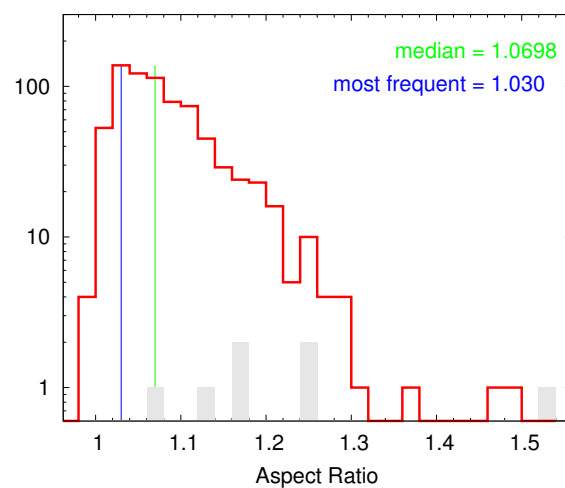

8

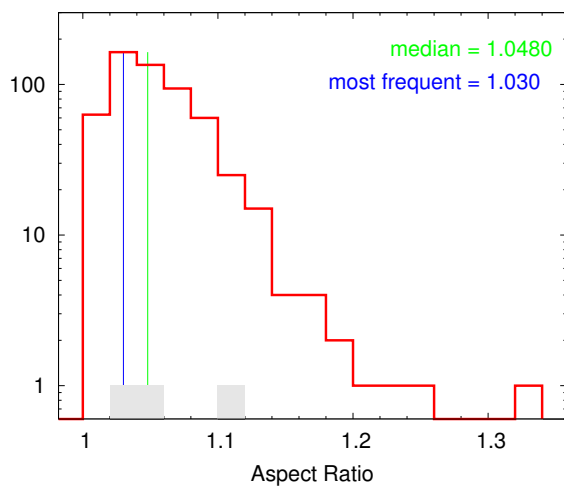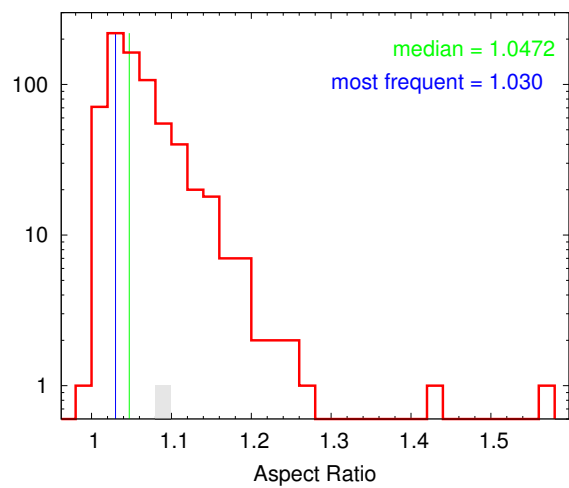

Supplement: Supplementary file 1 [file cells-11-01941-s001.zip › cells-1742284-supplementary/Documents S2-S6/cells-1742284 - Suppl. Document S4.pdf]

donor

plasma

serum, unwashed

1

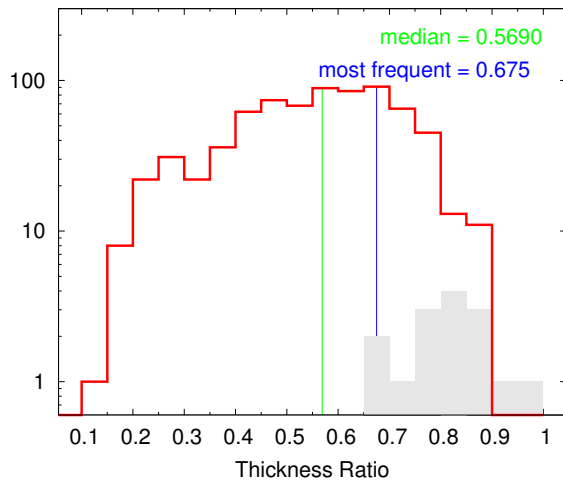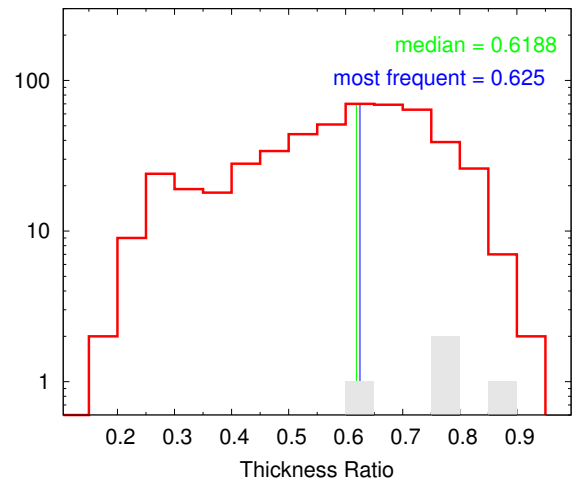

2

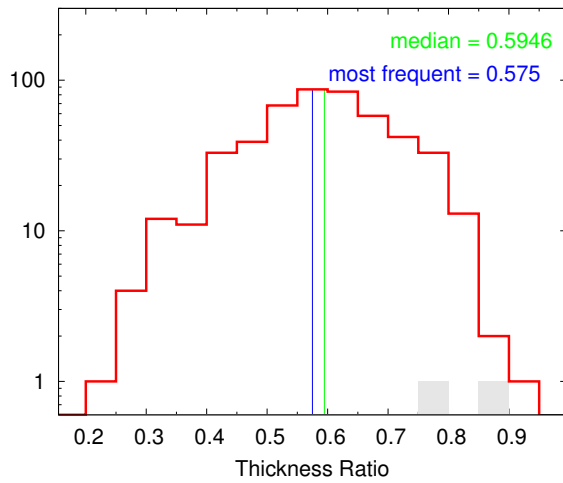

3

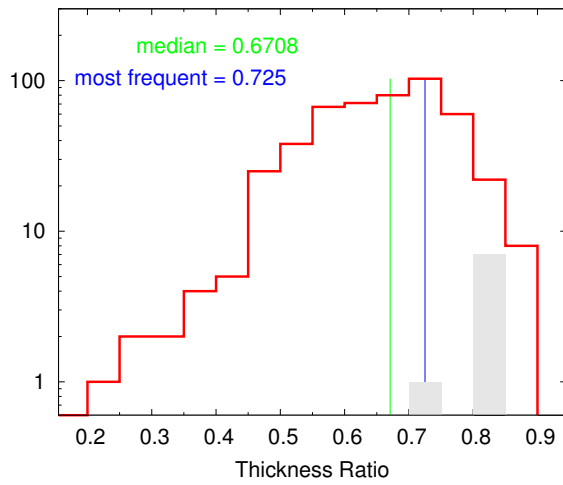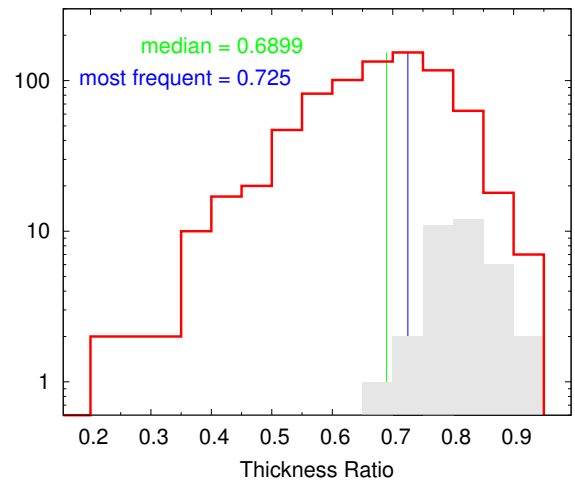

donor

plasma

serum, washed

3

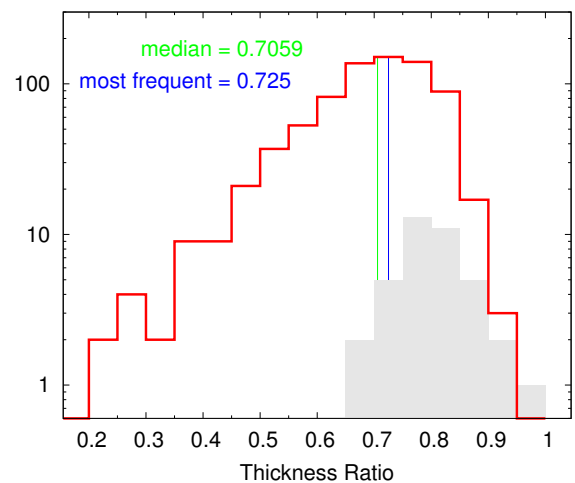

donor

plasma

serum, washed

4

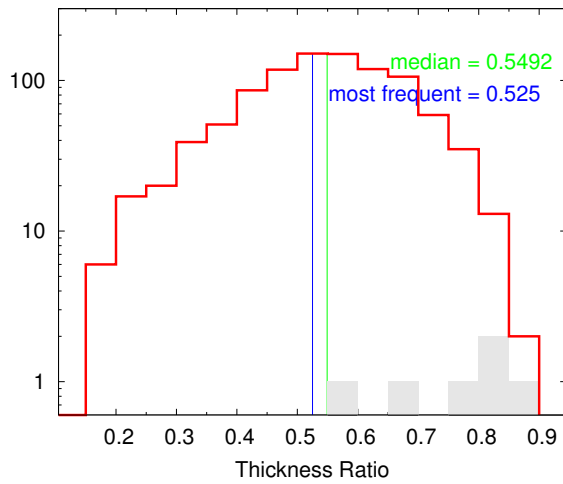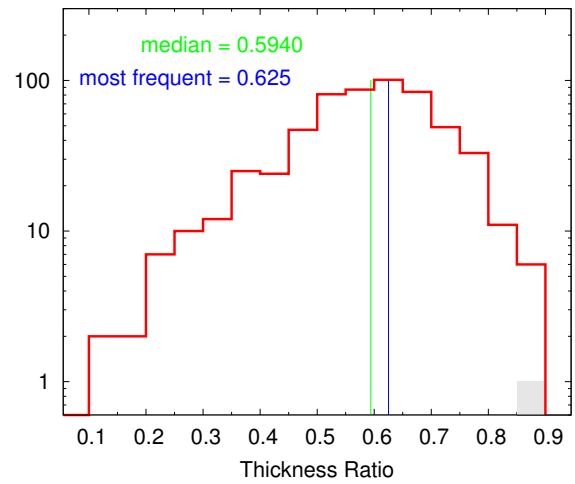

5

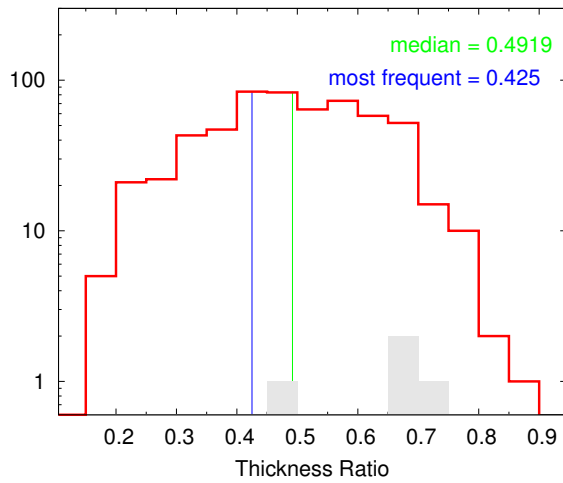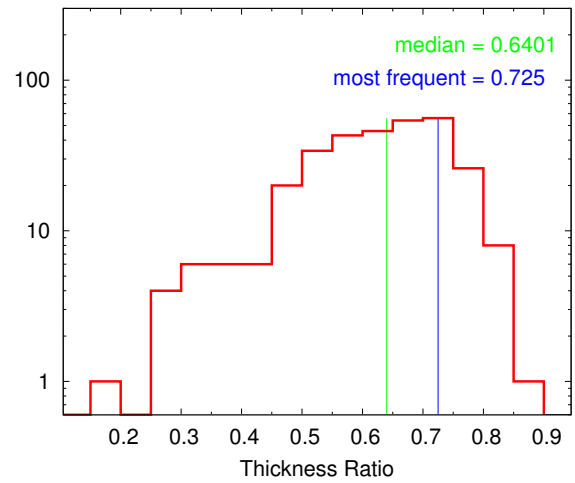

6

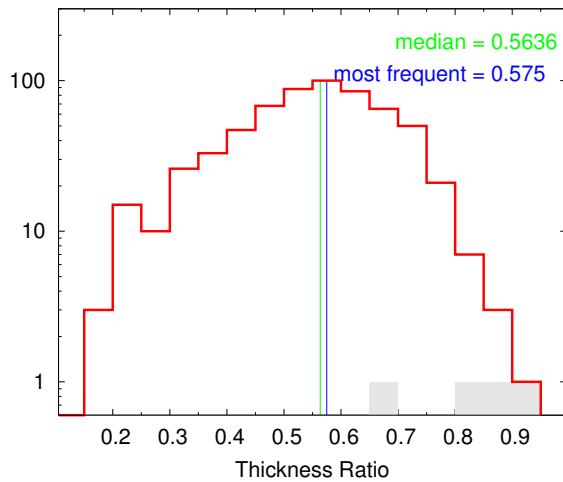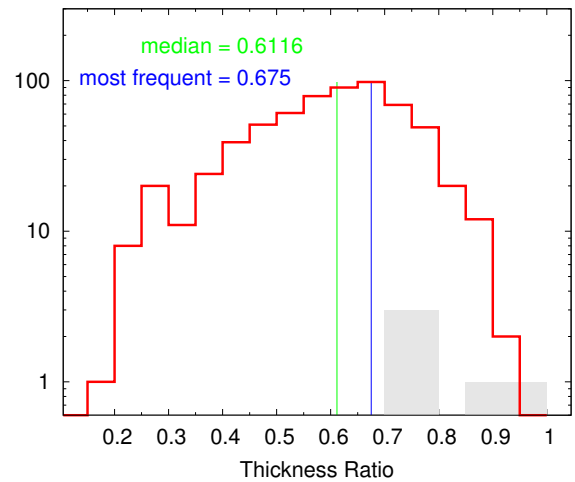

7

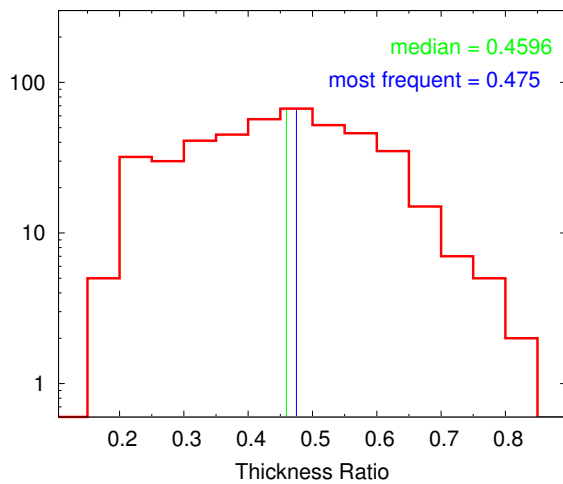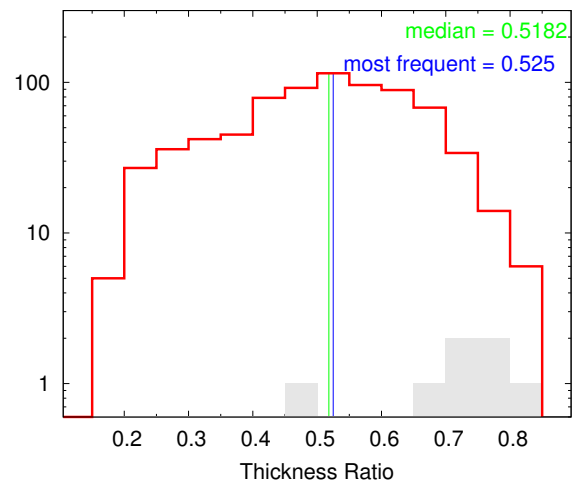

8

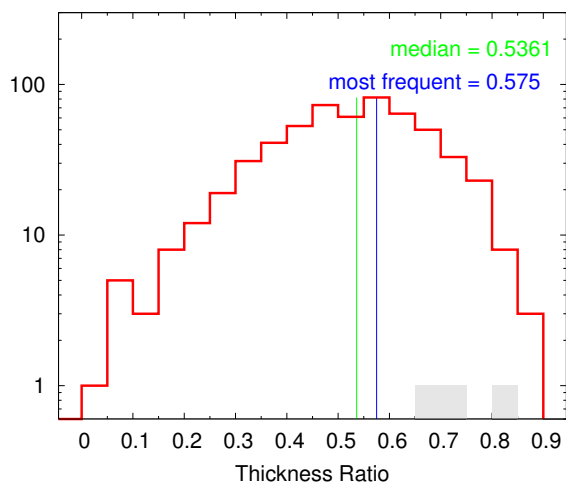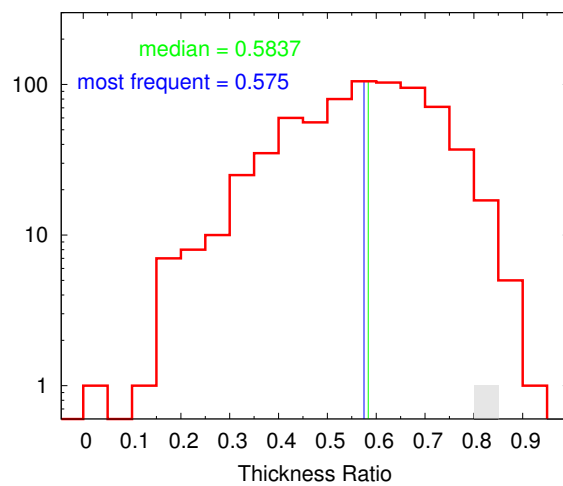

Supplement: Supplementary file 1 [file cells-11-01941-s001.zip › cells-1742284-supplementary/Documents S2-S6/cells-1742284 - Suppl. Document S5.pdf]

donor

plasma

serum, unwashed

1

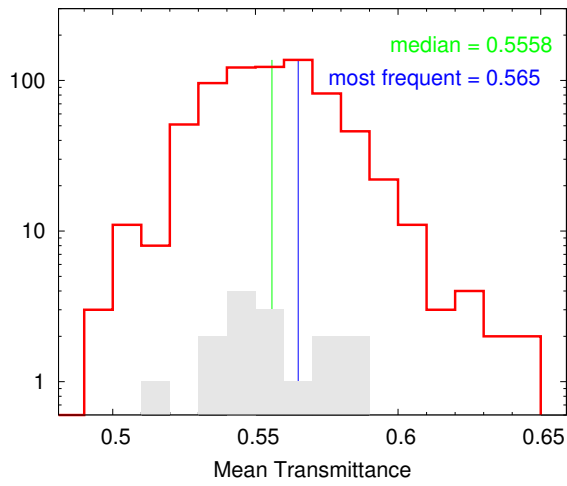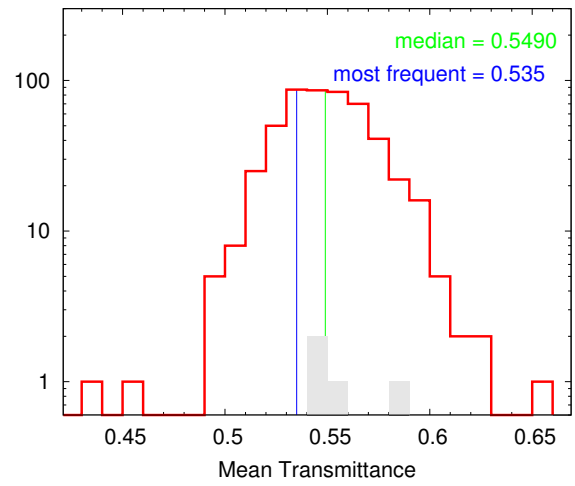

2

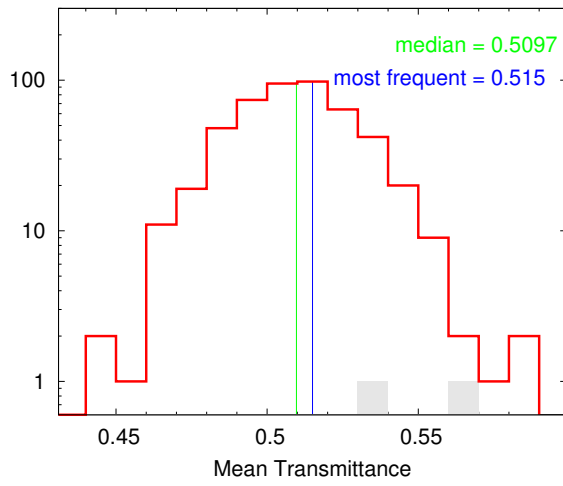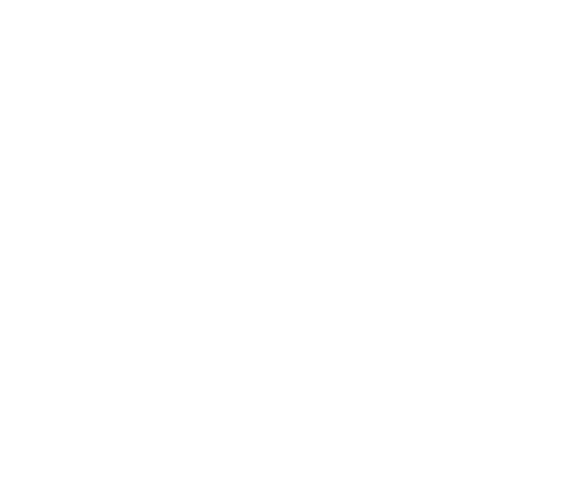

3

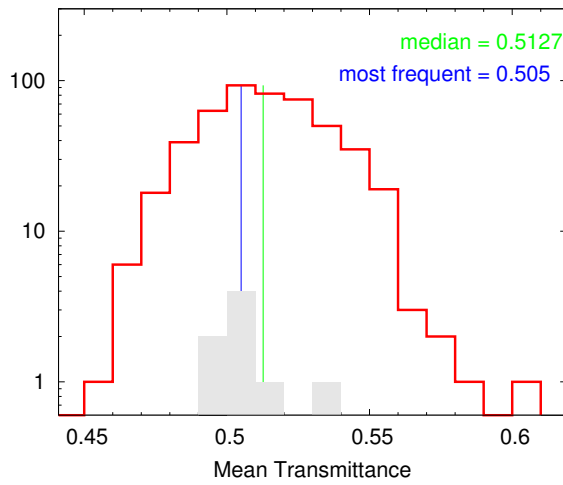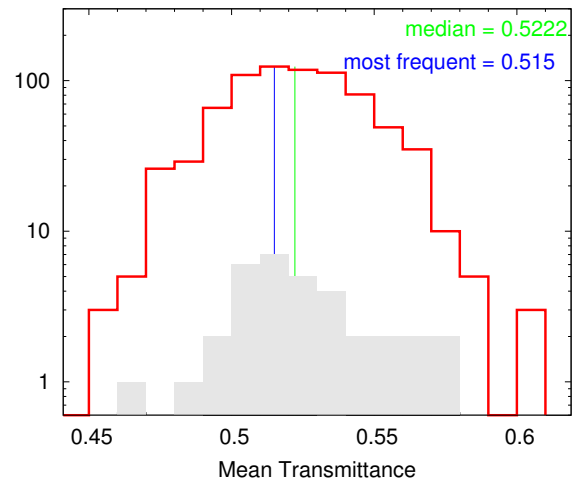

donor

plasma

serum, washed

3

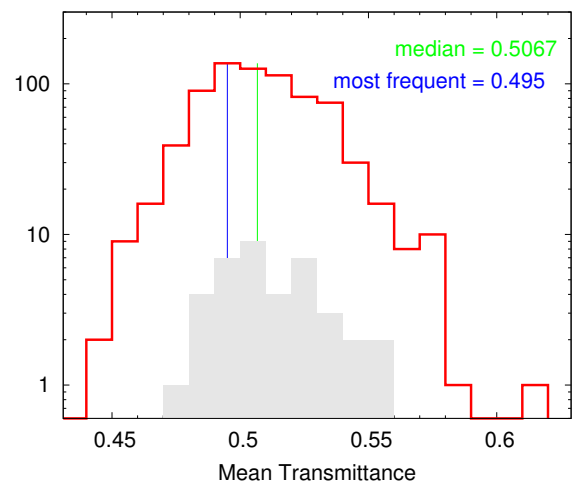

donor

plasma

serum, washed

4

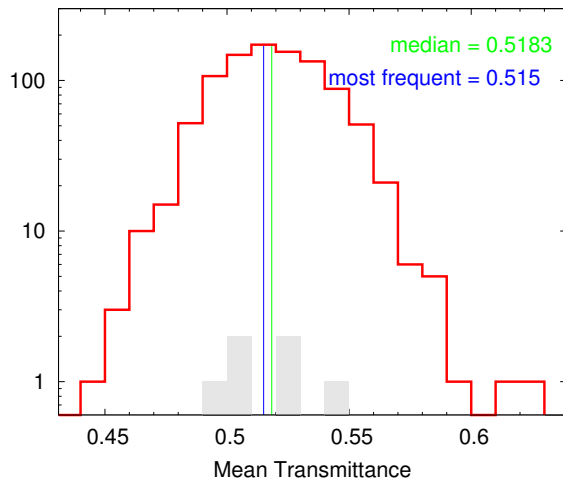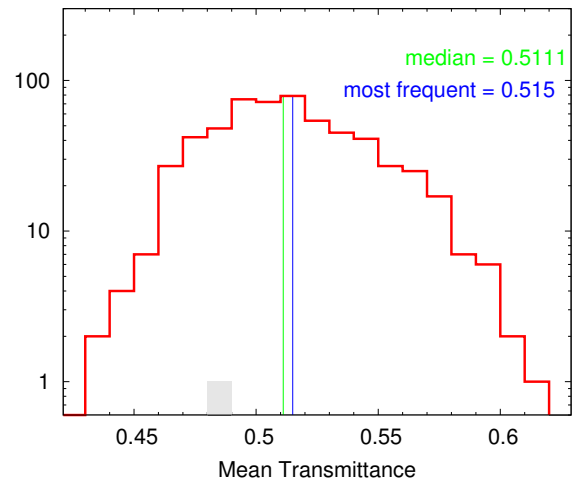

5

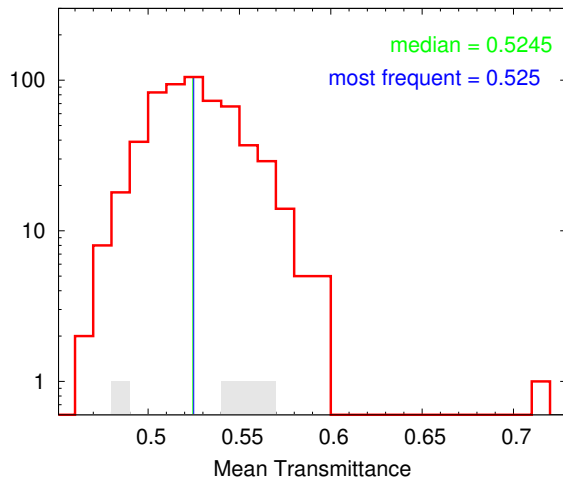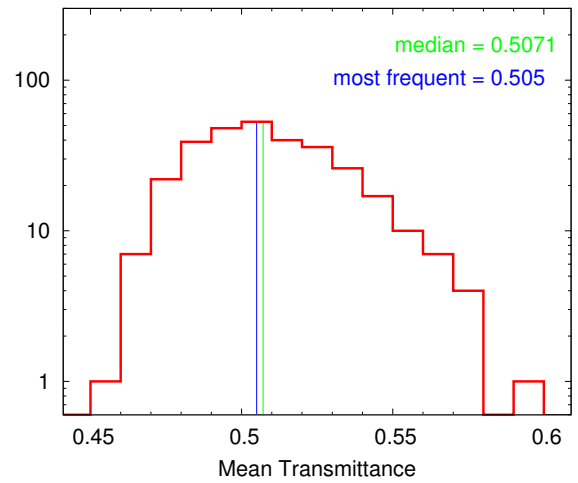

6

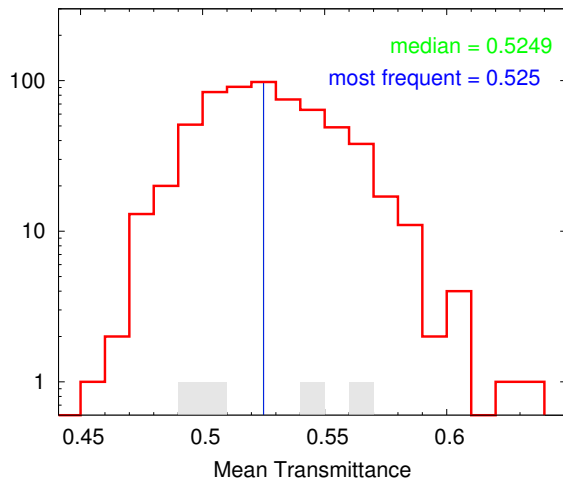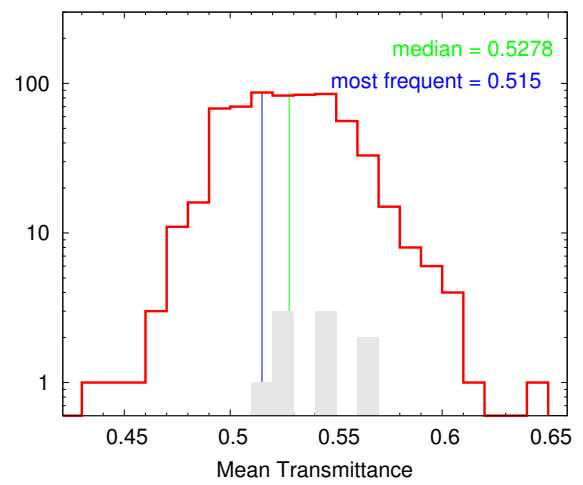

7

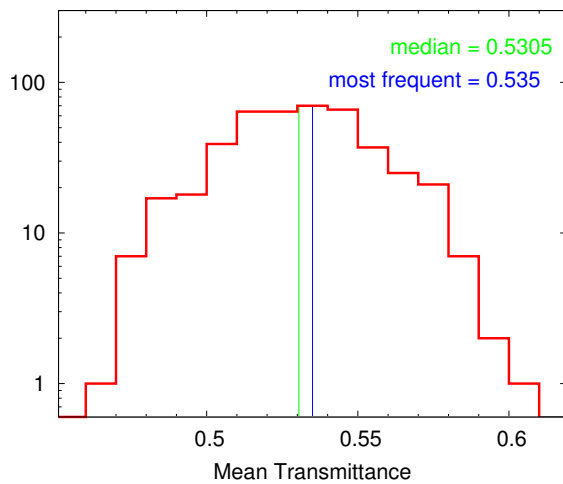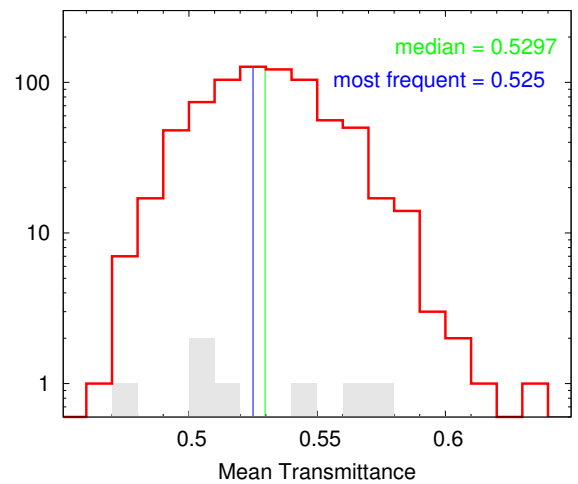

8

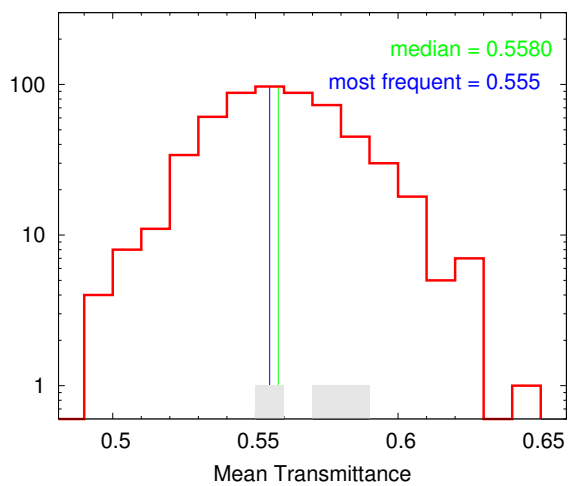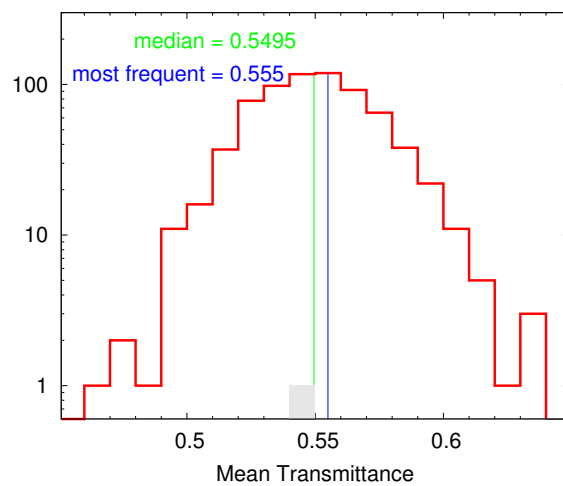

Supplement: Supplementary file 1 [file cells-11-01941-s001.zip › cells-1742284-supplementary/Documents S2-S6/cells-1742284 - Suppl. Document S6.pdf]
